# Electronic spectroscopy and nanocalorimetry of hydrated magnesium ions [Mg(H_2_O)_*n*_]^+^, *n* = 20–70: spontaneous formation of a hydrated electron?†
†Electronic supplementary information (ESI) available. See DOI: 10.1039/c8fd00204e


**DOI:** 10.1039/c8fd00204e

**Published:** 2018-12-10

**Authors:** Thomas Taxer, Milan Ončák, Erik Barwa, Christian van der Linde, Martin K. Beyer

**Affiliations:** a Institut für Ionenphysik und Angewandte Physik , Universität Innsbruck , Technikerstraße 25 , 6020 Innsbruck , Austria . Email: milan.oncak@uibk.ac.at ; Email: martin.beyer@uibk.ac.at

## Abstract

Hydrated singly charged magnesium ions [Mg(H_2_O)_*n*_]^+^ are thought to consist of an Mg^2+^ ion and a hydrated electron for *n* > 15. This idea is based on mass spectra, which exhibit a transition from [MgOH(H_2_O)_*n*–1_]^+^ to [Mg(H_2_O)_*n*_]^+^ around *n* = 15–22, black-body infrared radiative dissociation, and quantum chemical calculations. Here, we present photodissociation spectra of size-selected [Mg(H_2_O)_*n*_]^+^ in the range of *n* = 20–70 measured for photon energies of 1.0–5.0 eV. The spectra exhibit a broad absorption from 1.4 to 3.2 eV, with two local maxima around 1.7–1.8 eV and 2.1–2.5 eV, depending on cluster size. The spectra shift slowly from *n* = 20 to *n* = 50, but no significant change is observed for *n* = 50–70. Quantum chemical modeling of the spectra yields several candidates for the observed absorptions, including five- and six-fold coordinated Mg^2+^ with a hydrated electron in its immediate vicinity, as well as a solvent-separated Mg^2+^/e^–^ pair. The photochemical behavior resembles that of the hydrated electron, with barrierless interconversion into the ground state following the excitation.

## Abstract 2

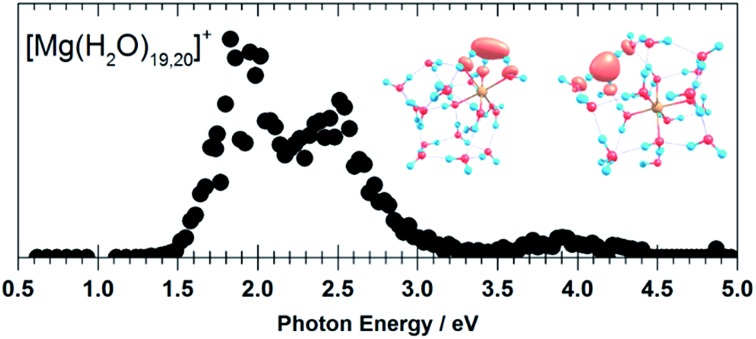
The absorption spectra and photochemistry of [Mg(H_2_O)_*n*_]^+^, *n* = 20–70, resemble those of the hydrated electron (H_2_O)_*n*_^–^.

## I. Introduction

Hydrated metal ions are a useful and well-defined model to examine fundamental chemical reactions including hydrogen production or corrosion mechanisms and to investigate the link from small metal–water complexes to bulk aqueous solutions.^1^–^11^ Among various hydrated metal ions, [Mg(H_2_O)_*n*_]^+^ has received continuous attention.^12^–^40^ Hydrated singly charged magnesium ions can be found in two different species, depending on hydration number. For *n* ≤ 5 and *n* ≥ 17, hydrated magnesium ions [Mg(H_2_O)_*n*_]^+^ are dominant and, in the range of 6 ≤ *n* ≤ 16, hydrated magnesium hydroxide ions [MgOH(H_2_O)_*n*–1_]^+^ are almost exclusively found.^18^,^19^,^28^,^29^,^41^

In our previous work on this topic,^42^ we investigated the photochemistry of small cluster sizes with 1 ≤ *n* ≤ 5, expanding on earlier experimental^15^,^16^,^18^,^19^,^21^,^22^,^28^,^29^ and theoretical^14^,^23^,^24^,^43^ results. In our measured photodissociation spectra, we could assign specific isomers, providing insight into the electronic structure and dissociation mechanisms, which involve water evaporation and hydrogen dissociation.

In the intermediate range of 6 ≤ *n* ≤ 16, Iwata and co-workers proclaimed a negative energy for the hydrogen elimination process for *n* ≥ 6, which explains the switching to [MgOH(H_2_O)_*n*–1_]^+^ in the ion formation in this size region.^23^ The re-switching back to [Mg(H_2_O)_*n*_]^+^ for larger clusters was explained through the formation of a magnesium di-cation and a hydrated electron Mg^2+^(H_2_O)_*n*_^–^ for *n* > 14.^23^ Berg *et al.* also suggested the formation of a solvent separated ion pair of Mg^2+^ and a hydrated electron for *n* > 17.^29^ Siu and Liu proposed an increase in the barrier when the solvated electron moves beyond the third solvation shell as an explanation for the switch off for the hydrogen loss process.^32^ Reinhard and Niedner-Schatteburg proclaimed the formation of a hydrated electron and a magnesium di-cation already for *n* ≥ 8 and suggested the existence of a contact ion pair state for 6 ≤ *n* < 17 and a solvent separated ion pair for *n* ≥ 17.^30^

Here, we focus on the spectroscopy of larger [Mg(H_2_O)_*n*_]^+^ clusters. Hydrated magnesium ions up to *n* = 80 in the ground electronic state were already examined by Berg *et al.*^28^,^29^ who measured black body infrared radiative dissociation (BIRD) rates up to *n* = 41. They let larger clusters fragment down to smaller sizes and found water evaporation as the only dissociation process for *n* ≥ 22 and water evaporation and hydrogen dissociation in the range of 16 ≤ *n* ≤ 21 whereas for *n* ≤ 15, all clusters were transformed into the [MgOH(H_2_O)_*n*–1_]^+^ species. This was argued to happen as a result of the removal of the stabilizing solvent and the recombination of the Mg^2+^(H_2_O)_*n*_^–^ ion pair. We follow up on this work and study photodissociation spectra of the larger [Mg(H_2_O)_*n*_]^+^ clusters, *n* = 20–70. We describe various dissociation pathways and similarities to the behavior of the hydrated electron, (H_2_O)_*n*_^–^. Experimental results are supported by *ab initio* calculations.

## II. Methods

A detailed description of the experimental setup can be found in earlier works.^44^–^47^ A Fourier Transform Ion Cyclotron Resonance Mass Spectrometer (FT-ICR-MS), equipped with a 4.7 T superconducting magnet was used to conduct the experiments. Mg^+^ ions are generated in an ion source *via* laser vaporization of an isotopically enriched (99% ^24^Mg) rotating target disk, using the 2^nd^ harmonic of a Nd:YAG laser. The ions then are entrained by a gas pulse of helium seeded with water vapor at 20 bar and expanded into high vacuum which leads to cluster formation of [Mg(H_2_O)_*n*_]^+^. The ions are stored and detected in a liquid nitrogen cooled ICR cell (*T* ∼ 90 ± 5 K) to minimize the influence of blackbody infrared radiative dissociation (BIRD).^48^–^58^ Ion selection is performed *via* the resonant ejection of ions with an unwanted mass to charge ratio. Because of the relatively high BIRD fragmentation rates for these large cluster sizes, a combination of [Mg(H_2_O)_*n*_]^+^ and [Mg(H_2_O)_*n*–1_]^+^ (the primary BIRD fragment) was selected for the experiments. Also present [MgOH(H_2_O)_*n*–1,*n*–2_]^+^ species were not ejected to avoid unintentional excitation of the ions of interest to higher cyclotron radii, thus diminishing their overlap with the dissociation laser beam. The presence of these species does not influence our results as they do not absorb photons in the wavelength range of interest (see Section 4 in the ESI† for details). Photodissociation is induced *via* irradiation with the beam of a tunable wavelength, pulsed (20 Hz) UV/VIS/NIR laser system (Nd:YAG pumped OPO system EKSPLA NT342 B-20-SH-SFG) with irradiation times of up to 1 s. A more detailed description of the laser setup is available elsewhere.^59^

Due to the high absorption cross section of [Mg(H_2_O)_*n*_]^+^, sequential absorption of several photons is frequently observed. Similar to earlier work,^60^,^61^ the average water binding energy was extracted from the photodissociation data using the equation the average water binding energy was extracted from the photodissociation data using the equation 〈*E*_H_2_O_〉 = = *E*_*γ*_*γ*/*m*. Here, *E*_*γ*_ is the photon energy for which the partial cross section of a specific fragmentation channel [Mg(H_2_O)_*n*_]^+^ → [Mg(H_2_O)_*n*–*m*_]^+^ + (H_2_O)_*m*_ reaches its maximum (see Section 6 in ESI† for details). This energy is then multiplied by the number of photons *γ* involved in the dissociation and divided by the number of lost water molecules *m*.

The photon flux inside the cell is strongly dependent on the wavelength, which is a typical feature for OPO systems. In our experiments, it was on the order of 0.25–7 mJ cm^–2^. Directly after irradiation, mass spectra of fragment and parent ions were recorded. From the parent and fragment intensities, along with the laser power, the relative photodissociation cross sections were calculated using Lambert–Beer’s law (see Section 1 in the ESI†).

The photochemistry of the [Mg(H_2_O)_*n*_]^+^, *n* = 3–8, 10, 12, 16 and 20 clusters was modeled using theoretical chemistry methods, with four types of clusters considered: with a three-, four-, five- and six-times coordinated Mg center (denoted also as 3×, 4×, 5×, and 6×; see Fig. S3† for all the structures considered). The ground state structures of the clusters were optimized at the ωB97XD/def2TZVP level of theory.^62^ Excited states were calculated using time-dependent density functional theory (TDDFT) with the CAM-B3LYP functional,^63^ the complete active space – self consistent field (CASSCF) method with subsequent multi-reference configuration interaction (MRCI), and the algebraic diagrammatic construction to the second order (ADC(2))^64^ method. The ADC(2) method was used for benchmarking the CAM-B3LYP calculations. The results are shown in Fig. S4.† For the excited state calculations, the aug-cc-pVDZ basis set was used. It was shown previously that this basis is able to correctly capture the first three excited states that are most relevant for the present study.^42^ Quantitative differences for higher-lying states might be expected. Excited states up to 5 eV or to a limit of 35 excited states were considered.

[Mg(H_2_O)_*n*_]^+^ clusters were created by the sequential addition of water molecules and re-optimization. For the [Mg(H_2_O)_12_]^+^ cluster, molecular dynamics (MD) was used to sample the potential energy surface for the structure search at the BLYP/6-31+g* level at 400 K with a time step of 30 a.u. (∼0.73 fs). After a thermalization period of 3 ps, a production run of 11 ps was conducted, with 12 structures taken for reoptimization at the ωB97XD/def2TZVP level.

The Gaussian program^65^ was used for ground-state and TDDFT calculations, Molpro^66^ for CASSCF/MRCI, and Turbomole^67^ for ADC(2). The spectrum width was modeled using the linearized reflection principle.^68^–^71^ MD calculations were performed in the Abin program.^72^

## III. Results and discussion

**Fig. 1:**
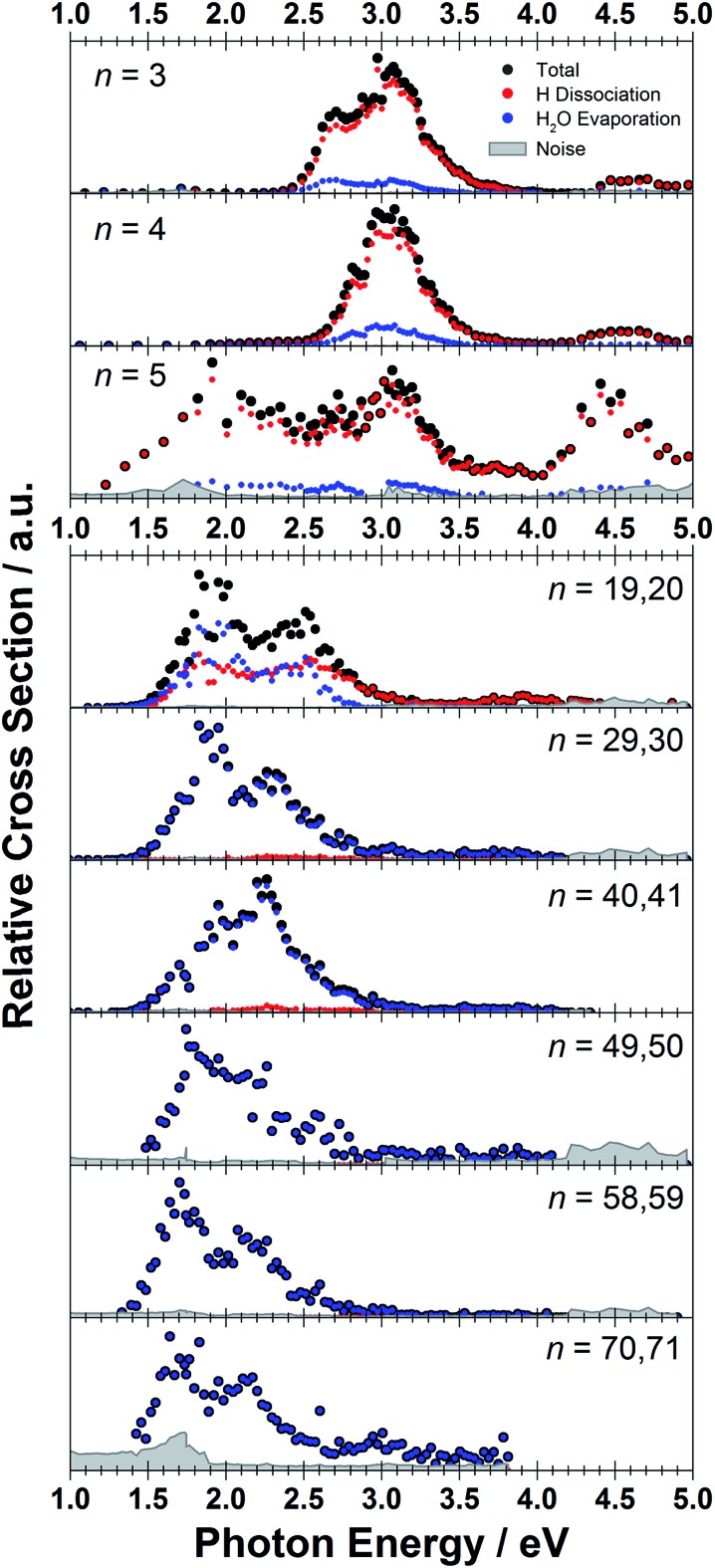
Experimentally measured relative photodissociation cross sections of [Mg(H_2_O)_*n*_]^+^ ions for 3 ≤ *n* ≤ 5 (adapted from ref. 42) and *n* ∼ 20, 30, 40, 50, 60 and 70, with partial cross sections for fragmentation *via* only the loss of water molecules (blue) and the additional loss of atomic hydrogen (red).

The measured photodissociation spectra for [Mg(H_2_O)_*n*_]^+^, *n* ∼ 20, 30, 40, 50, 60 and 70 at 90 ± 5 K are shown in Fig. 1. Spectra for small clusters for *n* = 3–5 recorded at a temperature of 130 ± 20 K, which were already discussed elsewhere,^42^ are shown for comparison. The spectra for *n* = 1 and 2 show two separated peaks, corresponding to the 3s–3p_*x*,*y*_ and 3s–3p_*z*_ electronic transitions of the isolated magnesium cation that are perturbed by the influence of the water molecules.^42^ For *n* ≥ 3, these transitions are not separated any more. The spectra for *n* = 1–3 show a significant redshift, caused by the increasing coordination of the magnesium cation with water molecules. The spectrum for *n* = 3 also shows the 3s–3d/4s electronic transitions at the higher energy end at about 4.5 eV. For *n* = 4, the redshift stops, indicating that the observed isomer is still a triply coordinated magnesium cation with a fourth water molecule already in the second solvation shell. For *n* = 5, we assume three different isomers contributing to the broad shape of the spectrum, a three-, four- and five times coordinated magnesium cation.

In the range of *n* ∼ 6–18, magnesium hydroxide clusters [MgOH(H_2_O)_*n*–1_]^+^ are predominantly formed in the ion source. Therefore, no photodissociation spectra could be measured (see also Section 4 in the ESI†). The hydroxide formation reaction is suppressed again for *n* > 18.^28^,^29^

While the photodissociation spectra change substantially with every coordinated water molecule for small clusters of *n* = 1–5, the spectra of larger clusters are very similar in the whole *n* = 20–70 range explored in this study, with only limited changes in the position and shape with the increasing size of the clusters. The spectrum for *n* ∼ 20 shows a broad absorption with a width of about 1.8 eV spanning from 1.4 eV to 3.2 eV, with two distinguishable bands peaking around 1.9 eV and 2.5 eV. There is also a long shallow tail on the higher energy side of the band, extending up to about 4.4 eV before vanishing in the noise level.

Compared to the spectrum for *n* ∼ 20, the higher-energy peak for *n* ∼ 30 is shifted to the red by about 0.2 eV, with the redshift continuously increasing with more water molecules to about 0.4 eV for *n* ∼ 70. The lower-energy peak for *n* ∼ 30 is at about the same position as for *n* ∼ 20, and shifts slightly to the blue for *n* ∼ 40 and back to the red for *n* > 40. For *n* ∼ 30, 40, 50 and 70 there also seems to be a third, less intense band at ∼3.0 eV.

**Fig. 2:**
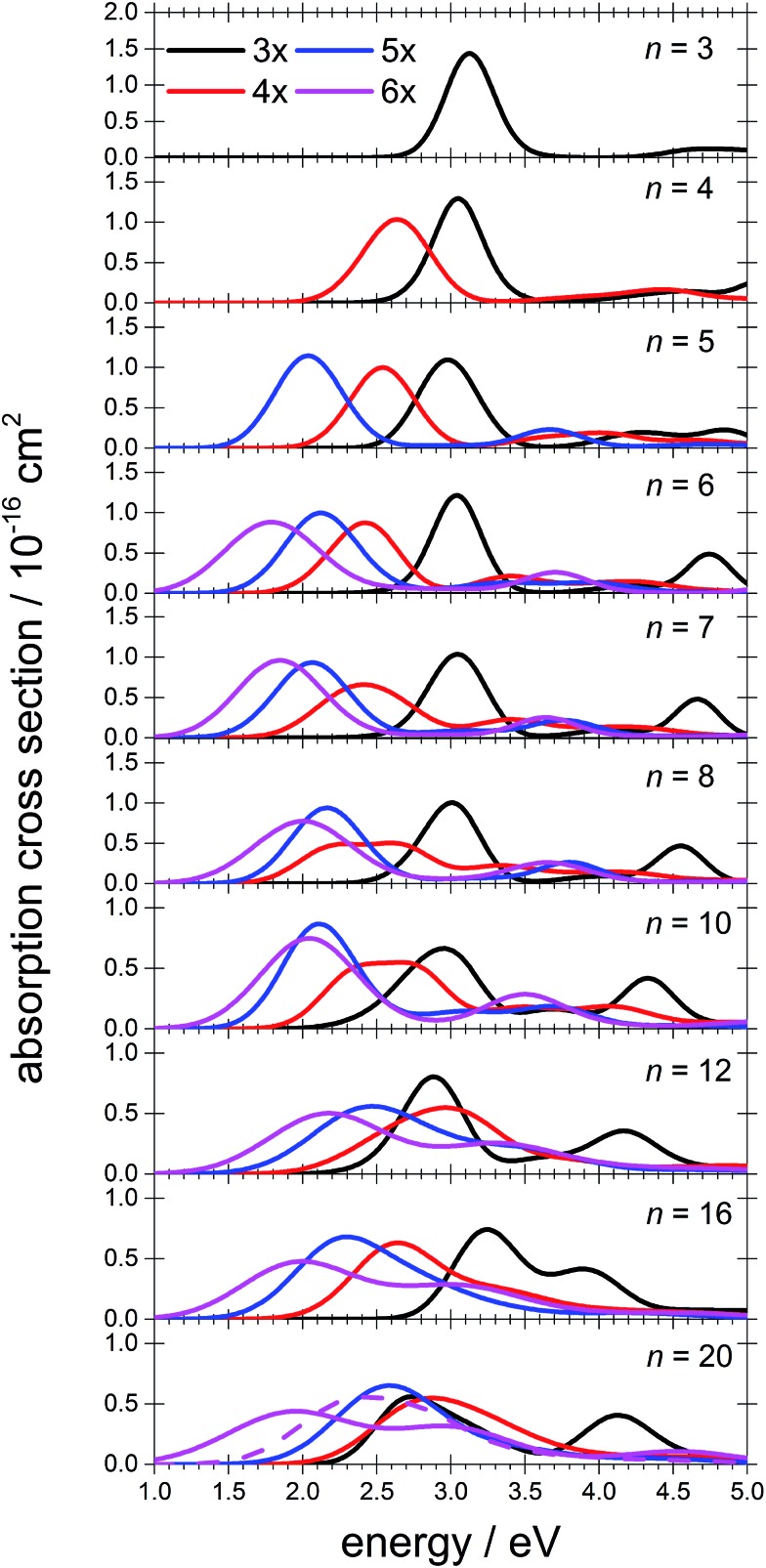
Calculated spectra of selected [Mg(H_2_O)_*n*_]^+^ ions with different Mg coordination numbers (3×–6×). Calculated at the CAM-B3LYP/aug-cc-pVDZ//ωB97XD/def2TZVP level of theory using the linearized reflection principle to calculate the peak width. Surface and inner isomers for *n* = 20 are shown with full and dashed lines, respectively. Benchmarking with respect to the ADC(2) method can be found in Fig. S4.†

**Fig. 3:**
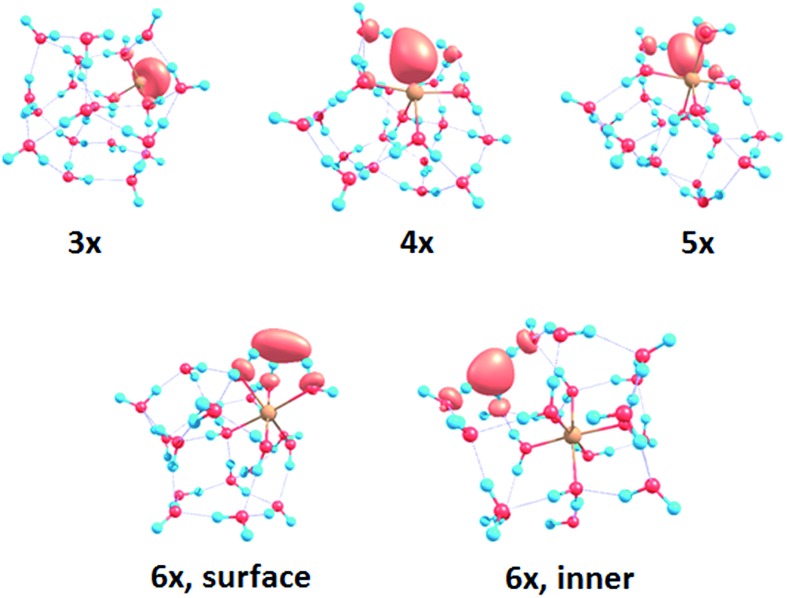
Structures for *n* = 20 along with the spin density calculated at the CAM-B3LYP/aug-cc-pVDZ//ωB97XD/def2TZVP level. See Fig. S1† for other structures.

To interpret the observations, we modeled the spectra with *ab initio* calculations, using various [Mg(H_2_O)_*n*_]^+^ structures, *n* = 3–20, with a three-, four-, five- and six-fold coordinated Mg ion (Fig. 2). Due to the size of the system, we have to limit ourselves to a relatively small selection of structures. However, we observe clear trends in the evolution of the photodissociation spectrum as we discuss below. Consistently with previous studies,^30^ we predict that the electron delocalizes with increasing coordination as can be documented *e.g.* for *n* = 20 where the gyration radius increases from 1.9 Å to 2.4 Å for three- and six-fold coordinated Mg ions, respectively (see also Fig. 3). For coordination numbers 3–5, the electron is localized next to the Mg ion. For a coordination number of 6, we considered two [Mg(H_2_O)_20_]^+^ isomers, with the [Mg(H_2_O)_6_]^+^ unit on the surface and hydrated in the cluster. In these two structures, the electron resides next to the Mg^2+^ ion or is shifted to the second solvation layer in surface and inner structures, respectively.

The calculated photoabsorption spectra in Fig. 2 show that the Mg coordination number has the most important influence on the spectrum shape, with hydration in further solvation layers inducing only a minor shift of the absorption bands. For a three-fold coordinated magnesium, the first absorption band composed of s–p transitions peaks at about 3.2 eV for *n* = 3. With an increasing number of water molecules, its maximum stays within 2.8–3.4 eV and shifts due to interactions in the second solvation layer. There is a distinct second absorption band that shifts to a lower energy with increasing size and can be assigned to transitions of 3s–3d and 3s–4s character.

For other coordination numbers of the Mg ion, we see a similar trend for the first absorption band, with only a limited change with increasing hydration, with the shift of the maximum within 0.5 eV. The same order of the excitation energies, *i.e.* lower excitation energies for clusters with a higher Mg coordination number, is preserved for all cluster sizes with the exception of three- and four-fold coordinated clusters for *n* = 20. The second absorption band localized in the 2.5–4.5 eV region shifts to lower energies with increasing cluster size. At the same time, the spectral width calculated within the linearized reflection principle is predicted to increase for larger clusters. With respect to the experimental spectra, the bands for six-fold coordinated Mg at ∼1.9 eV and ∼3.0 eV are less separated and the spectra are calculated to already start at 1.0 eV (compared to ∼1.5 eV in the experiment). This indicates that the width is overestimated by the linearized reflection principle.

For the surface isomer with *n* = 20, the absorption maximum is calculated at 2.0 eV, consistently with other clusters with a six-fold coordinated Mg ion. When the unpaired electron is shifted to the second solvation layer with full hydration of the [Mg(H_2_O)_6_]^+^ unit, a shift of ∼0.4 eV to the blue is seen in the calculated spectrum. This shift is preserved in the whole *n* = 17–20 series as we show in the ESI (Fig. S5).†

Comparing the calculated photoabsorption spectra to the measured photodissociation spectra, we can propose two interpretations of the results. We could assign the two observed peaks in the experiment to five-fold and six-fold coordinated magnesium ions on the cluster surface. This would indicate that the coordinated Mg ion stays on the surface of the cluster in the whole investigated size range. Alternatively, we could see the six-fold coordinated Mg ion in its two forms, *i.e.* with the unpaired electron in the vicinity of the Mg^2+^ ion and in the second hydration layer.

**Fig. 4:**
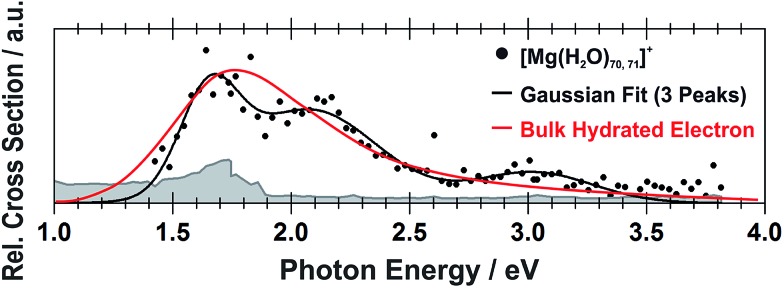
Comparison between the photodissociation spectrum of [Mg(H_2_O)_70,71_]^+^ and the spectrum of a bulk hydrated electron.^73^

Still another possible interpretation of the experimental spectra would be to assign the two bands to two and one s–p transitions of the hydrated electron, respectively, similarly to clusters with *n* = 1 and 2. A comparison of the spectrum of the largest measured cluster with the spectrum of a hydrated electron in bulk water^73^ is provided in Fig. 4. Both spectra show significant congruence and this could be interpreted as an indication that the [Mg(H_2_O)_*n*_]^+^ ions exist in the long-assumed^23^,^29^,^30^ Mg^2+^(H_2_O)_*n*_^–^ state for clusters with *n* ≥ 20 water molecules with a well separated hydrated electron, without any significant influence by the coordination number of the Mg^2+^ ion. The presence of Mg^2+^ would influence the electron only indirectly by disrupting the network of hydrogen bonds^74^ compared to pure (H_2_O)_*n*_^–^ clusters. This assignment is however not supported by our calculations for *n* = 20 in which the three electronic transitions forming the first band have a spread of only 0.5 eV and are predicted to merge into one band within the linearized reflection principle approach.

Next, we discuss the photodissociation pathways. Following excitation, we observe two different fragmentation channels, one involving pure H_2_O evaporation (I) and the other also showing H dissociation (II):

I [Mg(H_2_O)_*n*_]^+^ → [Mg(H_2_O)_*n*–*m*_]^+^ + (H_2_O)_*m*_

II [Mg(H_2_O)_*n*_]^+^ → [MgOH(H_2_O)_*n*–*m*–1_]^+^ + (H_2_O)_*m*_ + H

**Fig. 5:**
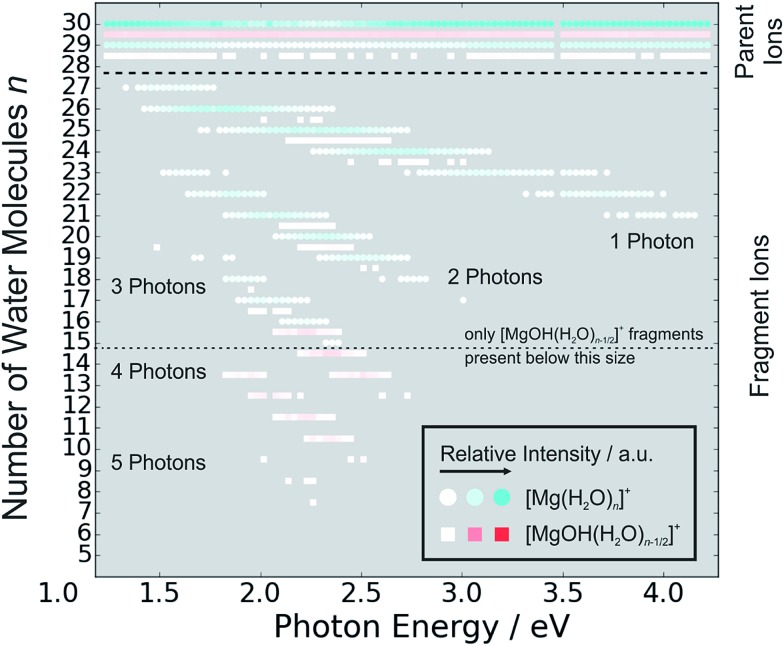
Fragmentation pattern from a typical photodissociation experiment for [Mg(H_2_O)_29,30_]^+^. The two different species of hydrated magnesium ions are represented by blue dots for [Mg(H_2_O)_*n*_]^+^ ions and red squares for [MgOH(H_2_O)_*n*–1/2_]^+^ ions. Color intensity represents measured ion intensity.

Fig. 5 shows the fragmentation pattern from a typical experiment for [Mg(H_2_O)_29,30_]^+^, with fragmentations involving the absorption of up to five photons (see Section 3 in the ESI† for detailed spectra of all the ions). The energetics of channel (I) in the experiment were analyzed through nanocalorimetry.^44^ We found that the average energy carried away by a water molecule over all cluster sizes is about 0.47(3) eV (Table 1), close to the literature value for the binding energy of a water molecule to a large ionic cluster (for *n* ≥ 40) of about 0.447(4) eV.^75^ In other words, the absorbed photon energy is quantitatively used for water dissociation, indicating that dissociation takes place in the electronic ground state of the system. Following the excitation, the system thus reaches a conical intersection and dissociates water molecules after funneling into the ground electronic state. For hydrated electrons, it was experimentally established by the Neumark group that internal conversion to the ground state takes place on a sub-ps timescale.^76^ Note that this is a completely opposite behavior to the one observed for *n* = 1–5.^42^ We observe cases where more photons are absorbed than laser pulses applied during the irradiation time window, which means that several photons must be absorbed during one laser pulse. As we will discuss below, it can be expected that internal conversion takes place on the picosecond time scale. Thus, within the 5 ns of a typical laser pulse, it is possible for a cluster ion to absorb one or more photons in the electronic ground state. As the dissociation of water molecules may take place in less than 5 ns, fragment ions created with more than a single photon could be direct fragments of the precursor ions as well as secondary products.

**Table 1:** Average binding energy per water molecule Average binding energy per water molecule 〈*E*_H_2_O_〉 (in eV) (in eV)

*n*	*γ*				
	1	2	3	4	5
20	0.51	—	—	—	—
30	0.46	0.46	0.47	—	—
41	0.49	0.48	0.47	0.47	0.48
50	0.45	0.45	0.47	0.47	0.47
59	0.47	0.46	0.46	0.46	0.47
71	0.43	0.45	0.46	0.46	0.47

Hydrogen atom dissociation, the second channel (II), produces [MgOH(H_2_O)_*n*_]^+^ ions that do not absorb further photons in the considered wavelength range. Below a limit of *n* = 15 water molecules, only [MgOH(H_2_O)_*n*_]^+^ ions are observed as fragmentation products whereas above this value, [Mg(H_2_O)_*n*_]^+^ ions are the dominant species (Fig. 5). The formation of magnesium hydroxide species in this range agrees quantitatively with the behavior of [Mg(H_2_O)_*n*_]^+^ clusters in the ground state investigated in previous mass spectrometric experiments.^18^,^19^,^28^,^29^ Therefore, we conclude that the hydrogen dissociation reaction (II) also takes place in the ground electronic state.

To calculate the partial cross sections of H_2_O evaporation and H dissociation in Fig. 1, only [MgOH(H_2_O)_*n*_]^+^ products created with the first absorbed photon are considered for the H dissociation channel and all fragments created by multiple photons are counted for the H_2_O evaporation channel. For *n* = 20, the partial cross sections of the two fragmentation pathways are about the same order of magnitude, but larger clusters fragment almost exclusively *via* the loss of water molecules.

To analyze the photochemistry of the [Mg(H_2_O)_*n*_]^+^ ions, we investigated photochemical pathways using theoretical chemistry methods. When a [Mg(H_2_O)_*n*_]^+^ cluster is excited into one of the three close-lying electronic states (D_1_–D_3_), we can expect that it quenches to the D_1_ state and evolves further on this potential energy surface. For the possible transfer back into the ground state as seen in the experiment, the size of the D_0_/D_1_ gap is crucial. We have already shown that for the [MgH_2_O]^+^ ion, the D_0_/D_1_ gap is almost identical to the D_0_–D_1_ excitation energy due to negligible geometry relaxation following the excitation.^42^ For *n* = 2 and 3, we already saw considerable relaxation with a tendency to linearize or planarize, decreasing the gap.^42^

**Fig. 6:**
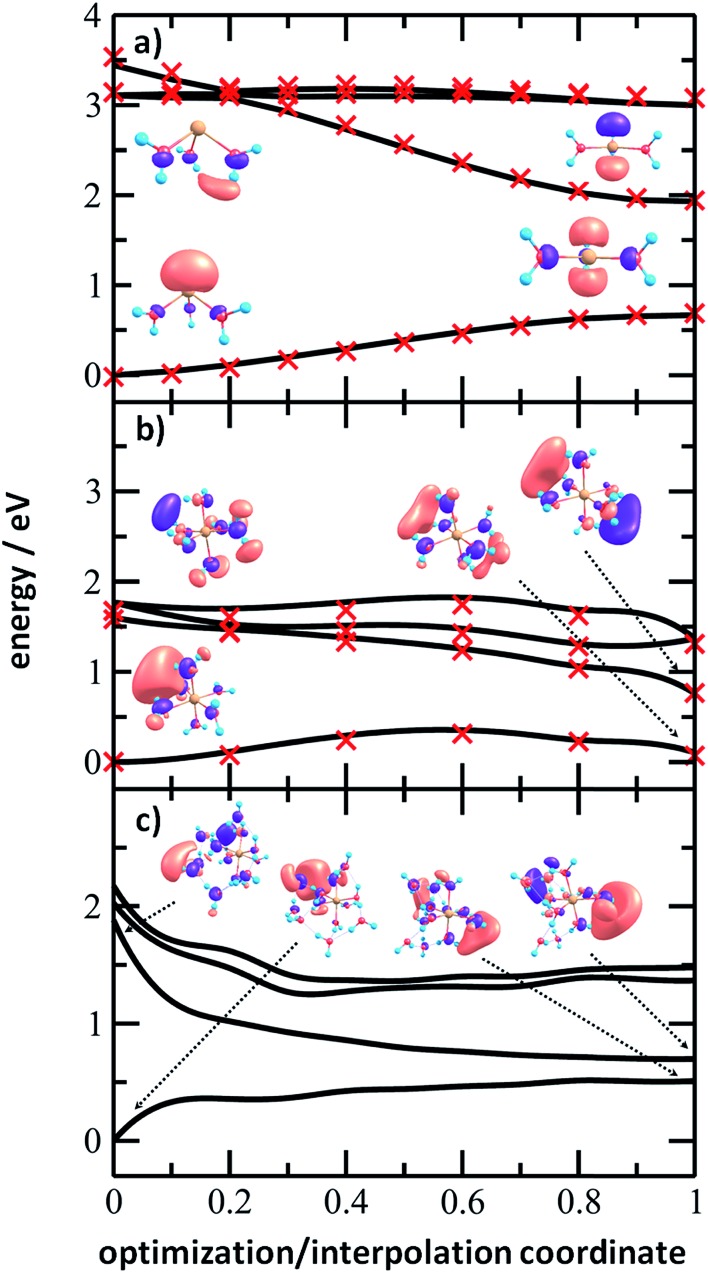
Potential energy scans for (a) [Mg(H_2_O)_3_]^+^, (b) [Mg(H_2_O)_6_]^+^ and (c) [Mg(H_2_O)_12_]^+^ ions calculated at the ADC(2)/aug-cc-pVDZ level of theory, connecting the ωB97XD/def2TZVP minimum in the ground state and the D_1_ excited state minimum optimized at the ADC(2)/aug-cc-pVDZ level, either by interpolation between the structures (a and b) or by following the ADC(2) optimization pathway (c). Natural transition orbitals calculated at the CAM-B3LYP/aug-cc-pVDZ level are shown for the first and last points. In (a) and (b), single-point recalculations are provided as crosses at the MRCI(3,5)/aug-cc-pVDZ and MRCI(1,5)/aug-cc-pVDZ levels, respectively.

Fig. 6 shows the evolution of the photochemical pathways for [Mg(H_2_O)_*n*_]^+^, *n* = 3, 6 and 12. For [Mg(H_2_O)_3_]^+^, optimization in the first excited state D_1_ leads to planarization. This can be understood in terms of optimizing the molecular structure for the 3p(Mg) orbital in which the electron is localized after excitation. The D_0_/D_1_ gap in the D_1_ minimum is about 1.3 eV. For [Mg(H_2_O)_6_]^+^, only the OH bond orientation changes to provide a more suitable environment for an orbital of p character delocalized over the whole cluster. Calculations yield a D_0_/D_1_ gap of 0.6 eV.

For [Mg(H_2_O)_12_]^+^, as a larger cluster with six-fold coordinated magnesium, the gap reduces to 0.2 eV due to the extended cluster flexibility, again heading for a structure with a fully accommodated delocalized p orbital. Note that all the calculated D_1_ minima can be reached from the Franck–Condon region without the need to surpass a barrier, and we can therefore expect that the transition to the D_1_ minimum takes place on a picosecond timescale.

The D_0_/D_1_ gap values should be compared to the D_0_/D_1_ gap in the D_1_ minimum of (H_2_O)_6_^–^, calculated as 0.23 and 0.25 eV at the ADC(2)/aug-cc-pVDZ and MRCI(3,5)/aug-cc-pVDZ levels of theory, respectively. The small water cluster is already flexible enough to provide an environment for structural changes. The presence of the magnesium ion, on the other hand, influences the structure and a low value of the D_0_/D_1_ gap can be reached only when more water molecules are present.

**Fig. 7:**
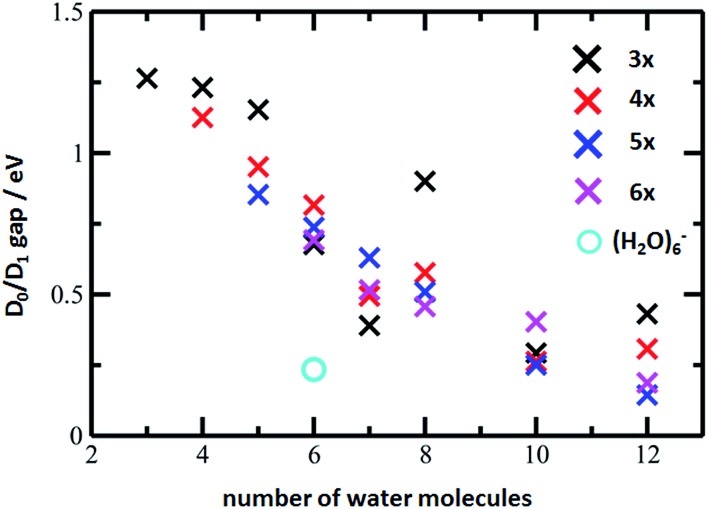
The D_0_/D_1_ gaps as calculated in the D_1_ minimum for the [Mg(H_2_O)_*n*_]^+^ clusters, *n* = 3–12, (crosses) and (H_2_O)_6_^–^ (circle). Calculated at the ADC(2)/aug-cc-pVDZ level of theory.

To further support this argument, we calculated the evolution of the D_0_/D_1_ gap for several [Mg(H_2_O)_*n*_]^+^ clusters (Fig. 7). It can be seen that with an increasing size of cluster, the D_0_/D_1_ gap decreases. Starting with seven water molecules, the gap drops below 0.5 eV and might already reach values below 0.25 eV for *n* = 12 (see Fig. S6† for optimized structures).

We can thus propose that the photochemistry of larger [Mg(H_2_O)_*n*_]^+^ clusters is similar to the photochemistry of an electron on a water cluster. After excitation, they reach a D_1_ minimum without any barrier and switch back to the D_0_ ground electronic state that lies energetically close to D_1_ in this region. Then, the clusters follow the known ground state reactivity,^28^,^29^ *i.e.* evaporate water molecules or dissociate a hydrogen atom to produce hydrated magnesium hydroxide for lower values of *n*.

## IV. Conclusions

Hydrated magnesium ions [Mg(H_2_O)_*n*_]^+^ exhibit a broad electronic absorption spectrum from 1.4–3.2 eV, in the same range as the hydrated electron. The spectrum is, however, more structured, with two pronounced local maxima and possibly even a third one. Based on quantum chemical calculations, these maxima can be assigned to different structural motifs, in particular five- and six-fold coordinated magnesium, or six-fold coordinated magnesium with two distinct hydration patterns. Due to the vast conformation space of these species, however, only a limited number of structures could be investigated computationally, which does not allow for a definitive assignment of the observed features. After excitation, the excitation energy is quantitatively used for dissociative reactions. We propose that the D_1_ minimum is reached on a picosecond timescale and the system might switch to the D_0_ ground state due to a low value of the D_0_/D_1_ gap in the vicinity of the D_1_ minimum for larger clusters. This resembles the photochemistry of the hydrated electron in (H_2_O)_*n*_^–^ clusters.

## Conflicts of interest

There are no conflicts of interest to declare.

## Supplementary Material

Supplementary information
