# Supplementary material for: Electronic spectroscopy and nanocalorimetry of hydrated magnesium ions [Mg(H2O)n]+, n = 20–70: spontaneous formation of a hydrated electron?
Source: Faraday Discuss. 2018 Dec 10;217(0):584–600. doi: 10.1039/c8fd00204e (PMC6677030; doi:10.1039/c8fd00204e)
Supplement: Supplementary file 1 [file FD-217-C8FD00204E-s001.pdf]

Electronic Supplementary Material (ESI) for Faraday Discussions.
This journal is © The Royal Society of Chemistry 2018

Electronic Spectroscopy and Nanocalorimetry of Hydrated Magnesium Ions $[Mg(H_2O)_n]^+$, n = 20–70: Spontaneous Formation of a Hydrated Electron?

Thomas Taxer, Milan Ončák*, Erik Barwa, Christian van der Linde, Martin K. Beyer*

Institut für Ionenphysik und Angewandte Physik, Universität Innsbruck, Technikerstraße 25, 6020 Innsbruck, Austria

* Corresponding authors: milan.oncak@uibk.ac.at; martin.beyer@uibk.ac.at

Supporting Information

Contents

1. Calculation of the relative photodissociation cross sections 2
2. Additional computational data 4
3. Detailed figures with all dissociation channels 7
4. Control experiments on $[MgOH(H_2O)_n]^+$ 13
5. Mass spectra 15
6. Average binding energies 21
7. Correction of discontinuities in the spectra 36
8. Cartesian coordinates (in Å) and energies (in Hartree) of calculated structures 40

1. Calculation of the relative photodissociation cross sections

Photodissociation cross sections σ are extracted from measured ion intensities via eq. (S1).

$$I_0 = \sum_{i=0}^{n} I_i \, \mathrm{e}^{-\sigma \frac{\lambda p E}{h c A}} \quad \text{(S1)}$$

Here, I_0 is the parent ion intensity and I_i is the intensity of fragment ion *i*. The laser wavelength is represented by λ, the number of pulses by *p* and the pulse energy by *E*; *h* is Planck's constant, *c* is the speed of light and *A* represents the area covered by the laser beam at the position of the ICR cell.

Partial cross sections were calculated via eqs. (S2) and (S3).

$$\sigma = \sigma_{H2O} + \sigma_H \quad \text{(S2)}$$

$$\frac{\sigma_{H2O}}{\sigma_H} = \frac{I_{H2O}}{I_H} \quad \text{(S3)}$$

Here, σ_{H2O} is the partial cross section for the water evaporation channel (I) and σ_H is the partial cross section for the hydrogen dissociation channel (II). I_{H2O} is the sum of the intensities of all $[Mg(H_2O)_n]^+$ products created with a single photon and all other fragments created with two or more photons and I_H is the sum of the intensities of all $[MgOH(H_2O)_n]^+$ products created with a single photon.

The setup for the laser irradiation is shown in Figure S1.

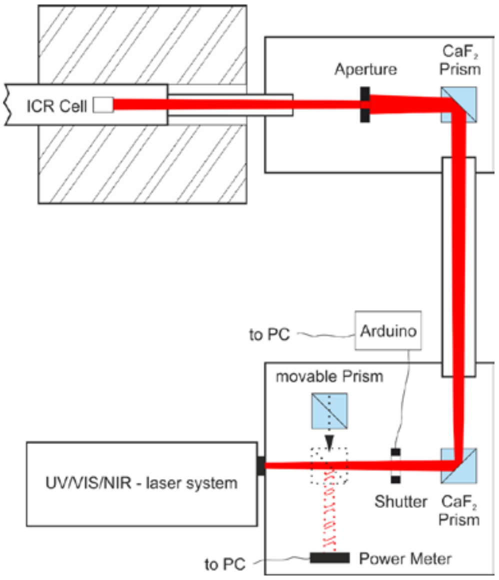

Figure S1: Optical setup for the experiments.

The number of pulses *p* is controlled by an electronically controlled shutter. The pulse energies *E* are measured by a power meter and absorptions of the CaF_2 prisms and the CaF_2 cell window are accounted for in the calculations. The wavelength λ is measured with a wavemeter. Figure S2 shows the power spectra of the laser system in the relevant photon energy range recorded during the experiments. The area *A* of the laser beam at the ICR cell is derived from the laser system manufacturer's specification of the beam diameter at the laser exit (5 mm, FWHM at 450 nm), the beam divergence (< 5 mrad, full angle at FWHM level at 450 nm) and the distance to the cell (4.6 m) in the experimental setup.

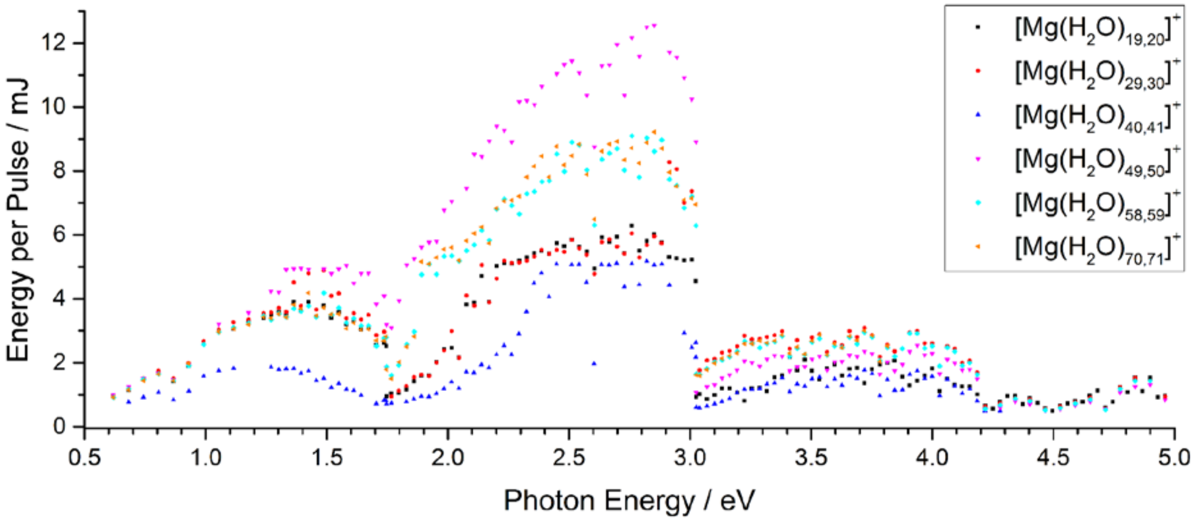

Figure S2: Pulse energies of the UV/VIS/IR - laser system as recorded during the experiments. Some optical components in the laser system were near the end of their lifetime, which caused the significant decrease in laser power, especially for $[Mg(H_2O)_{40,41}]^+$.

2. Additional computational data

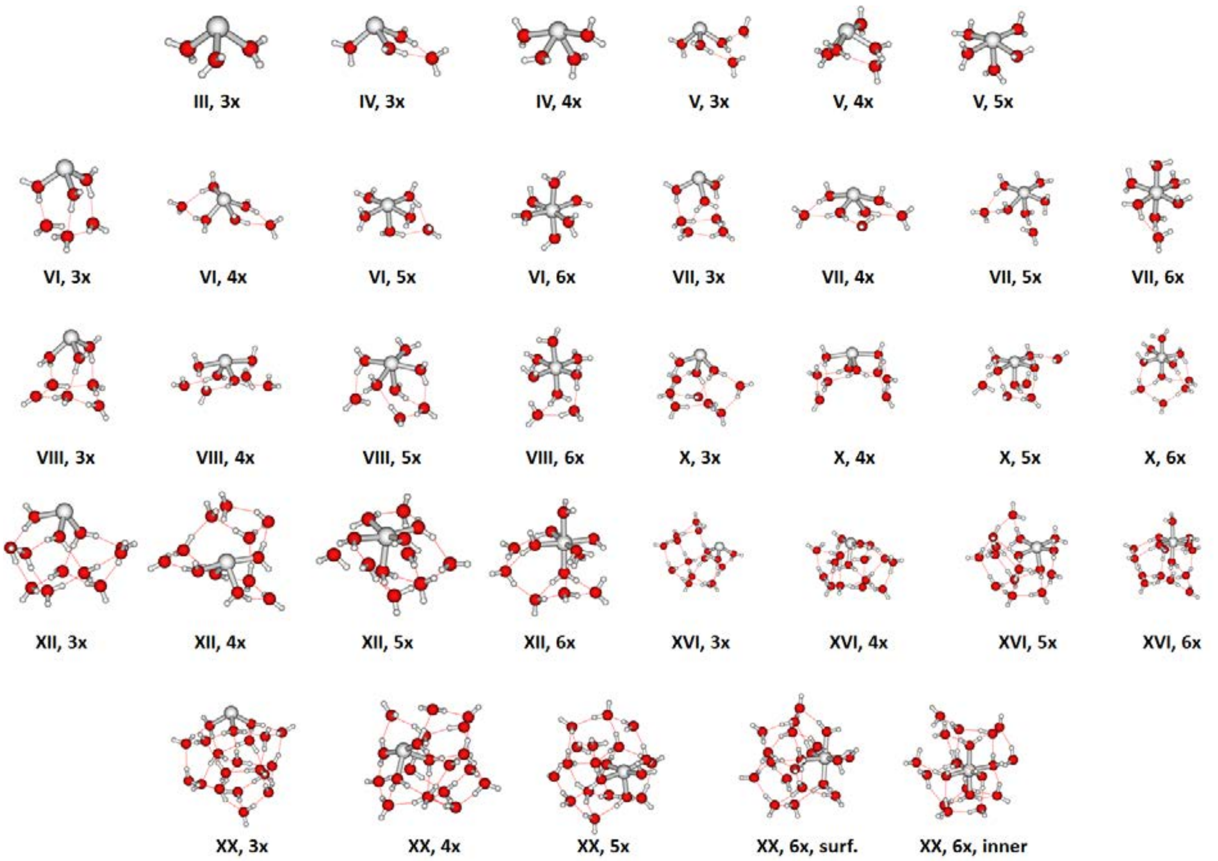

Figure S3: Structure of $[Mg(H_2O)_n]^+$ clusters optimized at the ωB97XD/def2TZVP level of theory with different coordination number of the Mg ion (3x–6x).

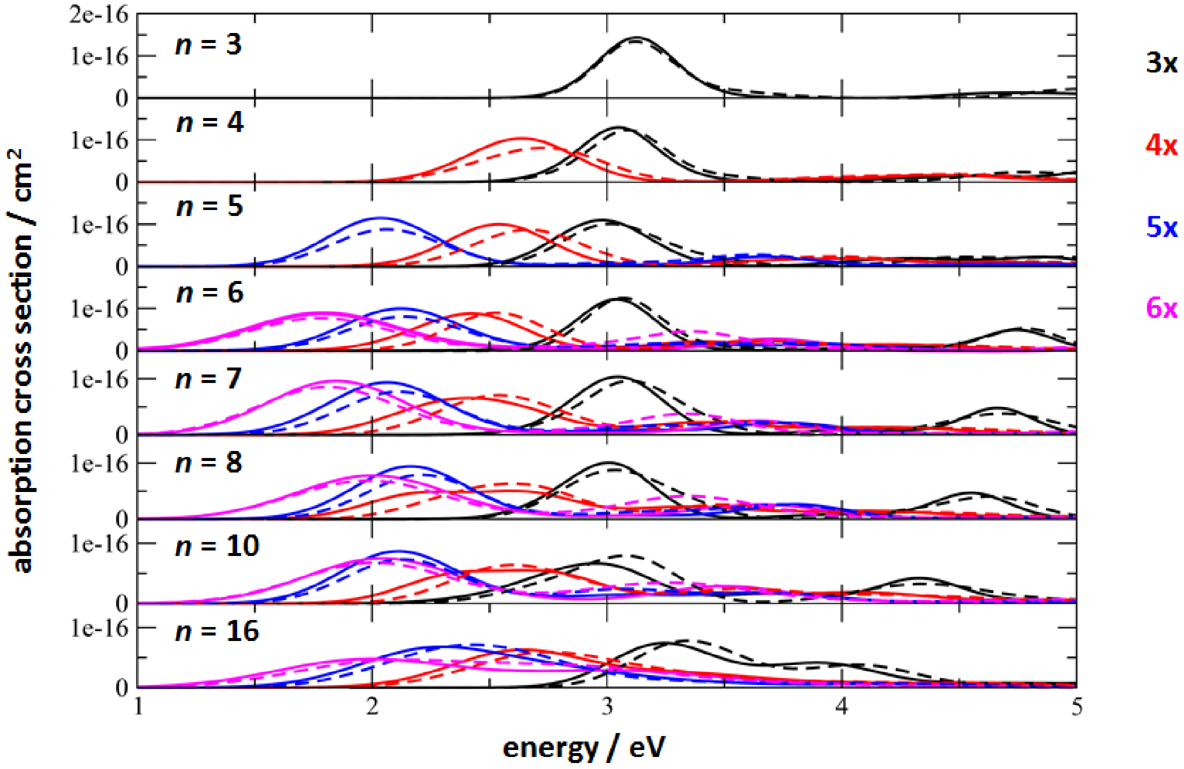

Figure S4: Comparison of $[Mg(H_2O)_n]^+$ clusters spectra calculated at the CAM-B3LYP/aug-cc-pVDZ level (full lines) and ADC(2)/aug-cc-pVDZ level (dashed lines). Peak width was calculated using the linearized reflection principle at the CAM-B3LYP/aug-cc-pVDZ//ωB97XD/def2TZVP level of theory for both methods. 20 ADC(2) excited states were considered for 16(6x).

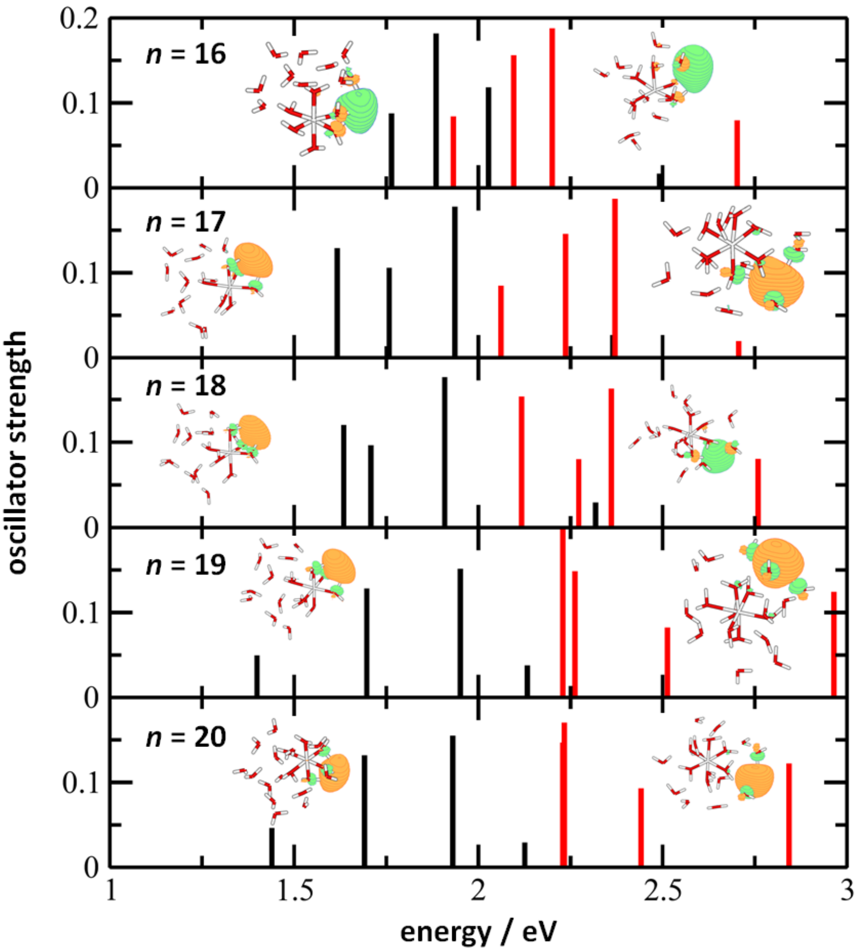

Figure S5: Lowest four electronic transitions in $[Mg(H_2O)_n]^+$ clusters, n = 16–20, for two isomers with the electron in close contact with the $[Mg(H_2O)_6]^+$ core (black) and separated by water molecules (red). Calculated at the CAM-B3LYP/aug-cc-pVDZ//ωB97XD/def2TZVP level. HOMO orbitals calculated at the CAM-B3LYP/aug-cc-pVDZ level are shown for each structure.

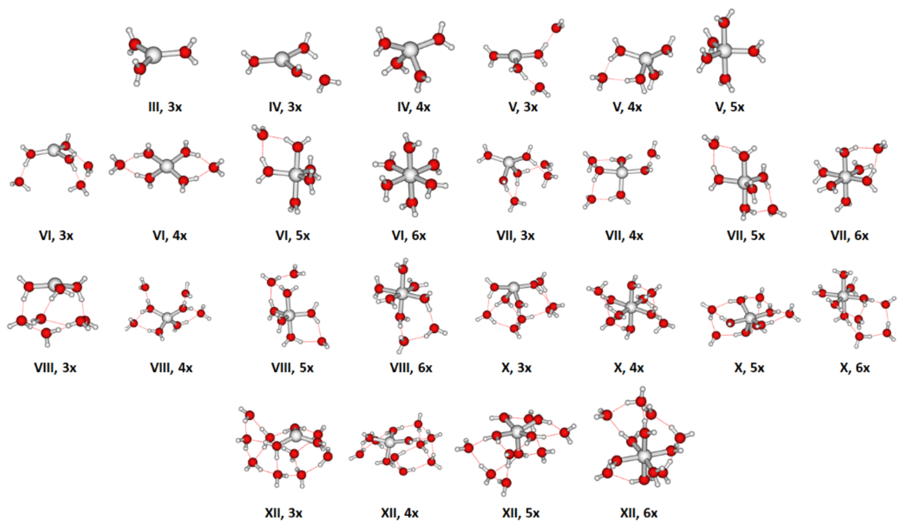

Figure S6: Structure of $[Mg(H_2O)_n]^+$ clusters optimized in the D_1 state at the ADC(2)/aug-cc-pVDZ level of theory.

3. Detailed figures with all dissociation channels

Figures S7–12 show the detailed photodissociation spectra of the investigated ions, along with their corresponding fragmentation pattern and experimental details for photon flux and number of laser pulses used.

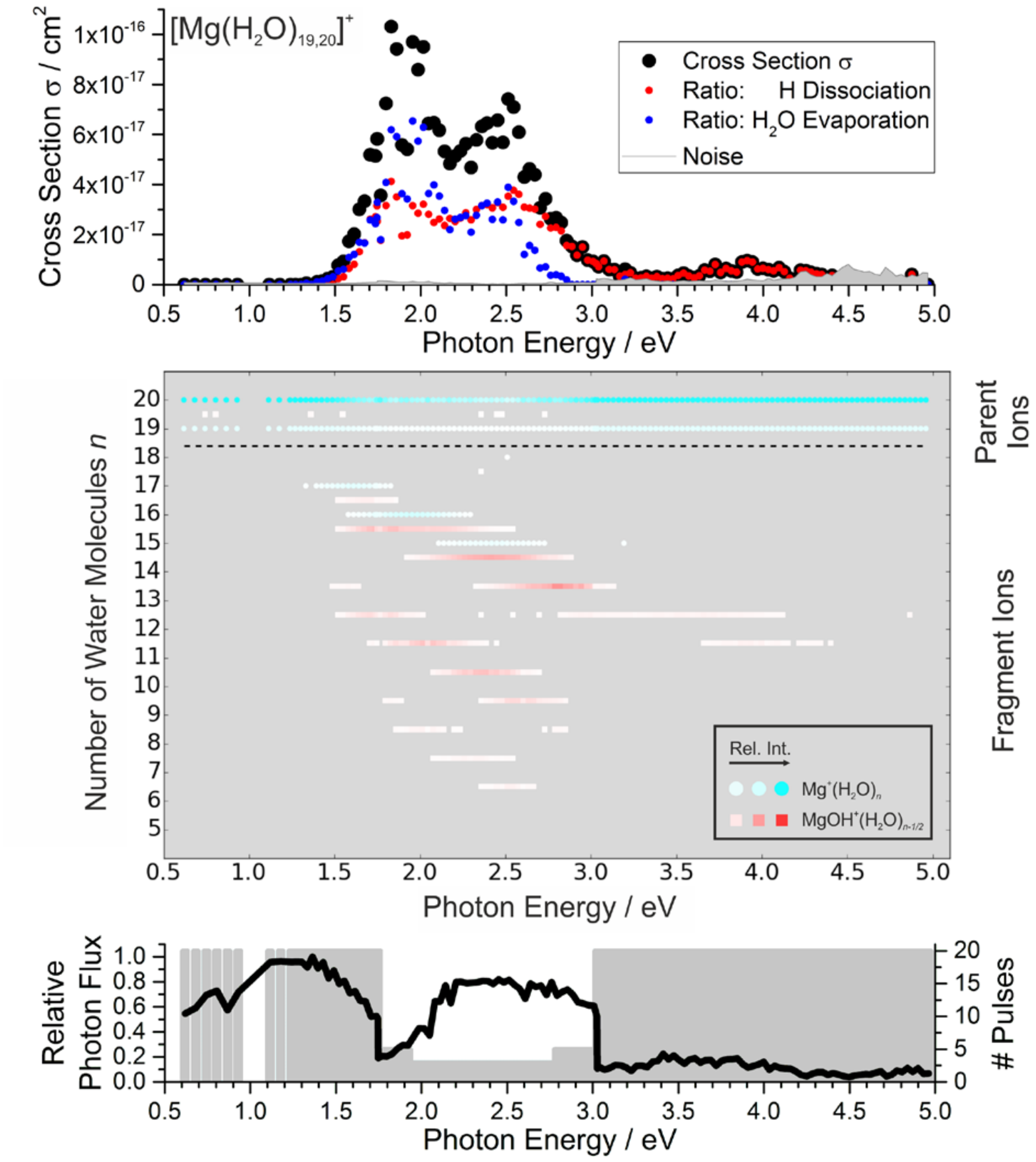

Figure S7: Detailed photodissociation spectrum of $[Mg(H_2O)_{19,20}]^+$, with corresponding fragmentation pattern and experimental details for photon flux and number of laser pulses used.

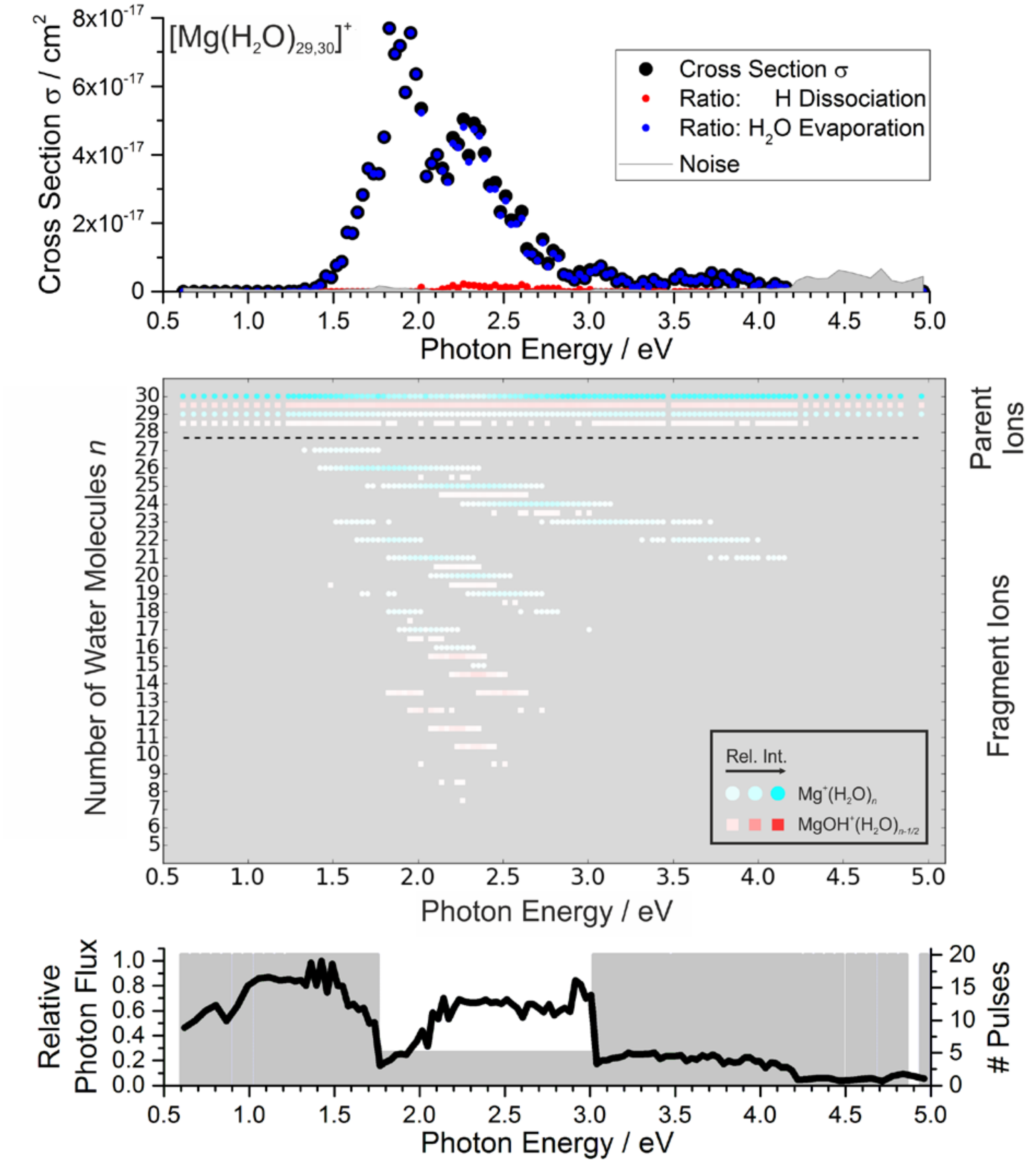

Figure S8: Detailed photodissociation spectrum of $[Mg(H_2O)_{29,30}]^+$, with corresponding fragmentation pattern and experimental details for photon flux and number of laser pulses used.

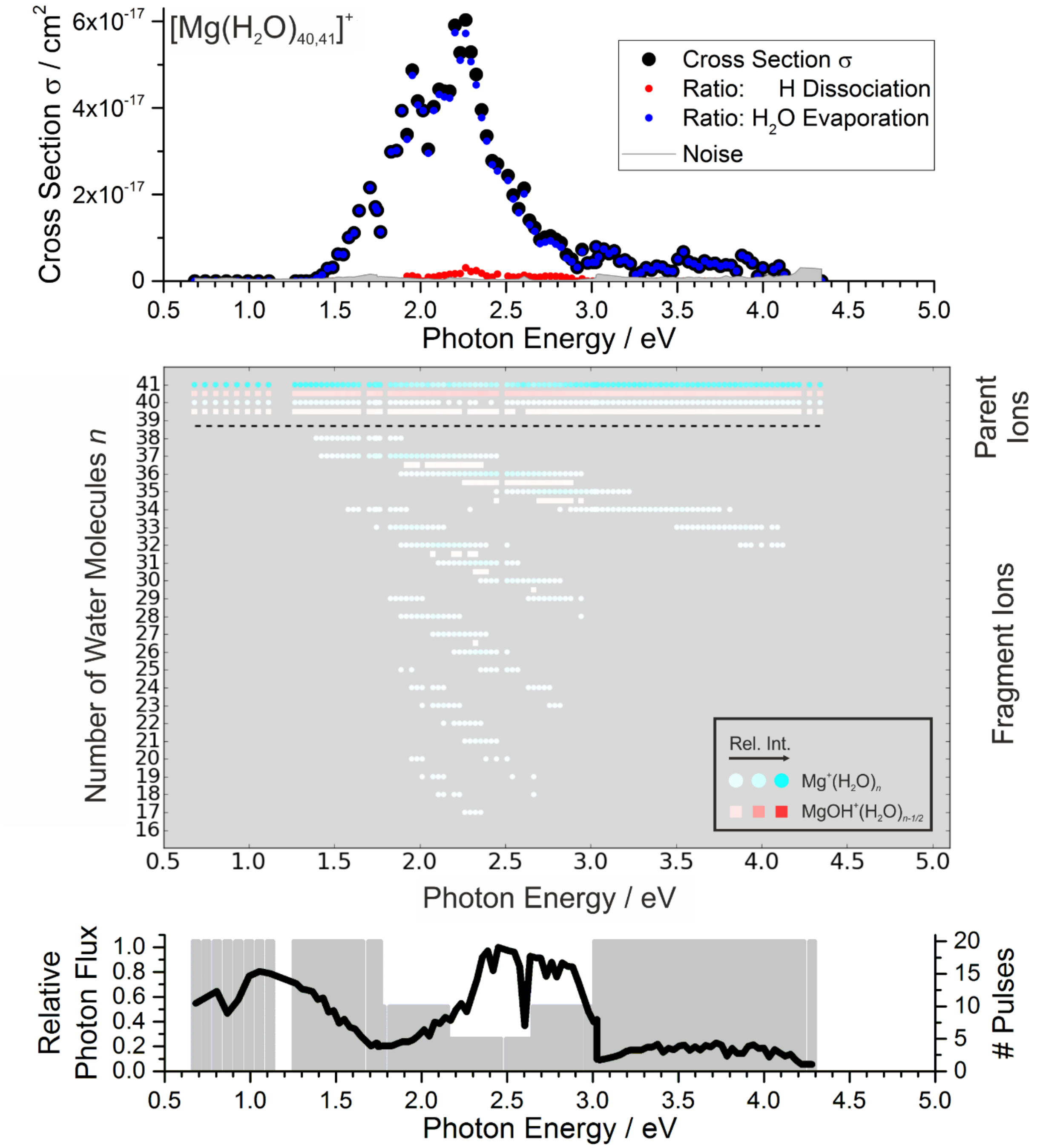

Figure S9: Detailed photodissociation spectrum of $[Mg(H_2O)_{40,41}]^+$, with corresponding fragmentation pattern and experimental details for photon flux and number of laser pulses used. Spectrum stopped at 4.35 eV due to significant decrease in laser power output.

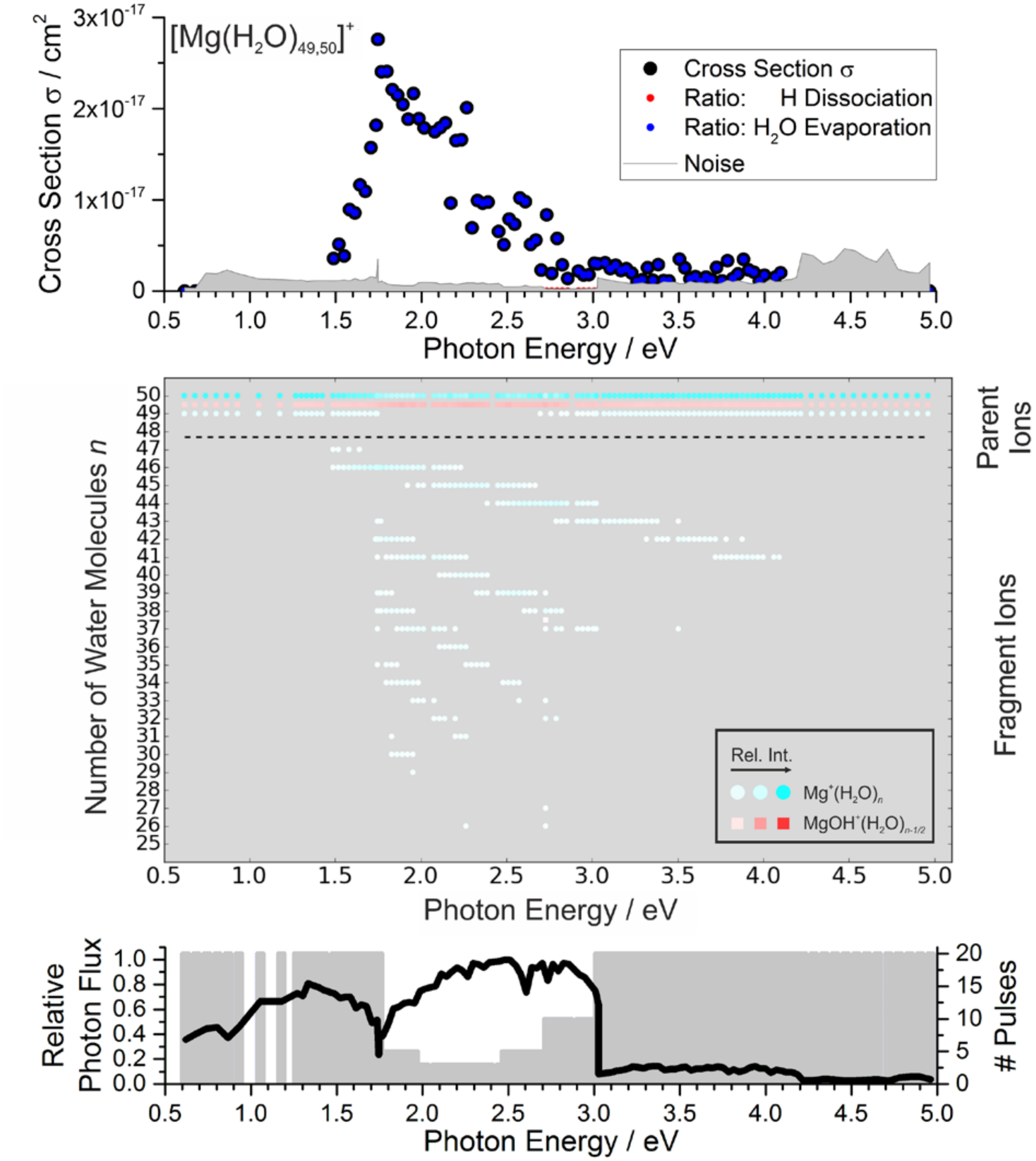

Figure S10: Detailed photodissociation spectrum of $[Mg(H_2O)_{49,50}]^+$, with corresponding fragmentation pattern and experimental details for photon flux and number of laser pulses used.

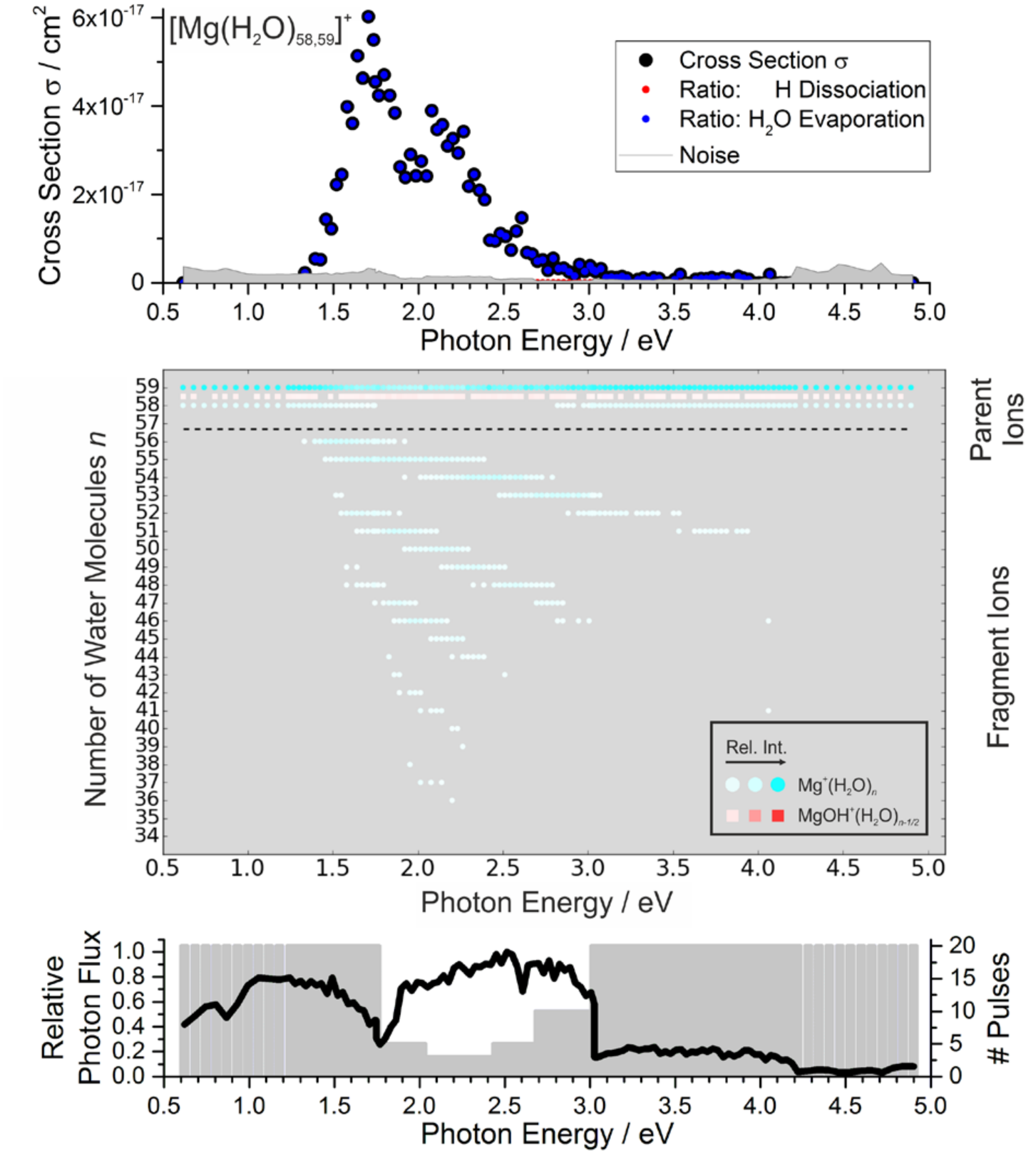

Figure S11: Detailed photodissociation spectrum of $[Mg(H_2O)_{58,59}]^+$, with corresponding fragmentation pattern and experimental details for photon flux and number of laser pulses used.

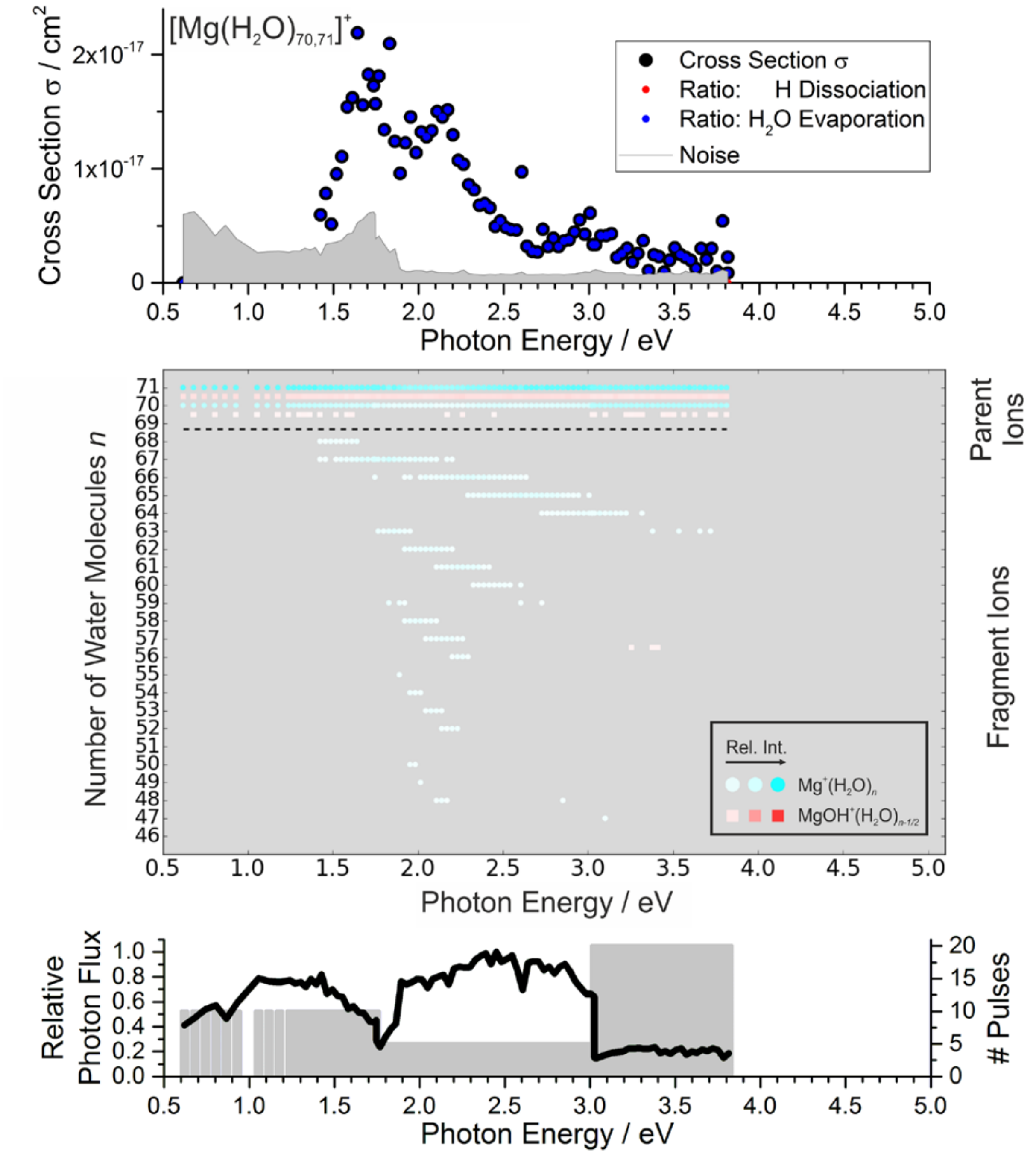

Figure S12: Detailed photodissociation spectrum of $[Mg(H_2O)_{70,71}]^+$, with corresponding fragmentation pattern and experimental details for photon flux and number of laser pulses used. Spectrum stopped at 3.8 eV due to signal decrease.

4. Control experiments on $[MgOH(H_2O)_n]^+$

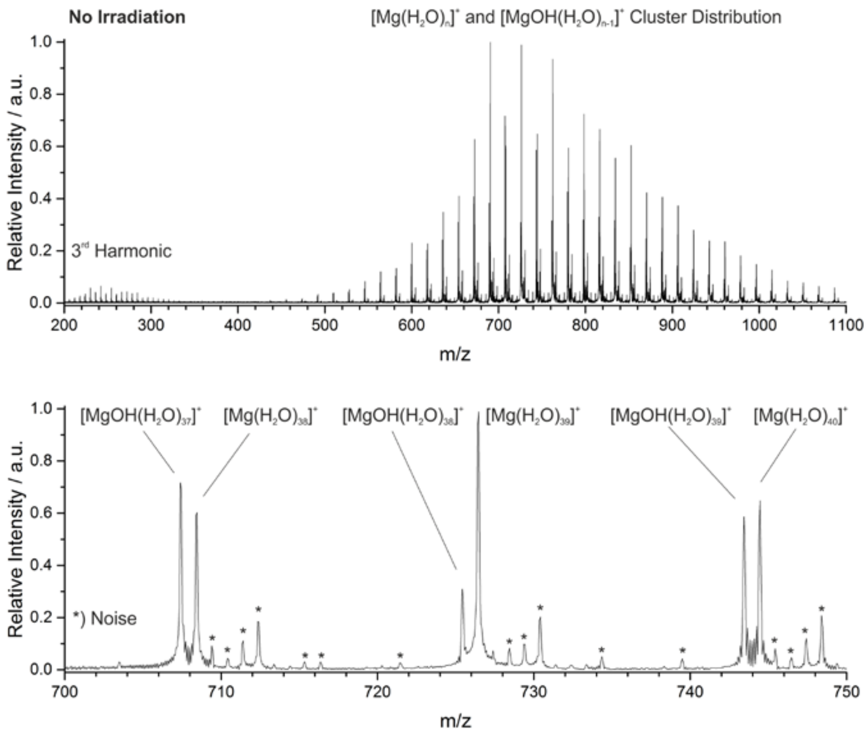

Figure S13: Mass spectrum of initial cluster distribution of $[Mg(H_2O)_n]^+$ and $[MgOH(H_2O)_{n-1}]^+$ ions.

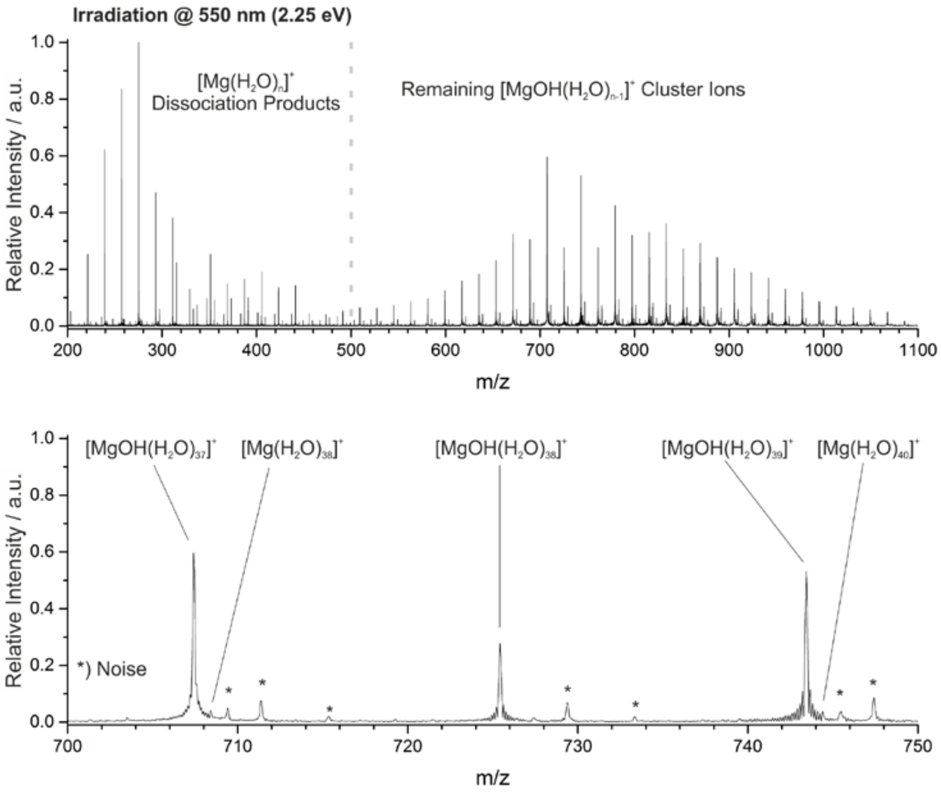

Figure S14: Mass spectrum after irradiating initial $[Mg(H_2O)_n]^+$ and $[MgOH(H_2O)_{n-1}]^+$ cluster distribution at 550 nm (2.25 eV) with 200 laser pulses (for 10 s). This shows that $[MgOH(H_2O)_{n-1}]^+$ do not absorb.

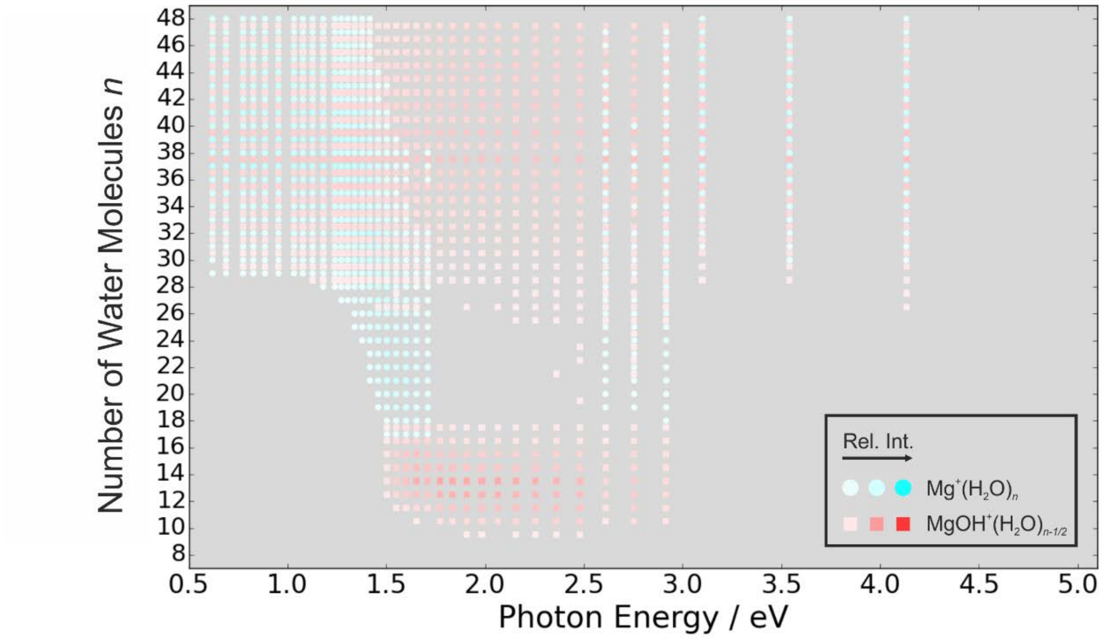

Figure S15: Fragmentation pattern of an initial cluster distribution of $[Mg(H_2O)_n]^+$ and $[MgOH(H_2O)_{n-1}]^+$ ions (for $29 \leq n \leq 48$) in the relevant wavelength range. Once formed, $[MgOH(H_2O)_{n-1}]^+$ do no longer fragment, confirming that no absorption takes place in these closed-shell systems within the studied energy range.

5. Mass spectra

Typical mass spectra for the observed ions are shown in Figures S16 – S33 at selected photon energies, as well as without irradiation.

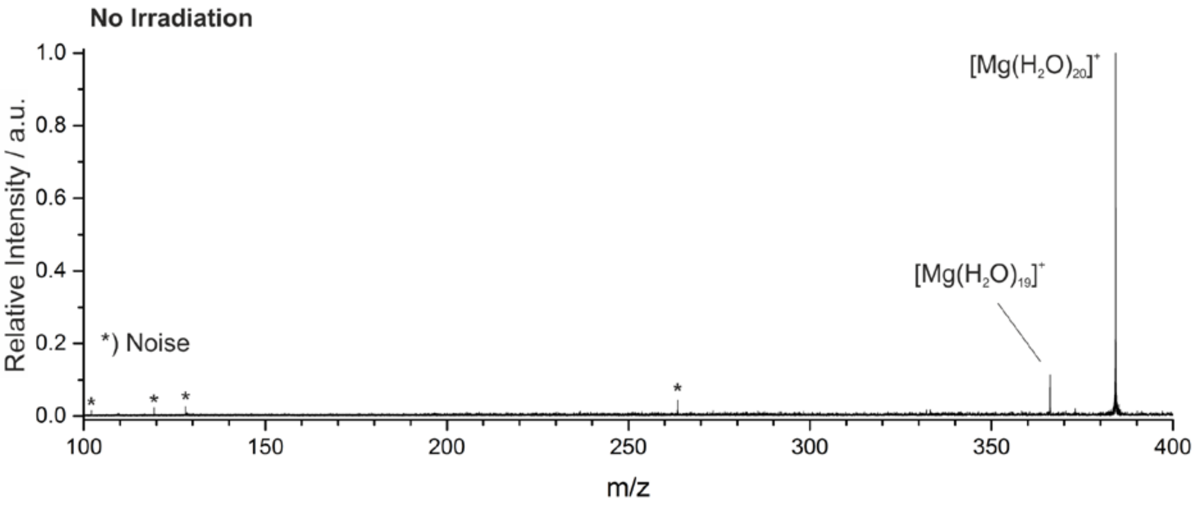

Figure S16: Mass spectrum of $[Mg(H_2O)_{19,20}]^+$ ions without laser irradiation.

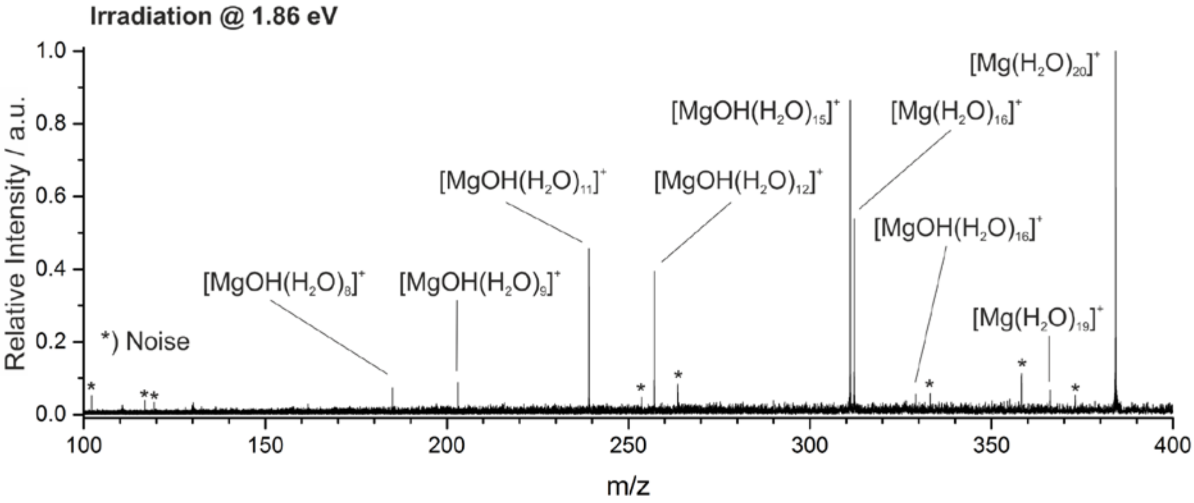

Figure S17: Mass spectrum of $[Mg(H_2O)_{19,20}]^+$ parent ions after laser irradiation at 1.86 eV.

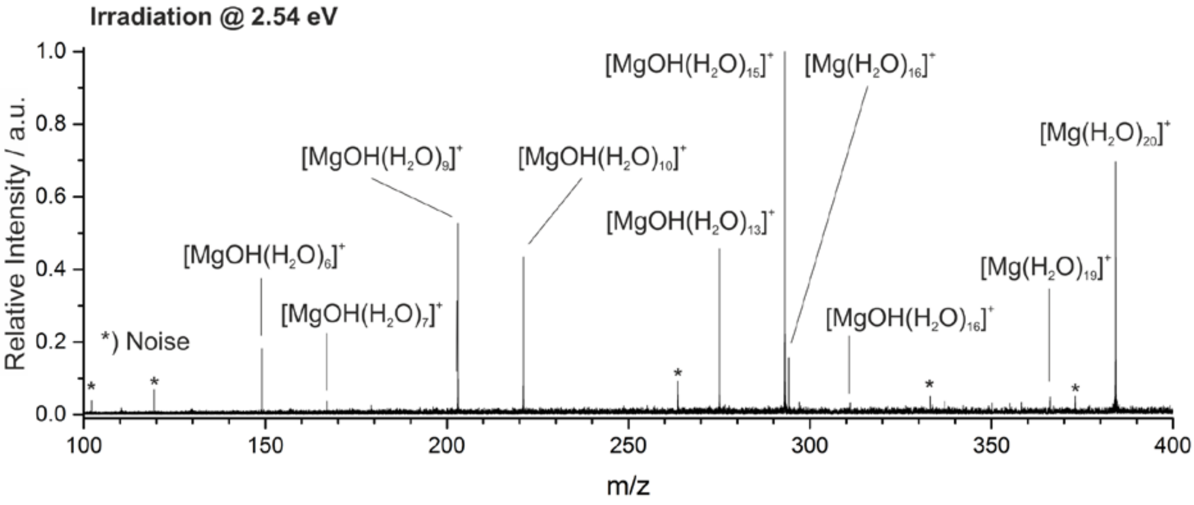

Figure S18: Mass spectrum of $[Mg(H_2O)_{19,20}]^+$ parent ions after laser irradiation at 2.54 eV.

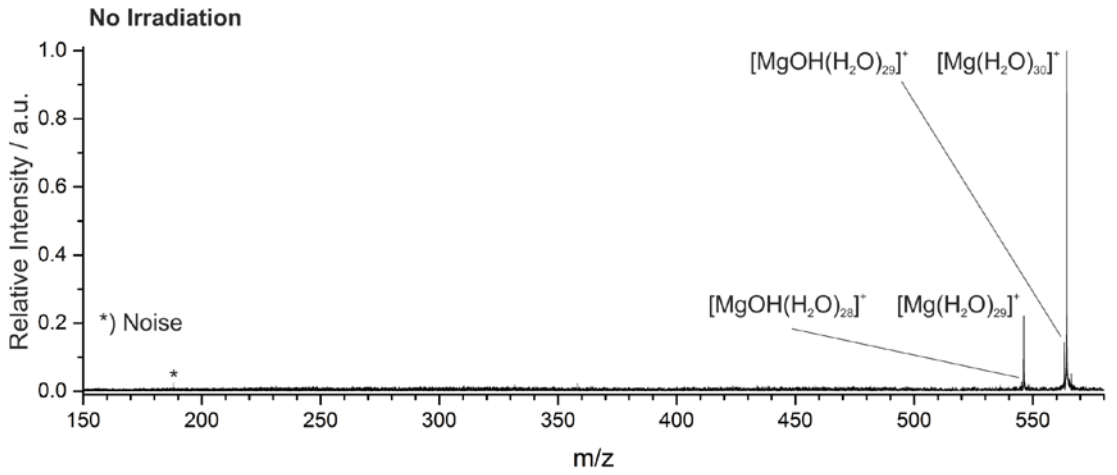

Figure S19: Mass spectrum of $[Mg(H_2O)_{29,30}]^+$ ions without laser irradiation.

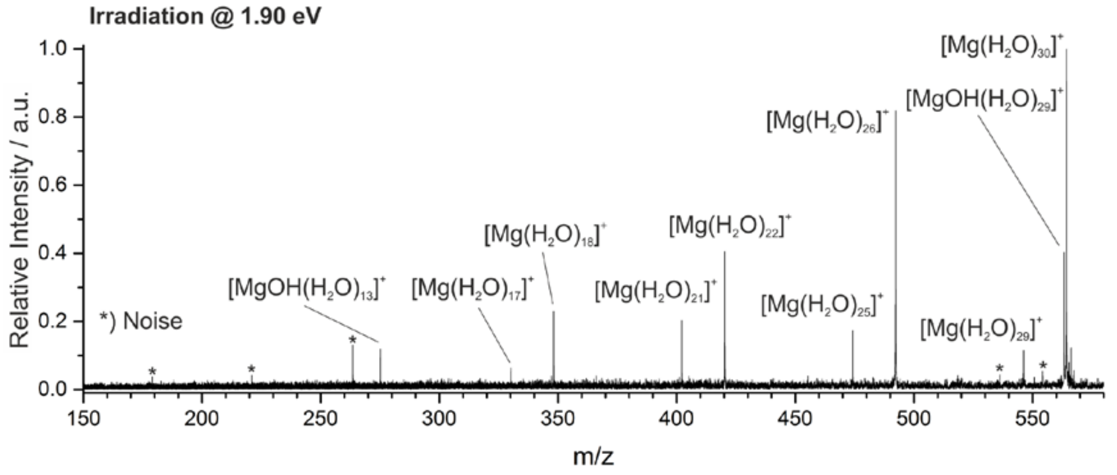

Figure S20: Mass spectrum of $[Mg(H_2O)_{29,30}]^+$ parent ions after laser irradiation at 1.90 eV.

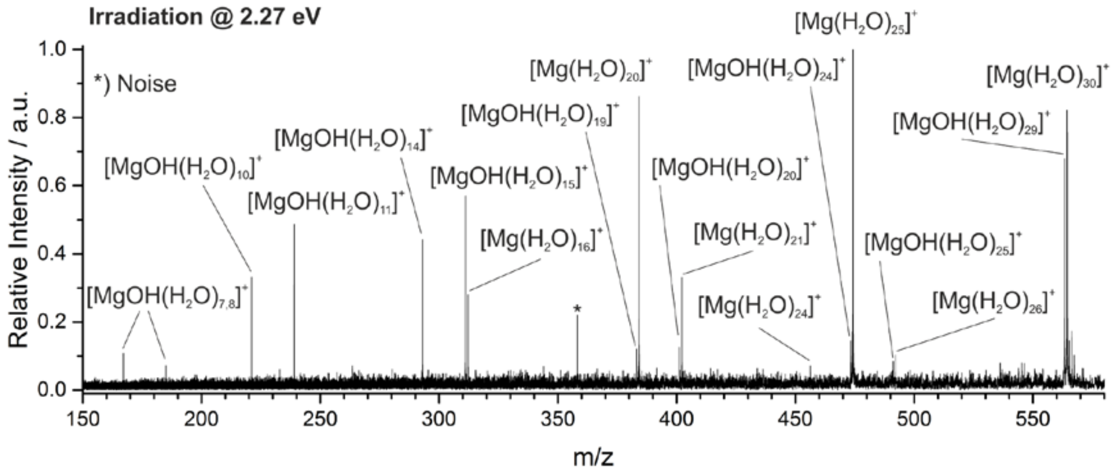

Figure S21: Mass spectrum of $[Mg(H_2O)_{29,30}]^+$ parent ions after laser irradiation at 2.27 eV.

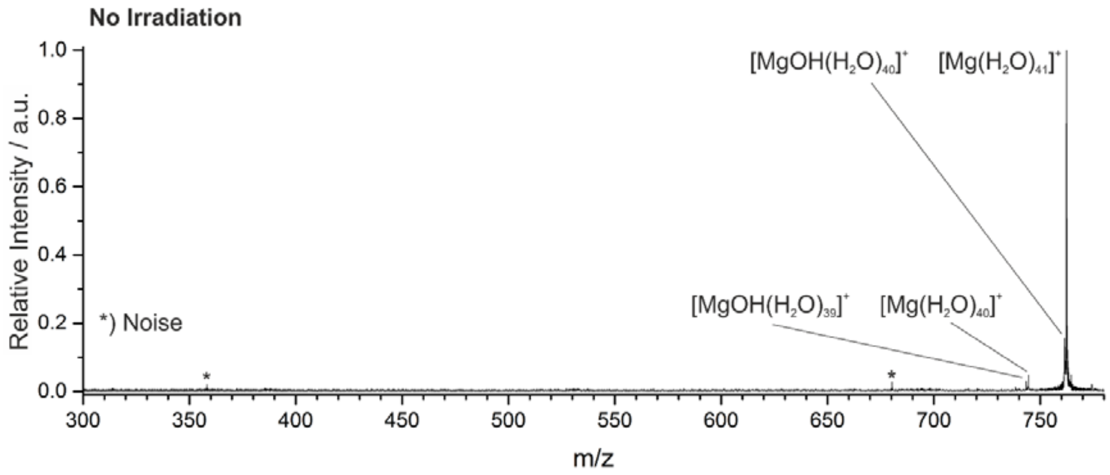

Figure S22: Mass spectrum of $[Mg(H_2O)_{40,41}]^+$ parent ions without laser irradiation.

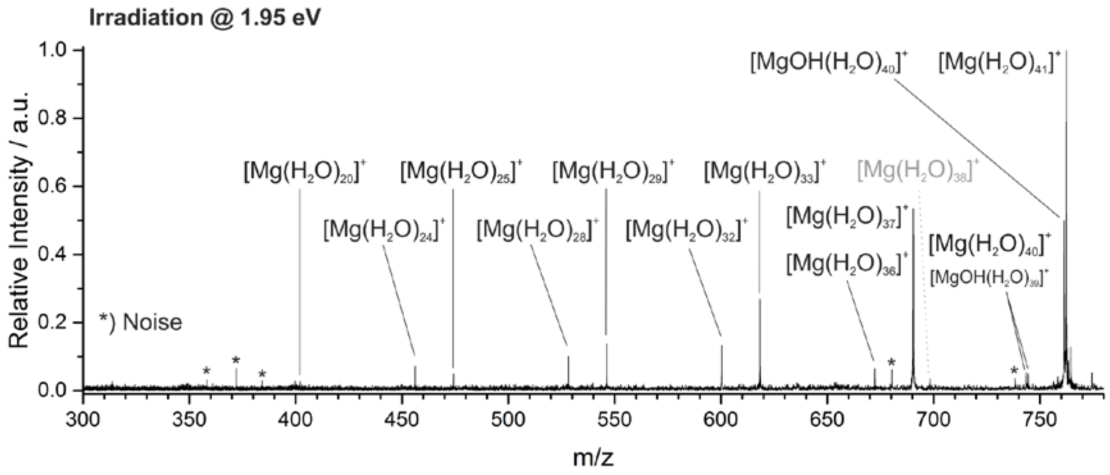

Figure S23: Mass spectrum of $[Mg(H_2O)_{40,41}]^+$ parent ions after laser irradiation at 1.95 eV. Fragments in gray are below the noise level.

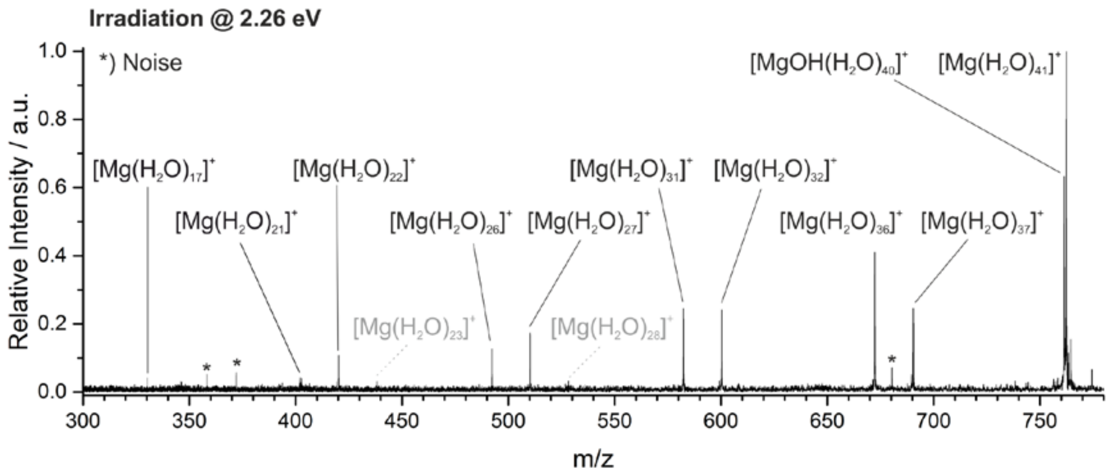

Figure S24: Mass spectrum of $[Mg(H_2O)_{40,41}]^+$ parent ions after laser irradiation at 2.26 eV. Fragments in gray are below the noise level.

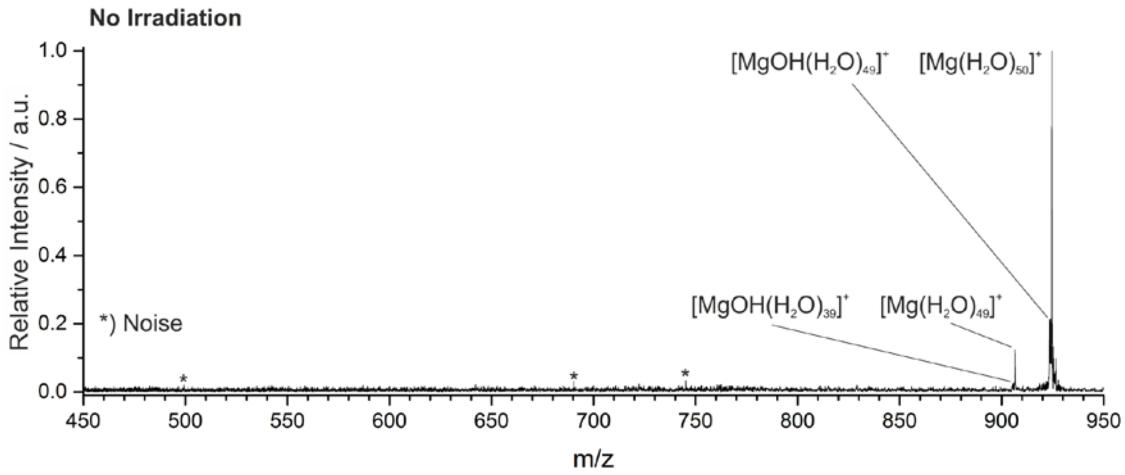

Figure S25: Mass spectrum of $[Mg(H_2O)_{49,50}]^+$ parent ions without laser irradiation.

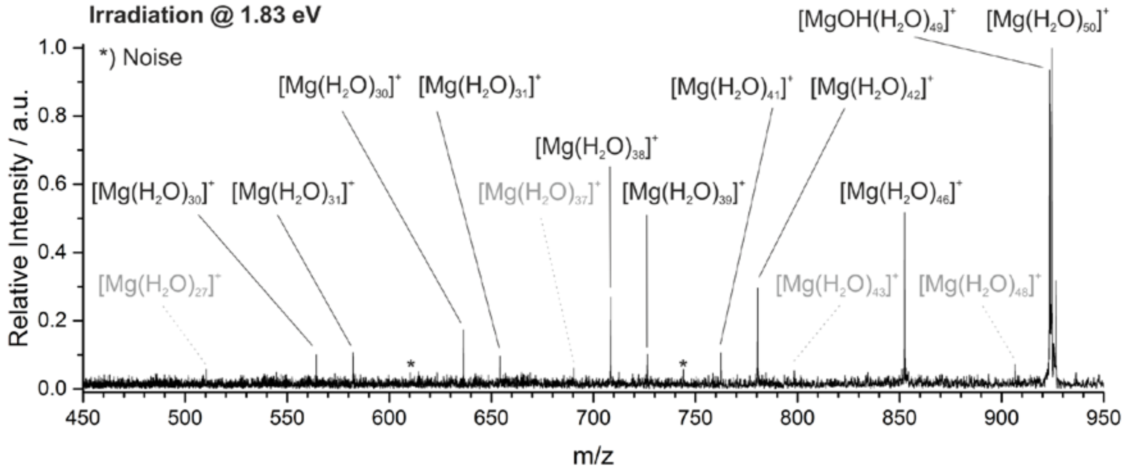

Figure S26: Mass spectrum of $[Mg(H_2O)_{49,50}]^+$ parent ions after laser irradiation at 1.83 eV. Fragments in gray are below the noise level.

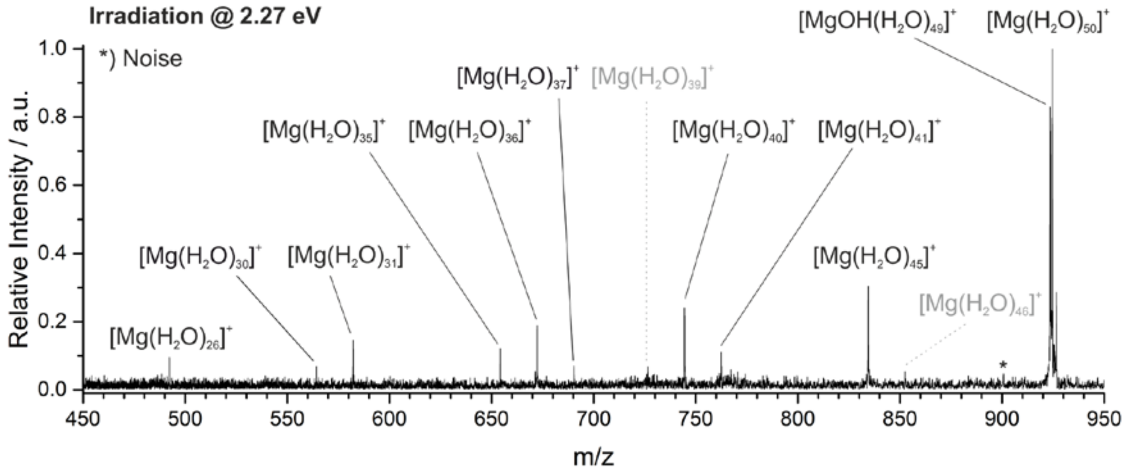

Figure S27: Mass spectrum of $[Mg(H_2O)_{49,50}]^+$ parent ions after laser irradiation at 2.27 eV. Fragments in gray are below the noise level.

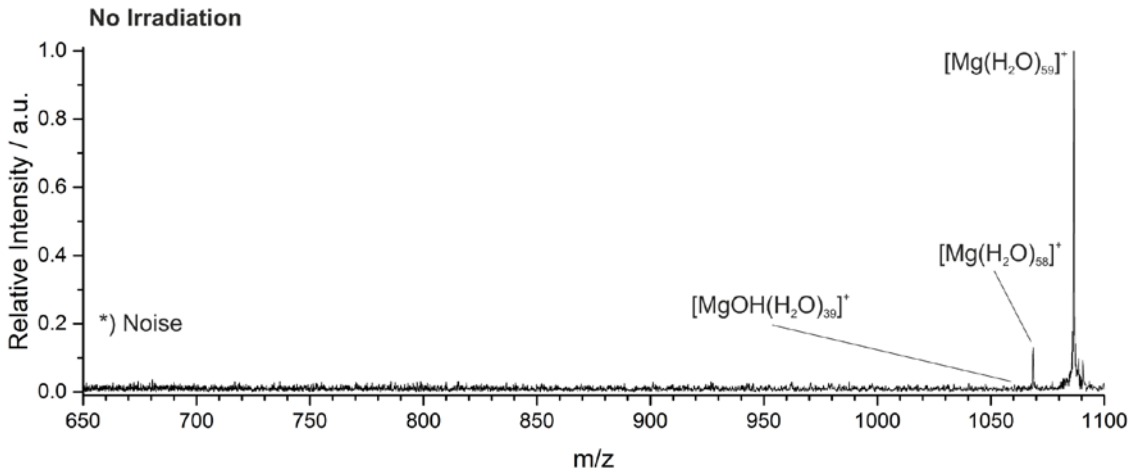

Figure S28: Mass spectrum of $[Mg(H_2O)_{58,59}]^+$ parent ions without laser irradiation.

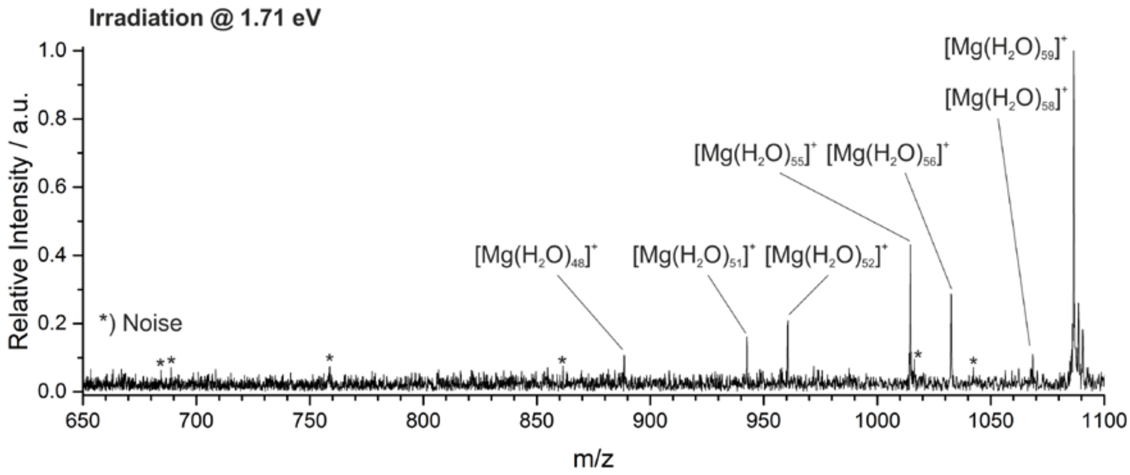

Figure S29: Mass spectrum of $[Mg(H_2O)_{58,59}]^+$ parent ions after laser irradiation at 1.71 eV.

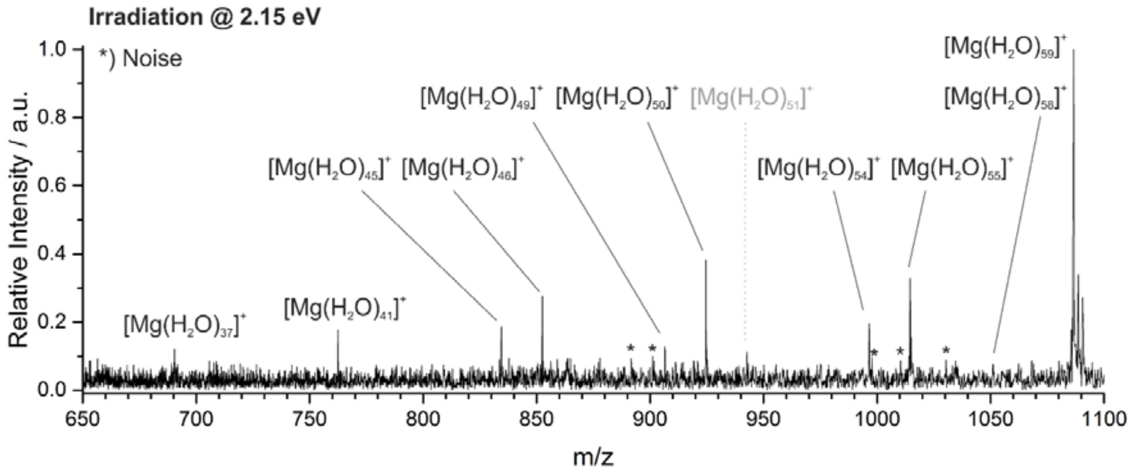

Figure S30: Mass spectrum of $[Mg(H_2O)_{58,59}]^+$ parent ions after laser irradiation at 2.15 eV. Fragments in gray are below the noise level.

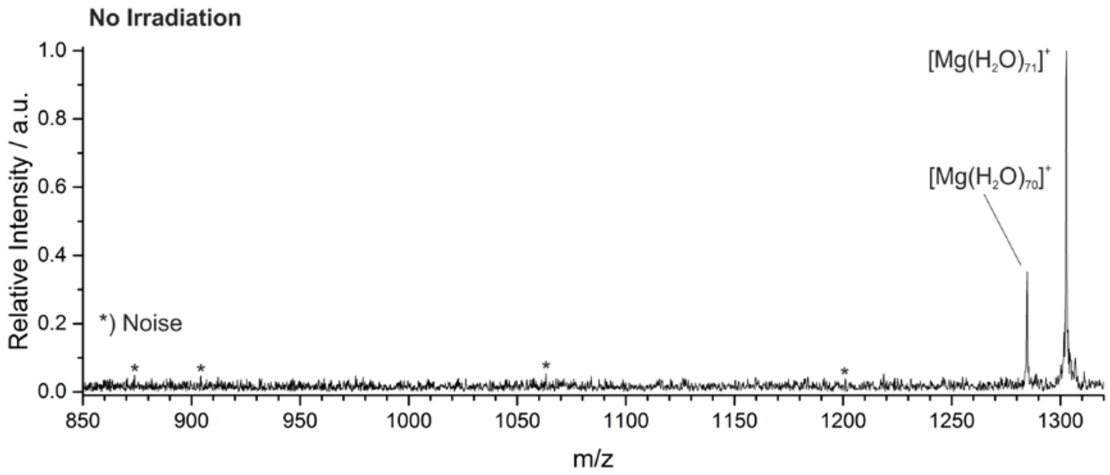

Figure S31: Mass spectrum of $[Mg(H_2O)_{70,71}]^+$ parent ions without laser irradiation.

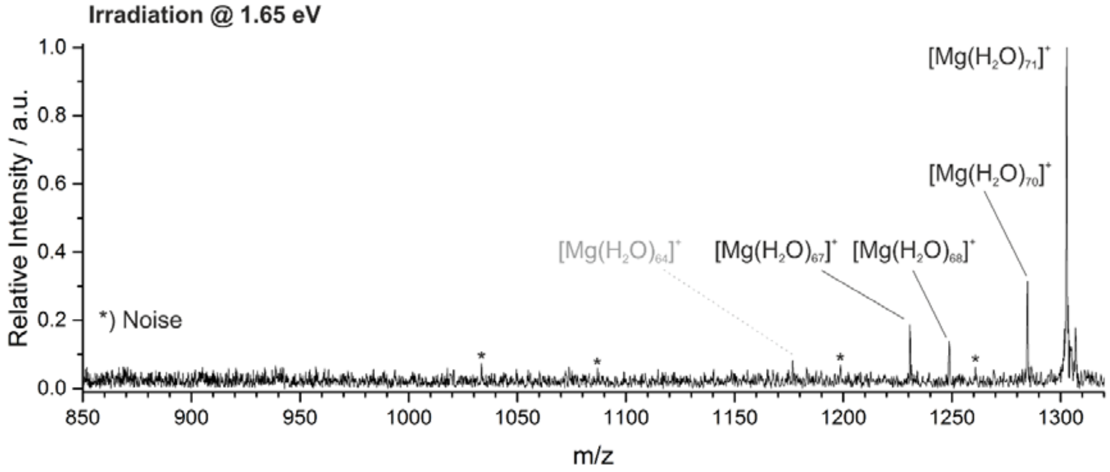

Figure S32: Mass spectrum of $[Mg(H_2O)_{70,71}]^+$ parent ions after laser irradiation at 1.65 eV. Fragments in gray are below the noise level.

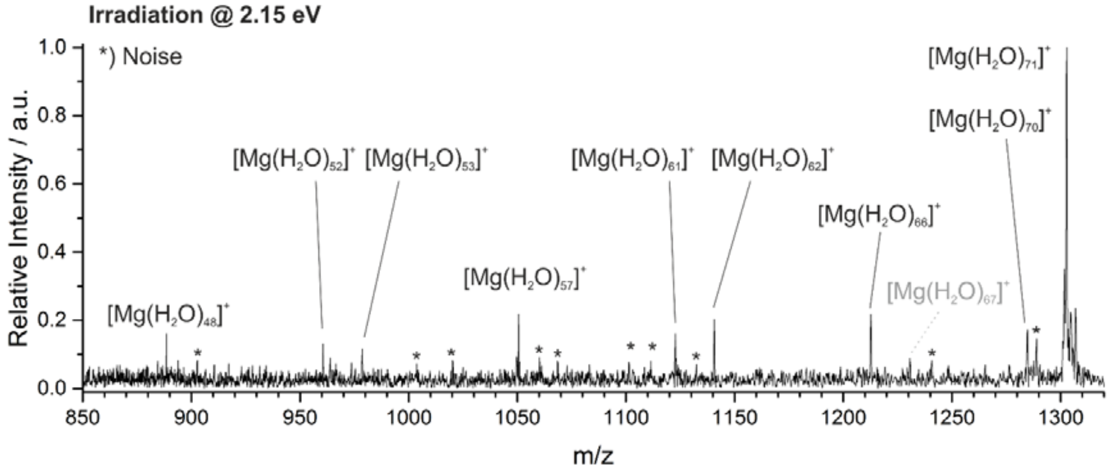

Figure S33: Mass spectrum of $[Mg(H_2O)_{70,71}]^+$ parent ions after laser irradiation at 2.15 eV. Fragments in gray are below the noise level.

6. Average binding energies

Table S1 – The table shows the average binding energy per water molecule *E / H₂O*. The energies were extracted from the photodissociation data using the equation $\langle E_{H_2O} \rangle = E_\gamma \gamma / m$. Here E_γ is the photon energy where the partial cross section for a specific fragmentation channel $[Mg(H_2O)_n]^+ \rightarrow [Mg(H_2O)_{n-m}]^+ + (H_2O)_m$ is at maximum (see SI for details). This energy is then multiplied by the number of photons γ involved in the dissociation and divided by the number of lost water molecules *m*.

# γ	1						2					3					4				5				
**n**	**20**	**30**	**40**	**50**	**60**	**70**	**30**	**40**	**50**	**60**	**70**	**30**	**40**	**50**	**60**	**70**	**40**	**50**	**60**	**70**	**40**	**50**	**60**	**70**	
**m**	**Energy per H_2O / eV**																								**Ø**
**3**	0.56	0.54	0.54	0.53	0.54																				**0.54**
**4**	0.48	0.46	0.48	0.43	0.44	0.43																			**0.45**
**5**	0.48	0.44	0.48	0.45	0.47																				**0.46**
**6**		0.44	0.48	0.44	0.44	0.44																			**0.45**
**7**		0.45	0.47	0.45	0.45	0.44	0.49	0.51		0.48															**0.47**
**8**		0.46	0.47	0.45		0.44	0.47	0.48	0.45	0.46	0.44														**0.46**
**9**		0.44		0.44			0.45	0.48	0.45	0.47	0.46														**0.46**
**10**							0.46	0.46	0.45	0.46	0.45														**0.46**
**11**							0.45	0.46	0.45	0.46		0.49			0.46										**0.46**
**12**								0.46	0.45	0.46		0.47	0.48	0.46	0.46										**0.46**
**13**												0.46	0.48	0.47	0.47	0.46									**0.47**
**14**												0.48	0.48			0.46									**0.47**
**15**												0.47	0.47		0.46	0.45		0.47							**0.46**
**16**													0.47	0.49				0.47							**0.48**
**17**													0.47				0.47		0.46						**0.46**
**18**																	0.47	0.48		0.46					**0.47**
**19**																	0.47	0.48							**0.48**
**20**																	0.47					0.47			**0.47**
**21**																	0.47				0.47				**0.47**
**22**																					0.48		0.47		**0.47**
**23**																					0.47			0.47	**0.47**
**24**																					0.48				**0.48**
**Ø**	**0.51**	**0.46**	**0.49**	**0.45**	**0.47**	**0.43**	**0.46**	**0.48**	**0.45**	**0.46**	**0.45**	**0.47**	**0.47**	**0.47**	**0.46**	**0.46**	**0.47**	**0.47**	**0.46**	**0.46**	**0.48**	**0.47**	**0.47**	**0.47**	**0.47**

The fits to determine the binding energies in Table S1 are shown in Figures S34 – S50.

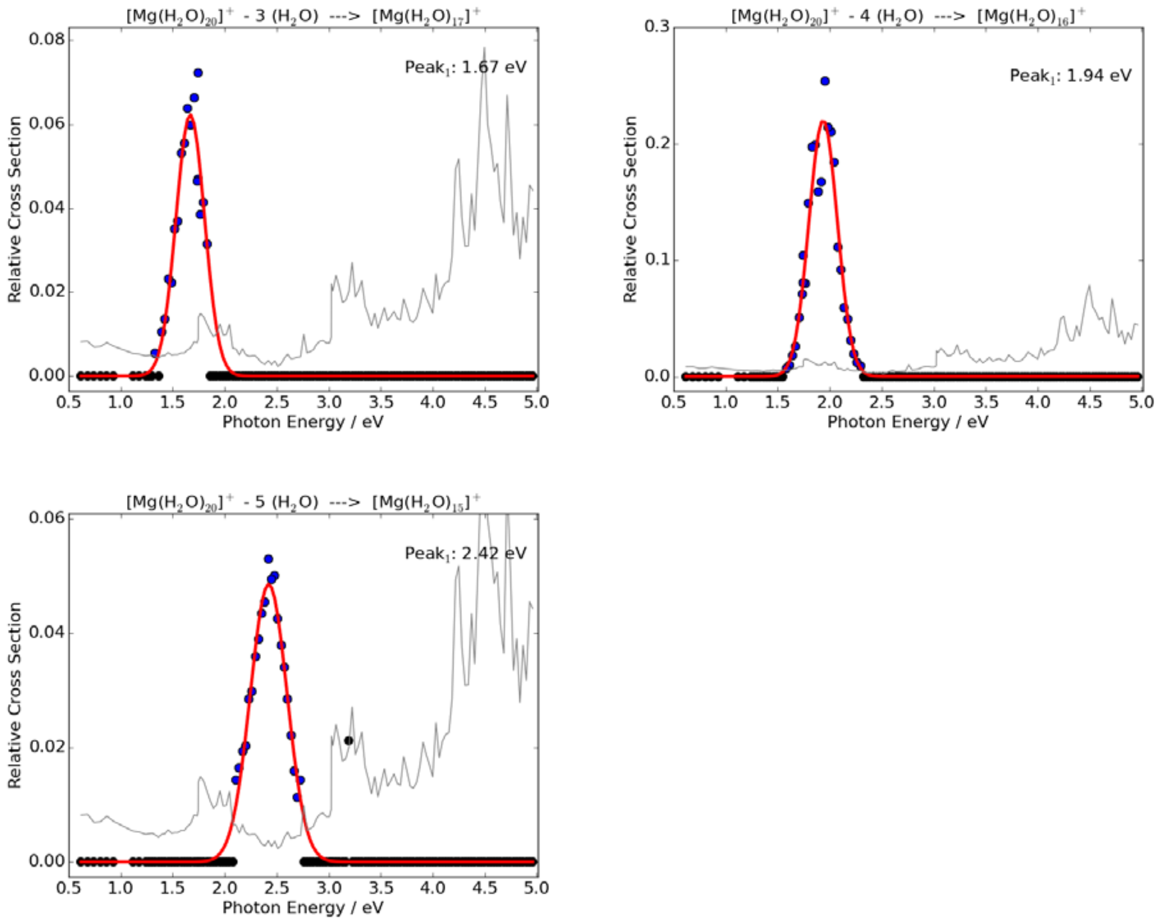

Figure S34: Fits to determine the centre of the fragment cross sections for the loss of 3, 4 and 5 water molecules for [Mg(H_2O)$_{20}$]$^+$. Blue dots: data points considered for fitting, black dots: not considered, red line: Gaussian fit, gray line: noise level.

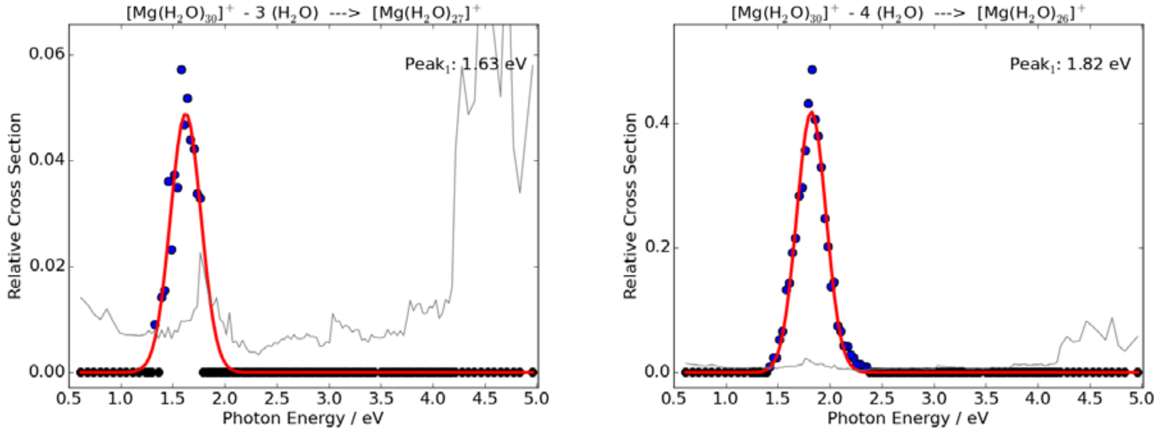

Figure S35: Fits to determine the centre of the fragment cross sections for the loss of 3 and 4 water molecules for [Mg(H_2O)$_{30}$]$^+$. Blue dots: data points considered for fitting, black dots: not considered, red line: Gaussian fit, gray line: noise level.

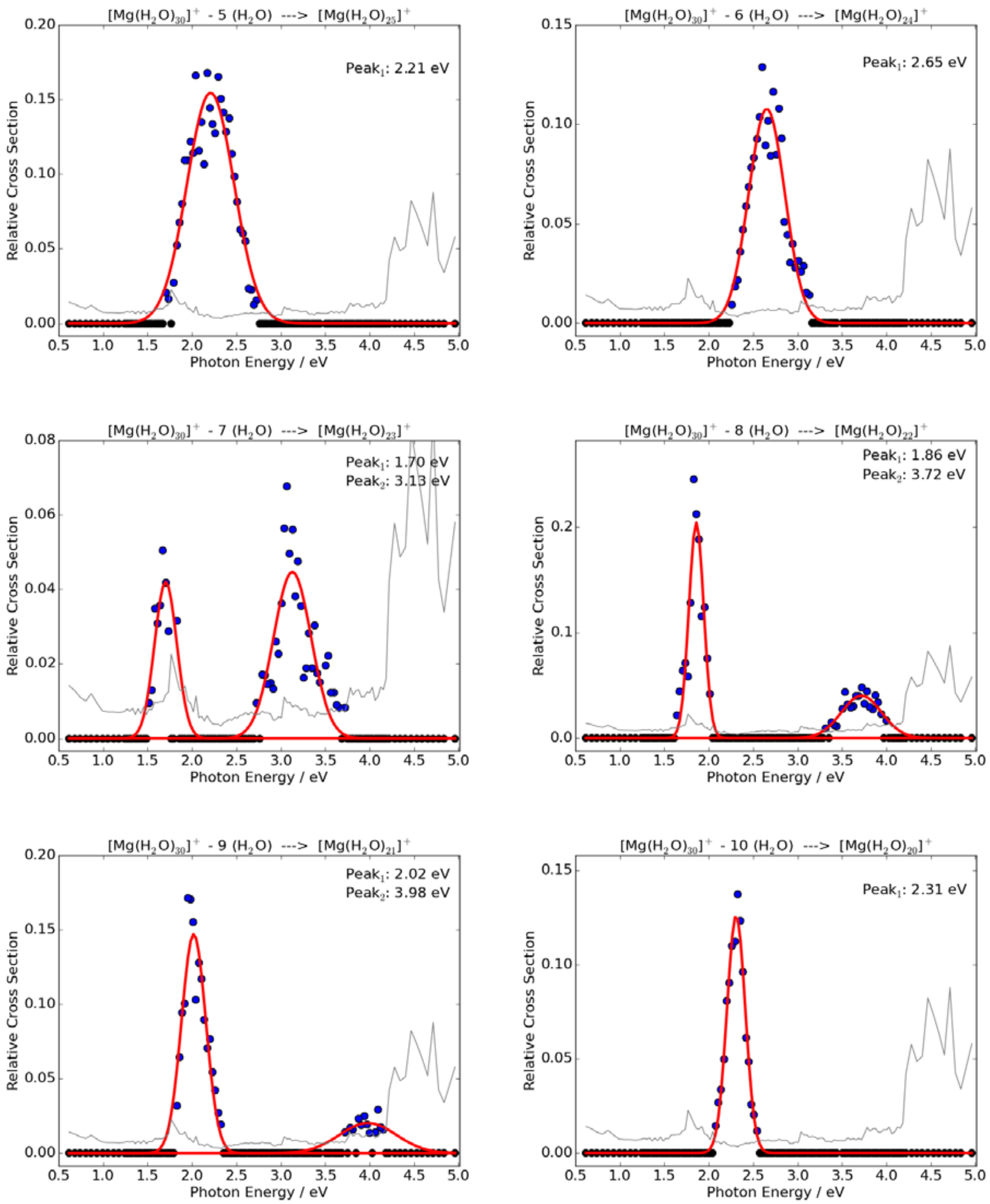

Figure S36: Fits to determine the centre of the fragment cross sections for the loss of 5, 6, 7, 8 , 9 and 10 water molecules for $[Mg(H_2O)_{30}]^+$. Blue dots: data points considered for fitting, black dots: not considered, red line: Gaussian fit, gray line: noise level.

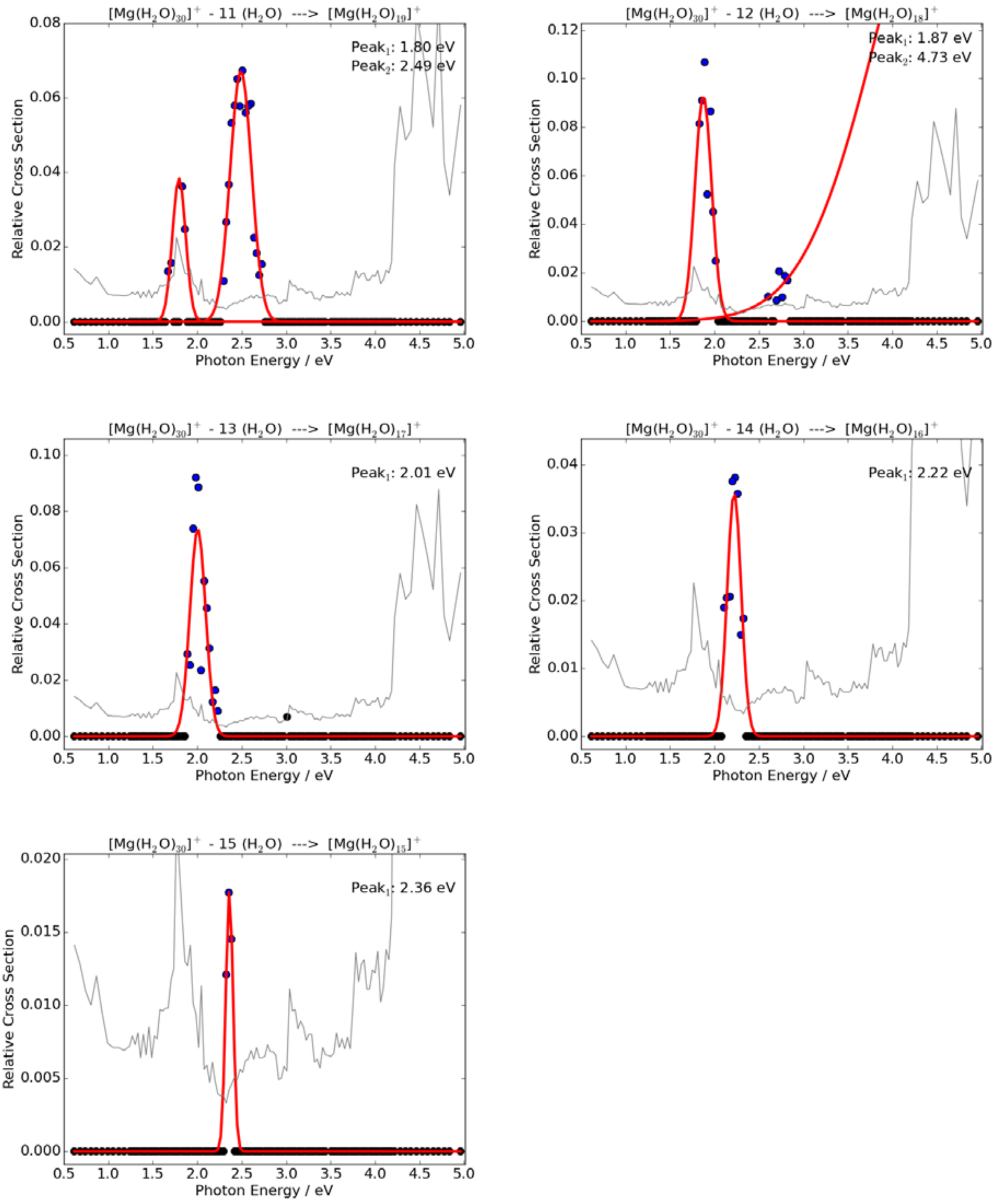

Figure S37: Fits to determine the centre of the fragment cross sections for the loss of 11, 12, 13, 14 and 15 water molecules for $[Mg(H_2O)_{30}]^+$. Blue dots: data points considered for fitting, black dots: not considered, red line: Gaussian fit, gray line: noise level.

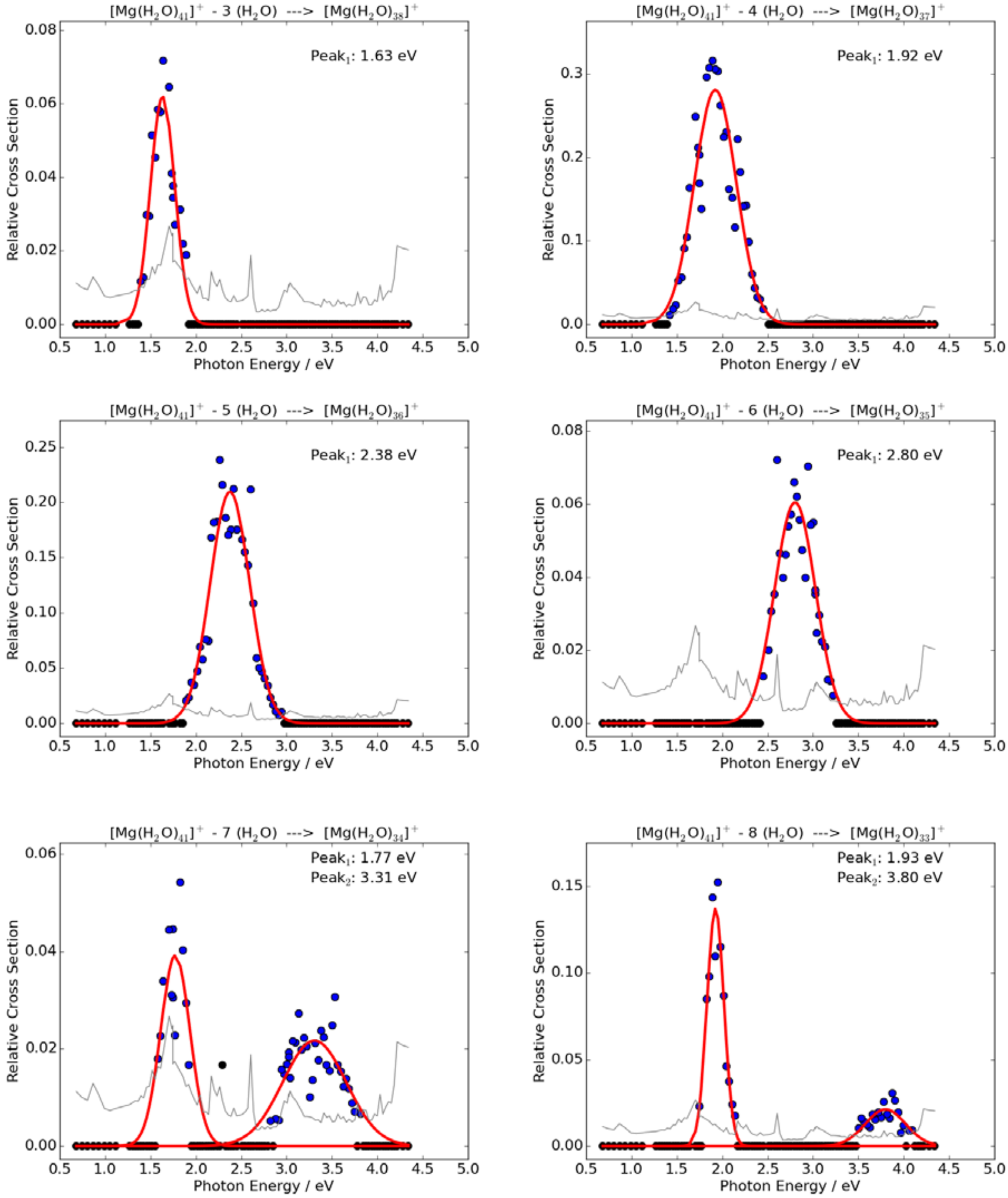

Figure S38: Fits to determine the centre of the fragment cross sections for the loss of 3, 4, 5, 6 , 7 and 8 water molecules for $[Mg(H_2O)_{41}]^+$. Blue dots: data points considered for fitting, black dots: not considered, red line: Gaussian fit, gray line: noise level.

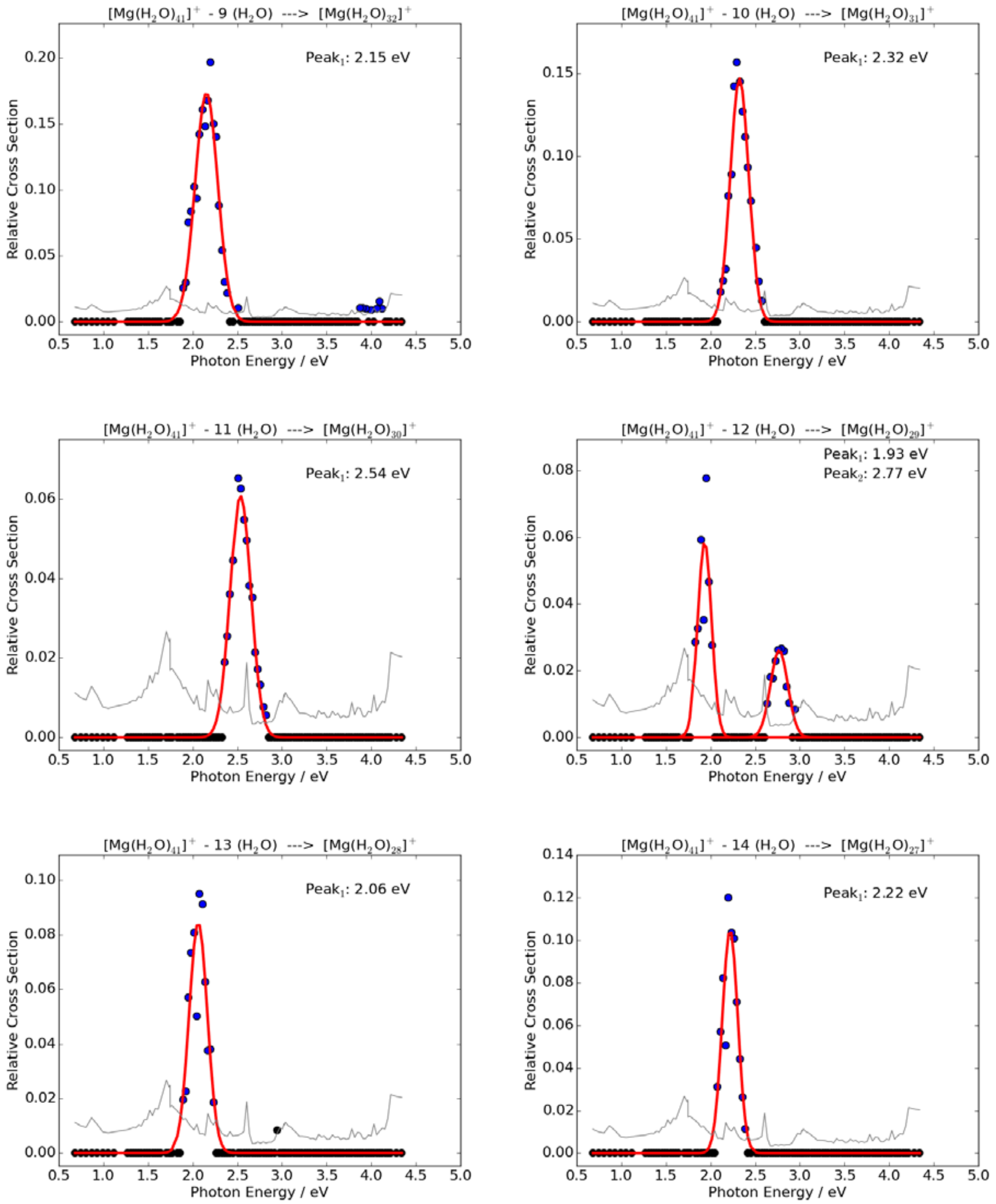

Figure S39: Fits to determine the centre of the fragment cross sections for the loss of 9, 10, 11, 12 , 13 and 14 water molecules for $[Mg(H_2O)_{41}]^+$. For the loss of 9 water molecules, the fit of the data points around 4.0 eV did not converge. Blue dots: data points considered for fitting, black dots: not considered, red line: Gaussian fit, gray line: noise level.

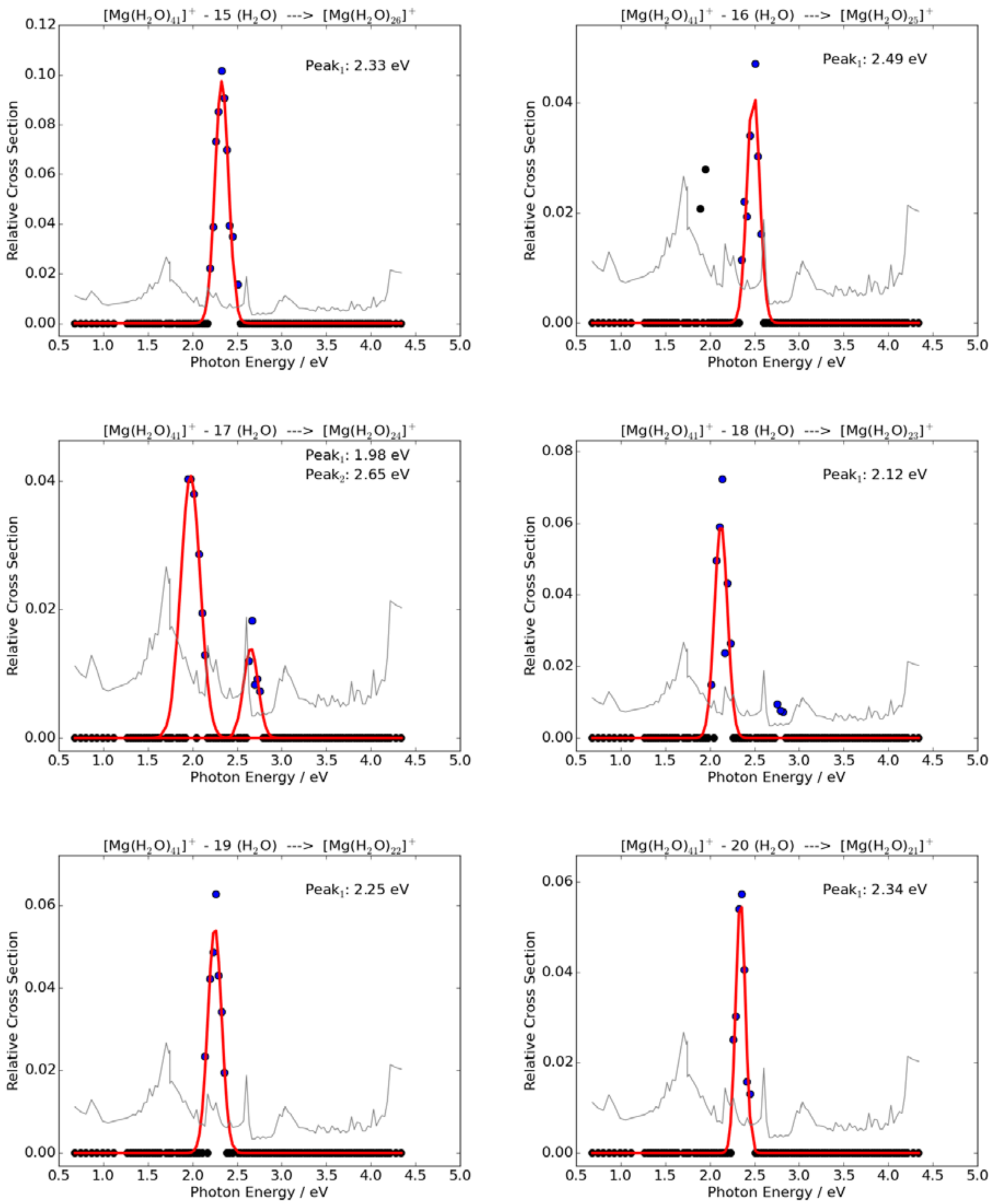

Figure S40: Fits to determine the centre of the fragment cross sections for the loss of 15, 16, 17, 18 , 19 and 20 water molecules for $[Mg(H_2O)_{41}]^+$. For the loss of 18 water molecules, the fit of the data points around 2.8 eV did not converge. Blue dots: data points considered for fitting, black dots: not considered, red line: Gaussian fit, gray line: noise level.

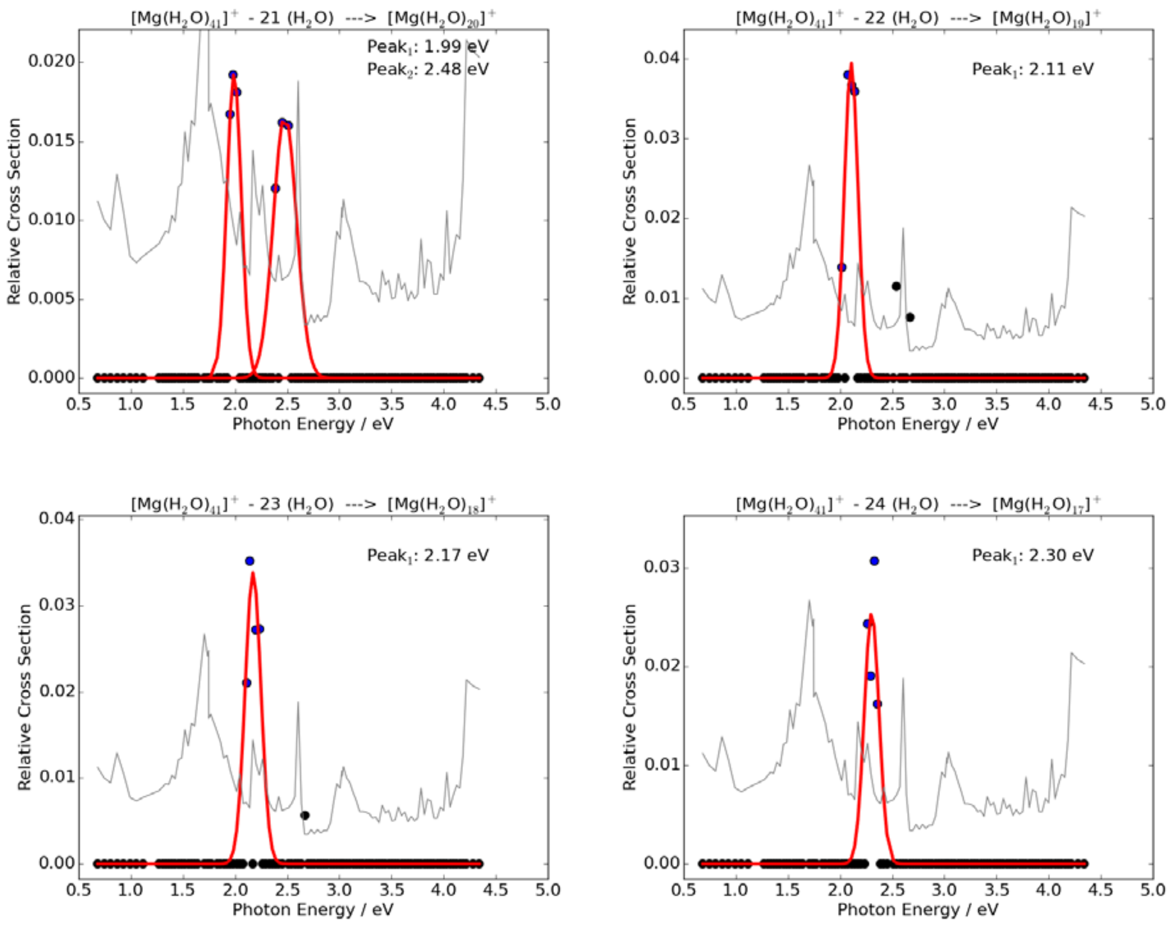

Figure S41: Fits to determine the centre of the fragment cross sections for the loss of 21, 22, 23 and 24 water molecules for $[Mg(H_2O)_{41}]^+$. Blue dots: data points considered for fitting, black dots: not considered, red line: Gaussian fit, gray line: noise level.

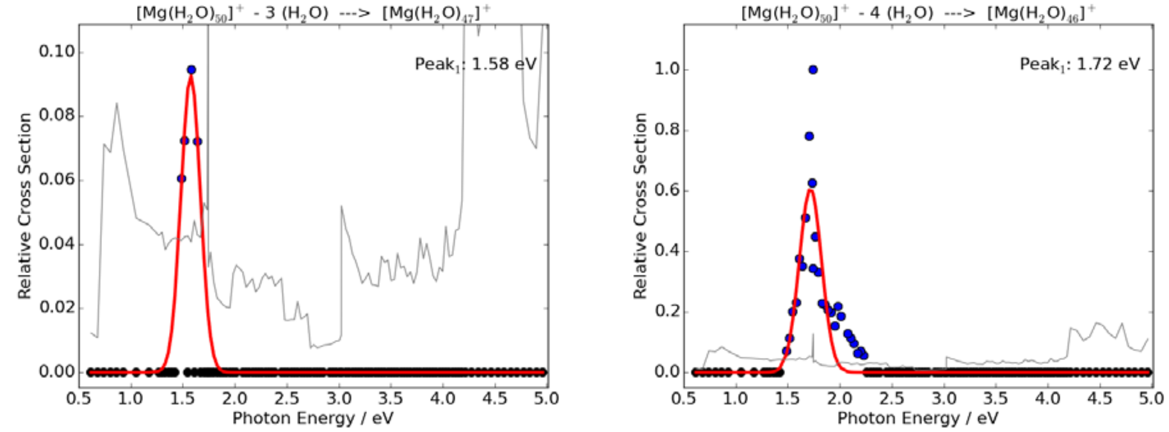

Figure S42: Fits to determine the centre of the fragment cross sections for the loss of 3 and 4 water molecules for $[Mg(H_2O)_{50}]^+$. Blue dots: data points considered for fitting, black dots: not considered, red line: Gaussian fit, gray line: noise level.

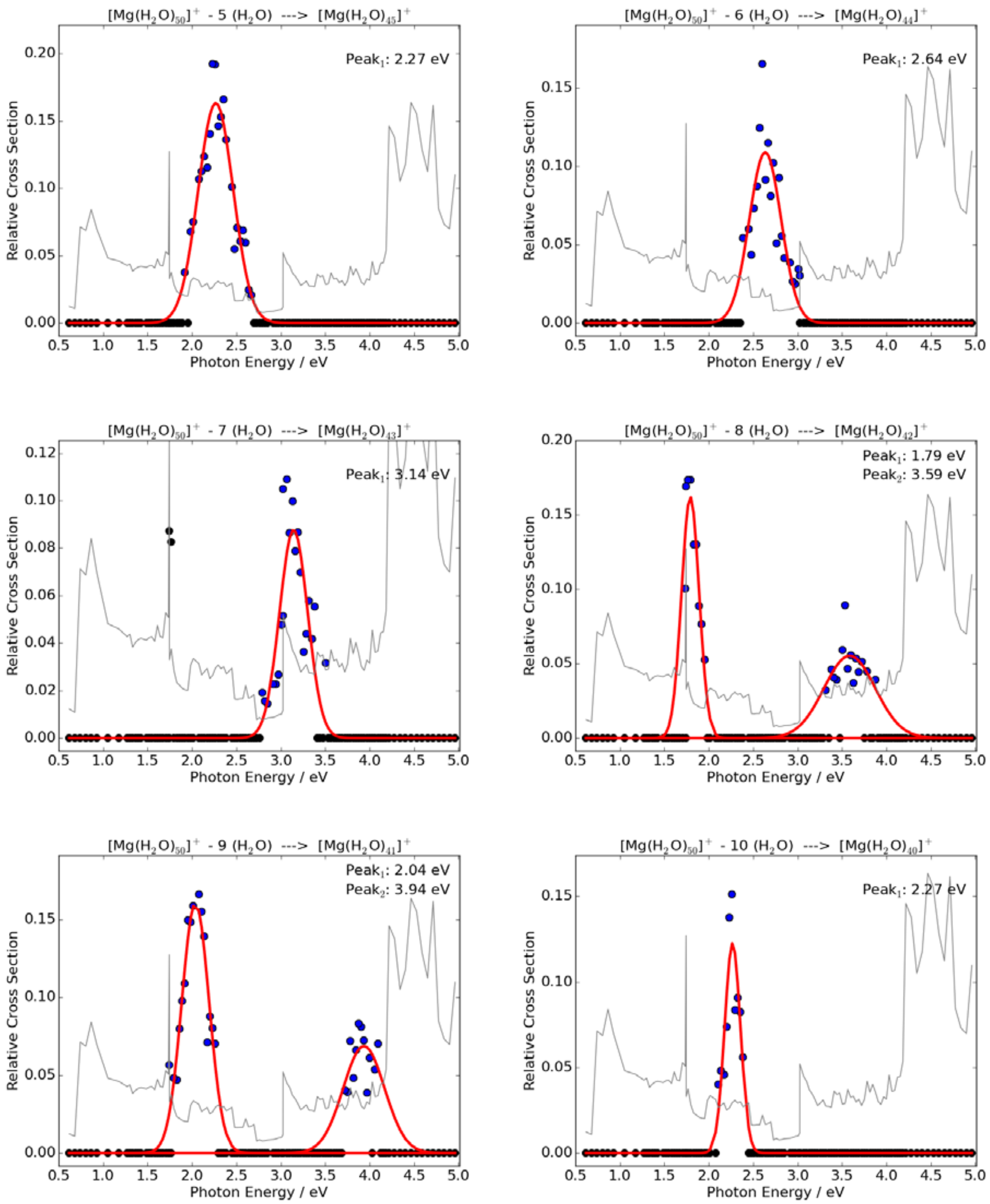

Figure S43: Fits to determine the centre of the fragment cross sections for the loss of 5, 6, 7, 8, 9 and 10 water molecules for $[Mg(H_2O)_{50}]^+$. Blue dots: data points considered for fitting, black dots: not considered, red line: Gaussian fit, gray line: noise level.

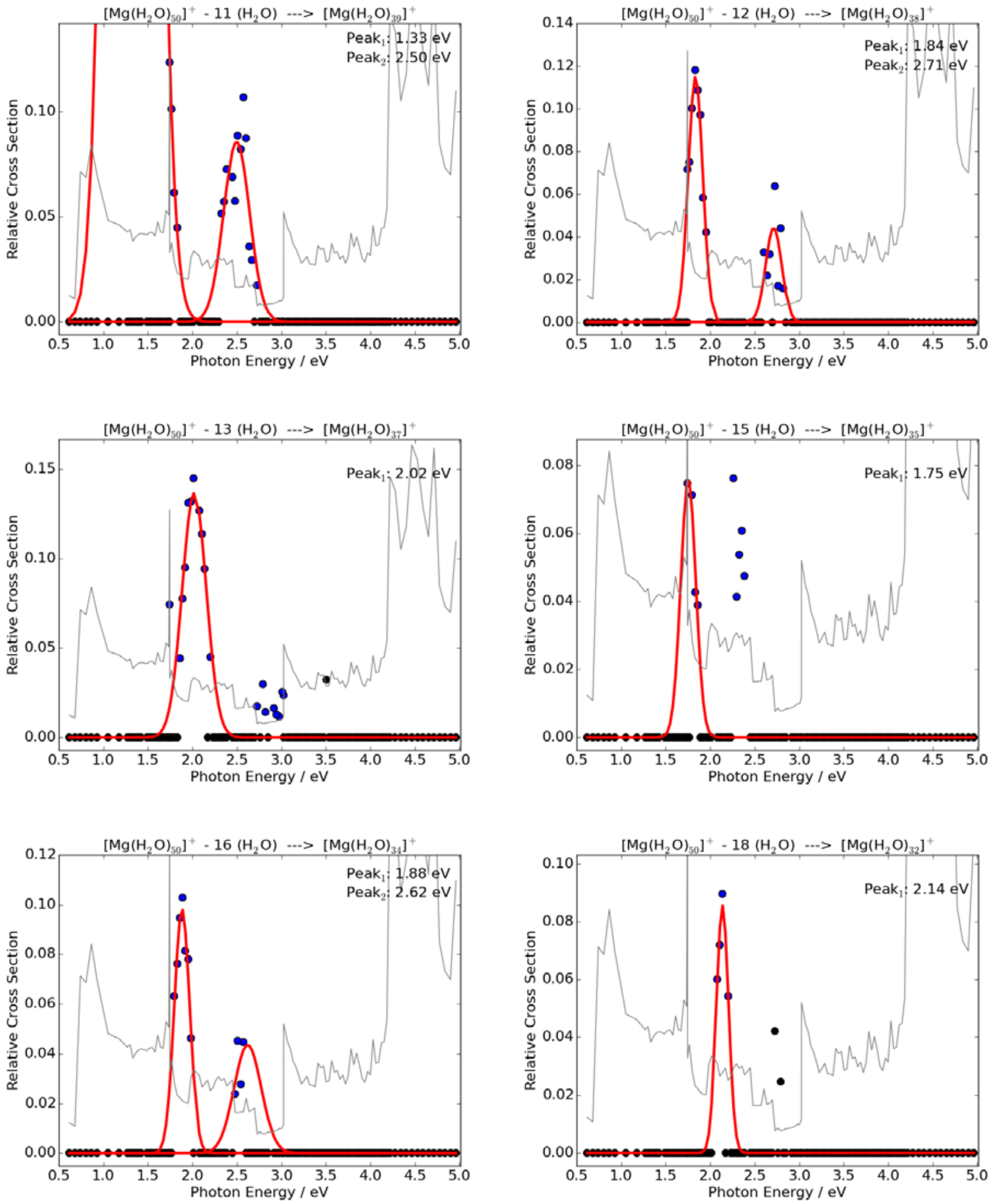

Figure S44: Fits to determine the centre of the fragment cross sections for the loss of 11, 12, 13, 15, 16 and 18 water molecules for $[Mg(H_2O)_{50}]^+$. For the loss of 11 water molecules, the fit for the lower energy data was not considered for the evaluation. The same is true for the loss of 14 water molecules (not shown in the figure). For the loss of 13 water molecules, the fit of the data points between 2.5 – 3.0 eV did not converge. For the loss of 15 water molecules, the fit of the data points around 2.8 eV did not converge. The fit of de date for the loss of 17 water molecules did also not converge (not shown in the figure). Blue dots: data points considered for fitting, black dots: not considered, red line: Gaussian fit, gray line: noise level.

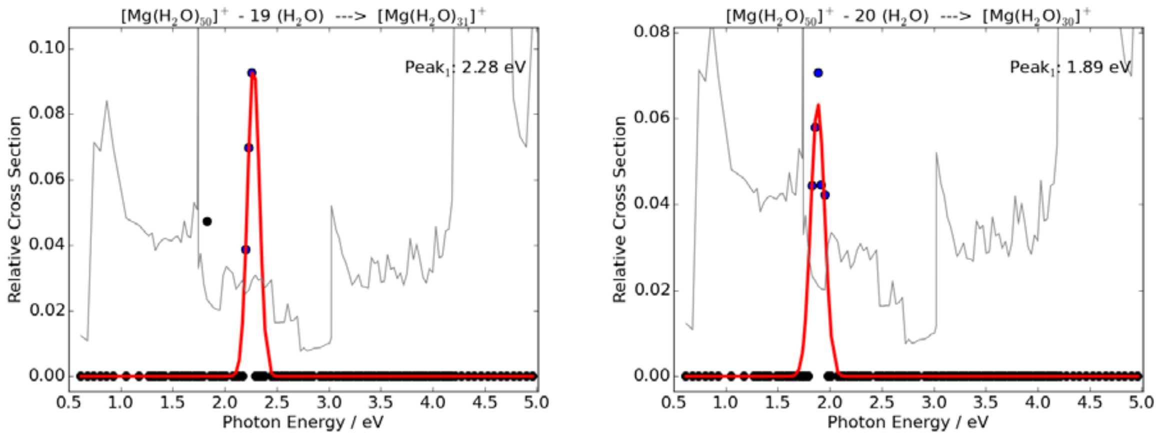

Figure S45: Fits to determine the centre of the fragment cross sections for the loss of 19 and 20 water molecules for $[Mg(H_2O)_{50}]^+$. Blue dots: data points considered for fitting, black dots: not considered, red line: Gaussian fit, gray line: noise level.

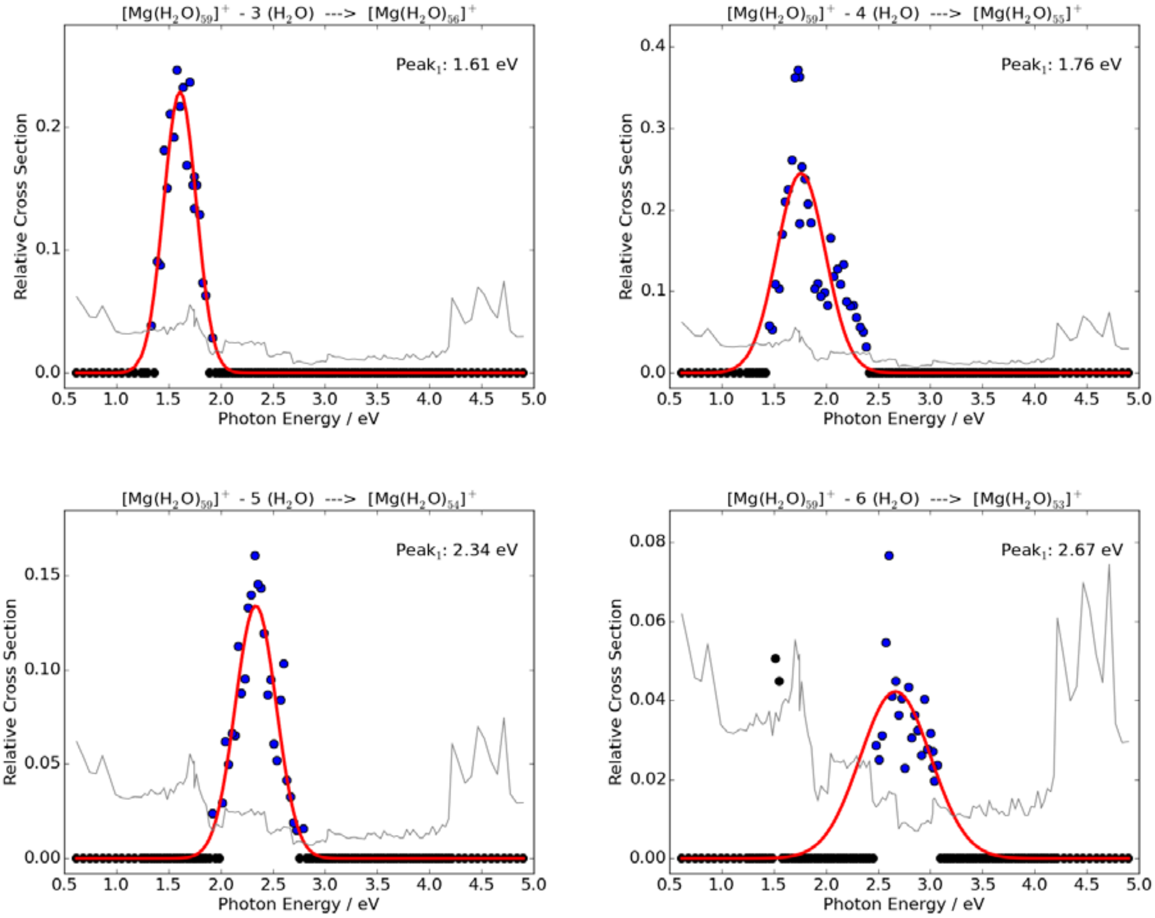

Figure S46: Fits to determine the centre of the fragment cross sections for the loss of 3, 4, 5 and 6 water molecules for $[Mg(H_2O)_{59}]^+$. Blue dots: data points considered for fitting, black dots: not considered, red line: Gaussian fit, gray line: noise level.

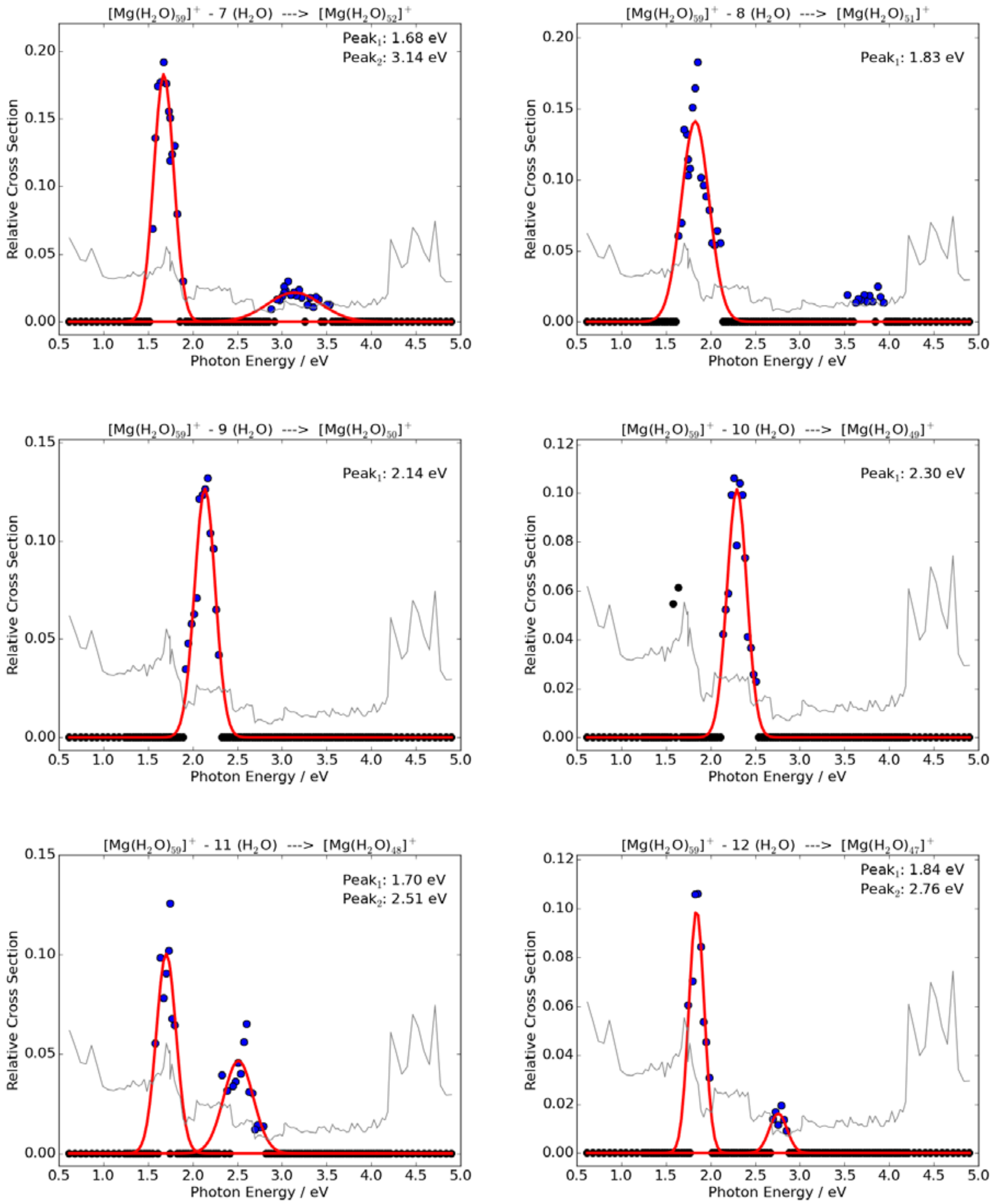

Figure S47: Fits to determine the centre of the fragment cross sections for the loss of 7, 8, 9, 10, 11 and 12 water molecules for $[Mg(H_2O)_{59}]^+$. For the loss of 8 water molecules, the fit of the data points between 3.5 – 4.0 eV did not converge. Blue dots: data points considered for fitting, black dots: not considered, red line: Gaussian fit, gray line: noise level.

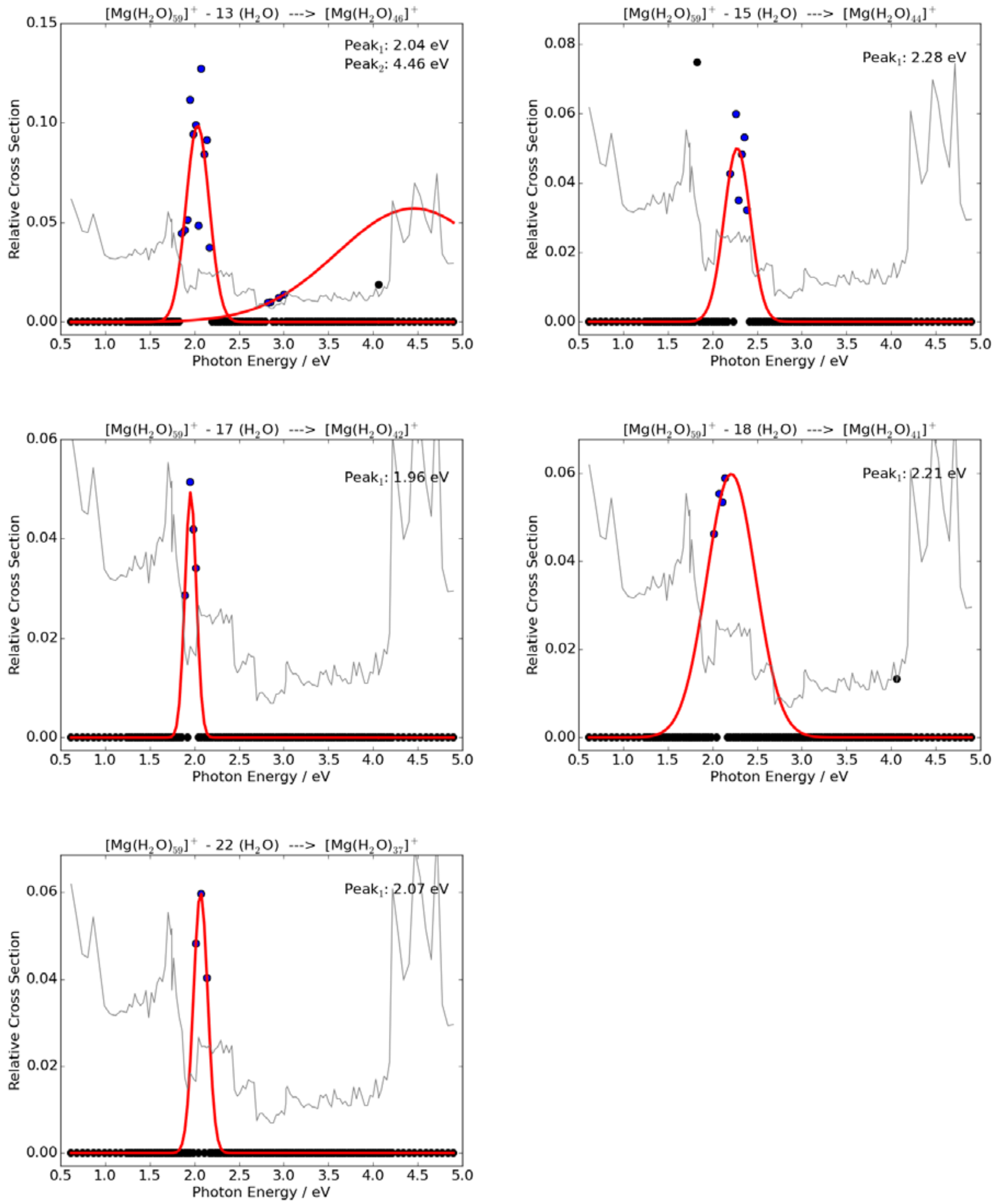

Figure S48: Fits to determine the centre of the fragment cross sections for the loss of 13, 15, 17, 18, and 22 water molecules for [Mg(H_2O)$_{59}$]$^+$. For the loss of 13 water molecules the fit for the higher energy data was not considered for the evaluation. For the loss of 14 water molecules the fit did not match well with the data and was not considered as well (not shown in the figure). For the loss of 16, 19, 20 and 21 water molecules there were too few data points for fitting (all not shown in the figure). Blue dots: data points considered for fitting, black dots: not considered, red line: Gaussian fit, gray line: noise level.

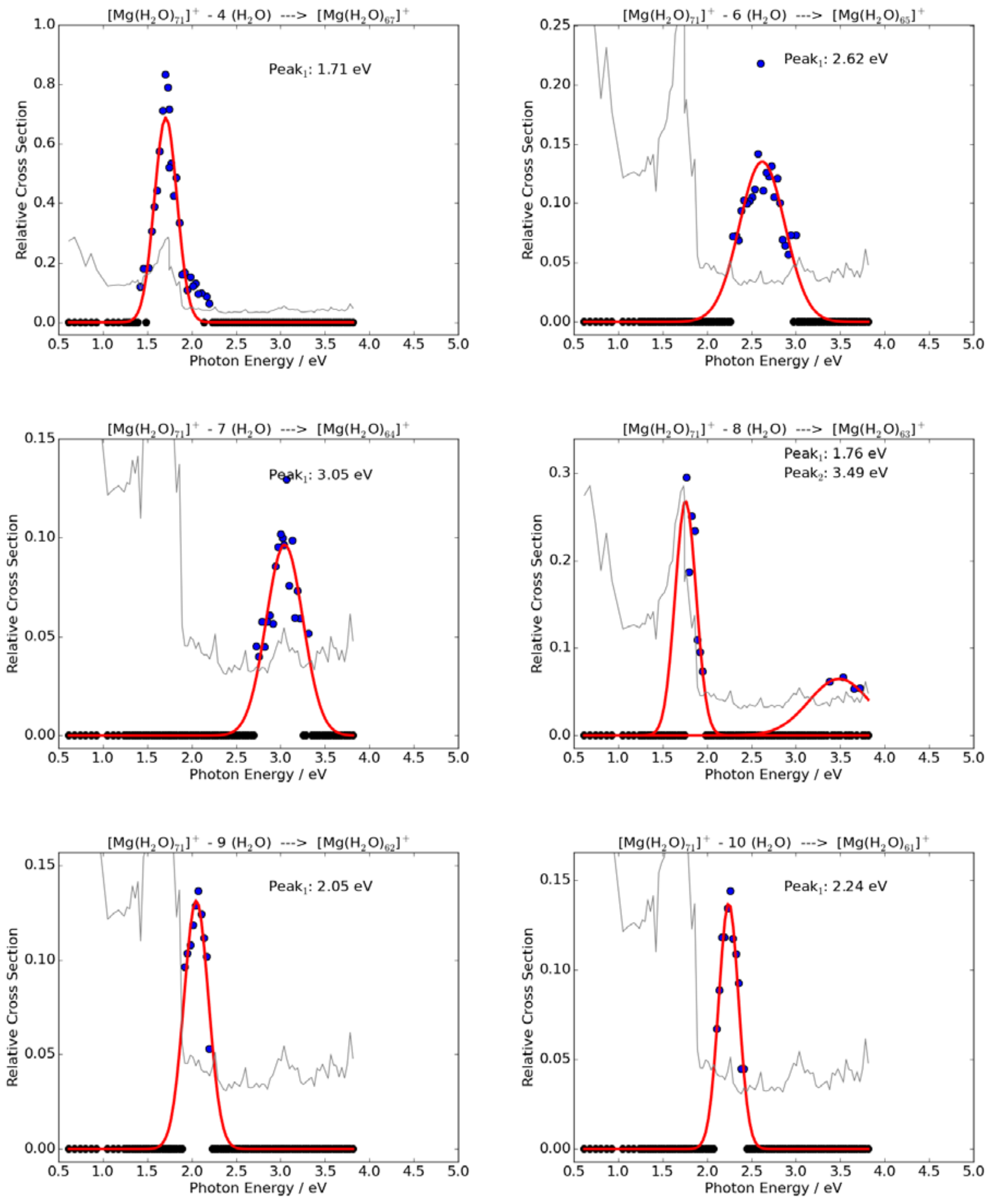

Figure S49: Fits to determine the centre of the fragment cross sections for the loss of 4, 6, 7, 8, 9 and 10 water molecules for $[Mg(H_2O)_{71}]^+$. For the loss of 3 water molecules the fit did not converge (not shown in the figure). Blue dots: data points considered for fitting, black dots: not considered, red line: Gaussian fit, gray line: noise level.

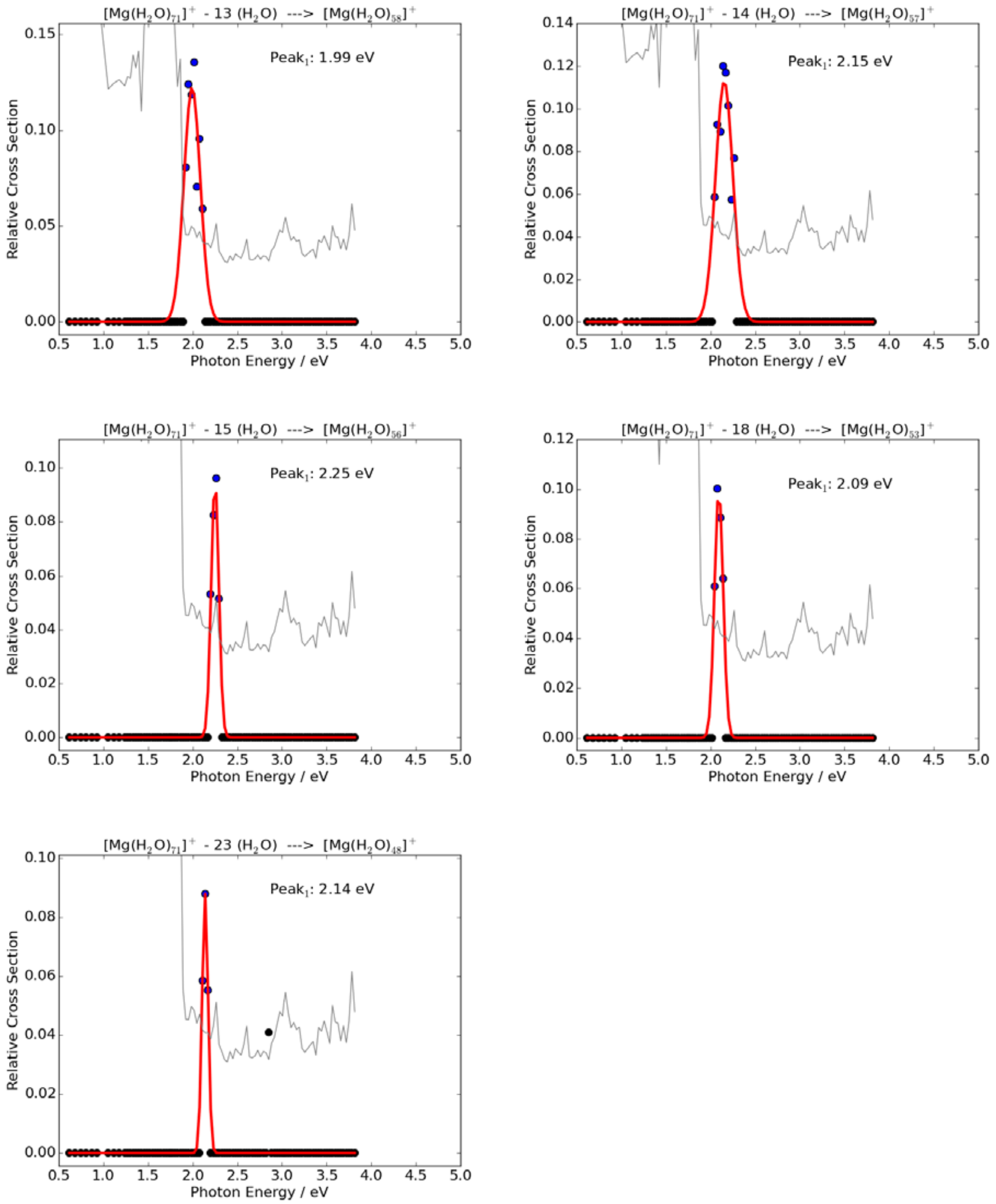

Figure S50: Fits to determine the centre of the fragment cross sections for the loss of 13, 14, 15, 18, and 23 water molecules for $[Mg(H_2O)_{71}]^+$. For the loss of 12, 17 and 19 water molecules the fits did not converge (all not shown in the figure). For the loss of 16, 20, 21 and 22 water molecules there were too few data points for fitting (all not shown in the figure). Blue dots: data points considered for fitting, black dots: not considered, red line: Gaussian fit, gray line: noise level.

7. Correction of discontinuities in the spectra

At the wavelength where the laser system switches from SF/SH (Sum Frequency / Second Harmonic) generated beam to signal beam and from signal beam to idler beam, discontinuities appear in the photodissociation cross sections. This is most likely due to a spatial deviation of the different beams. To correct this, data points were recorded as close as possible on either side of the wavelength where beams change. The cross sections were adjusted to meet at the same level at this specific wavelength. This is accomplished by multiplying the intensities outside the signal beam by a constant factor. The results are shown in Figures S51 – S55.

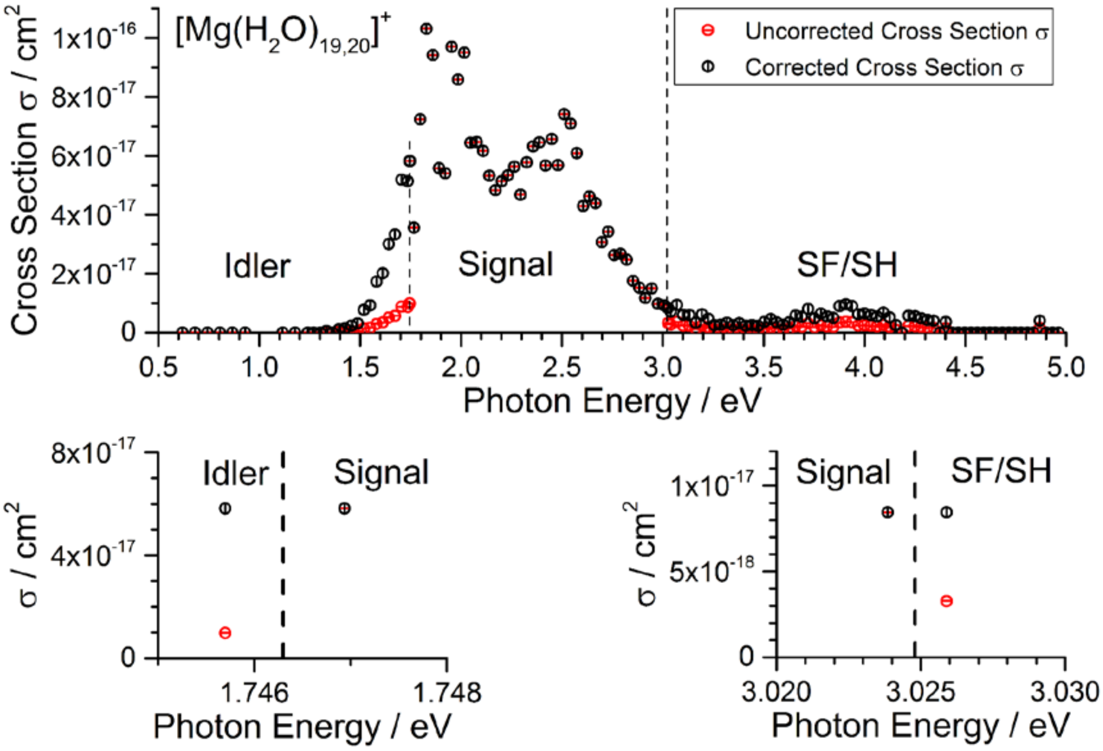

Figure S51: Discontinuity correction in the spectrum of $[Mg(H_2O)_{19,20}]^+$.

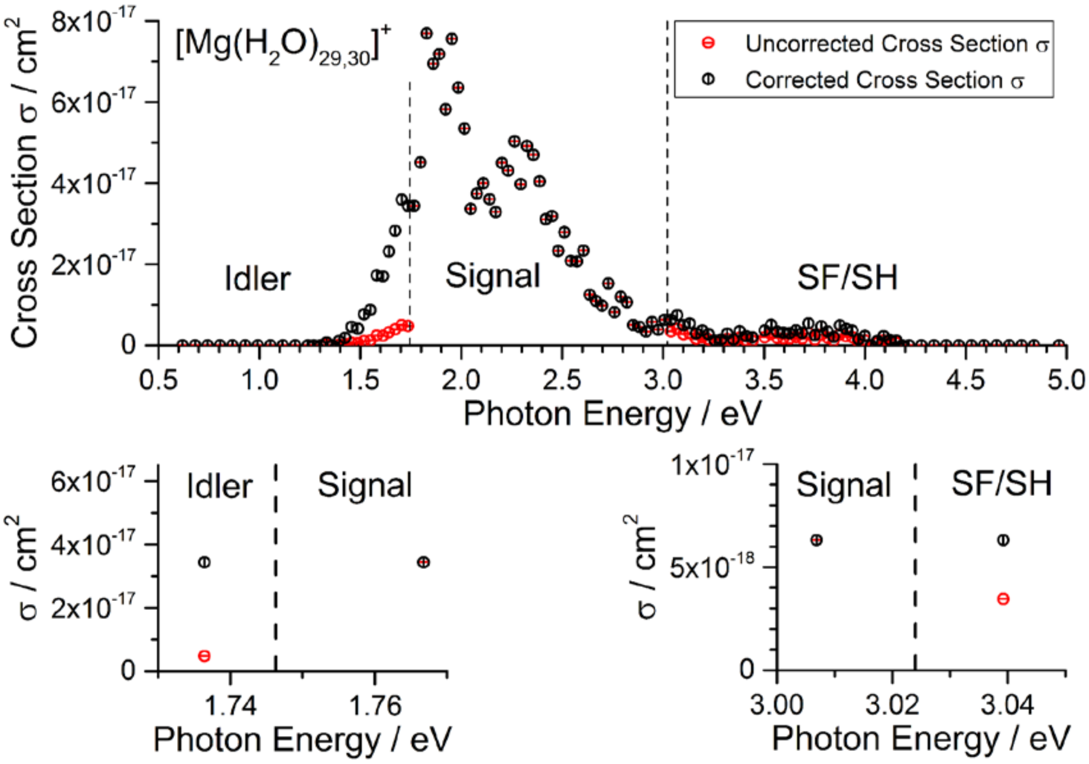

Figure S52: Discontinuity correction in the spectrum of $[Mg(H_2O)_{29,30}]^+$.

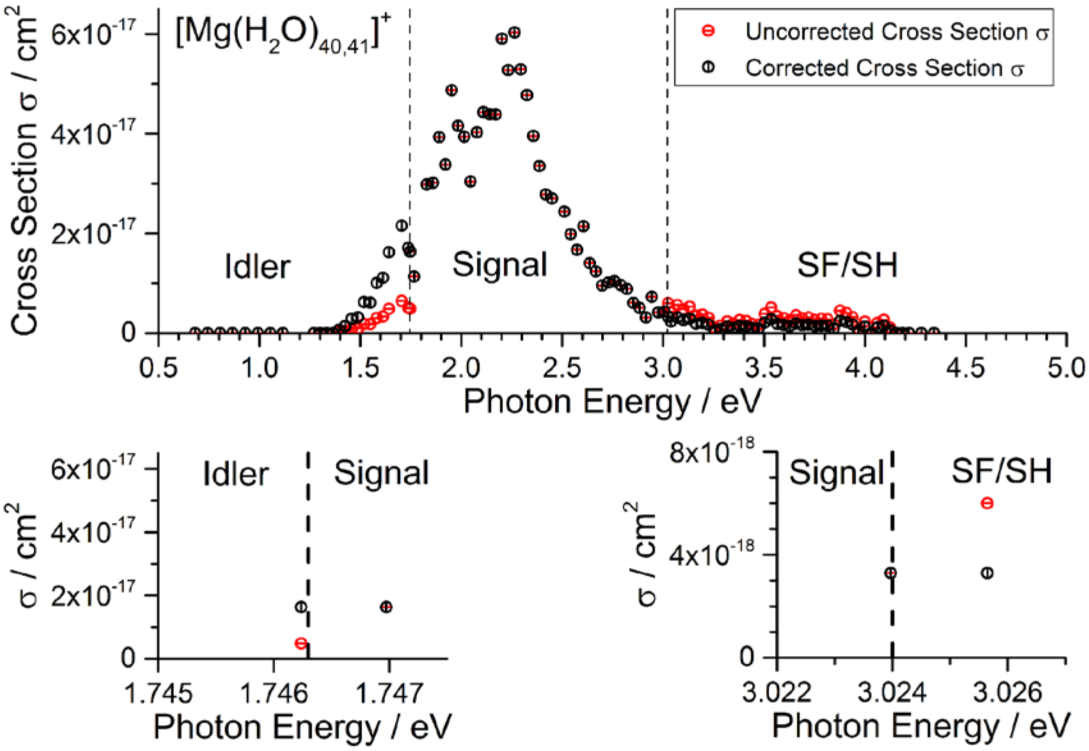

Figure S53: Discontinuity correction in the spectrum of $[Mg(H_2O)_{40,41}]^+$.

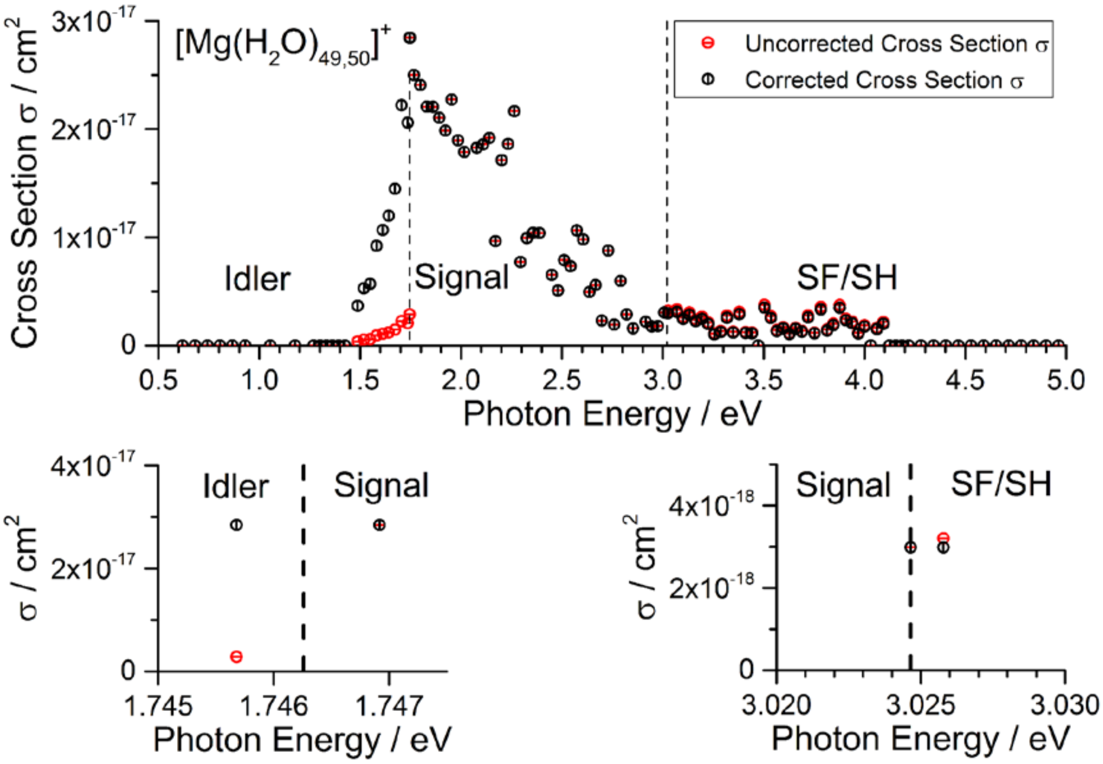

Figure S54: Discontinuity correction in the spectrum of $[Mg(H_2O)_{49,50}]^+$.

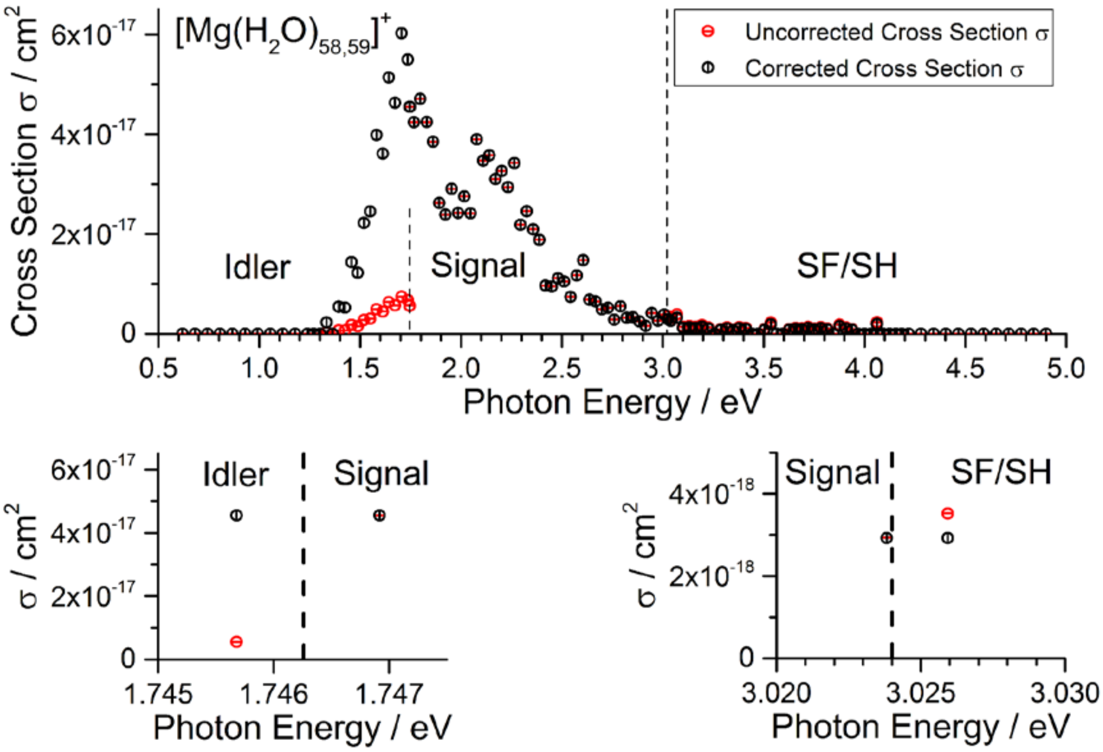

Figure S55: Discontinuity correction in the spectrum of $[Mg(H_2O)_{58,59}]^+$.

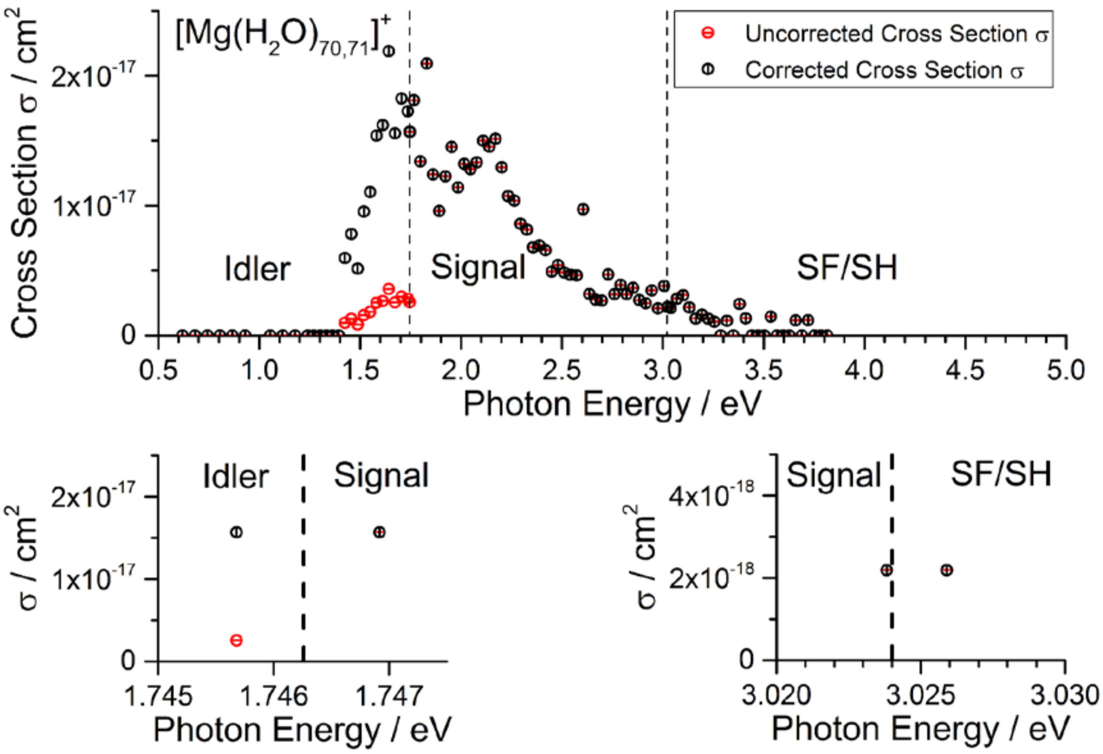

Figure S56: Discontinuity correction in the spectrum of $[Mg(H_2O)_{70,71}]^+$.

8. Cartesian coordinates (in Å) and energies (in Hartree) of calculated structures

D0 minima, ωB97XD/def2TZVP

[Mg(H2O)3]+, 3x
E=-429.155727
O 1.167632 1.264806 -0.330608
Mg -0.000361 0.000133 0.920624
O 0.512065 -1.643189 -0.330512
O -1.679329 0.378271 -0.330978
H -2.586926 0.270557 -0.028947
H -1.684662 1.062710 -1.007849
H 1.529225 2.104284 -0.028843
H 1.761953 0.926804 -1.008234
H -0.078197 -1.989930 -1.007245
H 1.059996 -2.375122 -0.029586

[Mg(H2O)4]+, 3x
E=-505.597817
O -0.317711 1.416053 -0.107758
Mg 1.037295 0.000367 -0.885727
O -0.317502 -1.415848 -0.108297
O -2.676358 -0.000255 0.354434
H -1.242193 1.177472 0.081813
H -0.274347 2.360043 -0.277805
H -1.242052 -1.177395 0.081145
H -0.274097 -2.359722 -0.278980
H -3.381403 -0.000069 -0.299836
H -3.108135 -0.000593 1.213761
O 2.285655 -0.000323 0.812202
H 3.245855 -0.000011 0.763393
H 2.036157 -0.001141 1.740588

[Mg(H2O)4]+, 4x
E=-505.594904
O 0.000154 1.657631 0.642318
Mg -0.000051 -0.012473 -0.690522
O -2.179515 -0.196217 -0.419362
O -0.000003 -1.305462 0.987922
H 0.000120 1.616498 1.602975
H 0.000051 2.583351 0.384071
H -0.778172 -1.826176 1.206447
H 0.778274 -1.826014 1.206452
H -2.750951 0.460456 -0.007990
H -2.632959 -0.497838 -1.214527
O 2.179439 -0.196335 -0.419414
H 2.632864 -0.498156 -1.214514
H 2.750779 0.460623 -0.008362

[Mg(H2O)5]+, 3x
E=-582.035124
O 0.747793 0.206319 0.158550
Mg -0.559841 -1.032340 -0.839153
O -2.038395 0.400558 -0.414967
O -0.862027 -2.250932 0.859286
O -0.688120 2.664444 0.431339
H 0.521538 1.131343 0.335087
H 1.726989 0.135655 0.080475
H -1.796754 1.301460 -0.133982
H -2.919937 0.418555 -0.793246
H -0.516531 3.387256 -0.178688
H -0.847335 3.068808 1.288556
H -1.071074 -3.188120 0.824556
H -0.686696 -2.016823 1.774711
O 3.396010 0.032639 -0.121662
H 3.823243 -0.071551 -0.973812
H 4.042555 -0.202733 0.545819

[Mg(H2O)5]+, 4x
E=-582.034554
O -1.389643 1.426178 0.869800
Mg -0.744555 0.148310 -0.711898
O 1.082420 1.354696 -0.500098
O -2.409146 -1.155971 -0.131586
O 0.388437 -1.258562 0.363274
H -0.749019 2.079001 1.168470
H -2.256276 1.840141 0.897720
H 0.091288 -2.165719 0.459469
H 1.359866 -1.239729 0.398076
H -2.875972 -1.257314 0.703879
H -3.009810 -1.415371 -0.837592
H 1.924773 0.908748 -0.306222
H 1.239142 1.959709 -1.231409
O 3.071574 -0.516691 0.235408
H 3.604825 -0.397082 1.026232
H 3.656703 -0.889304 -0.430221

[Mg(H2O)5]+, 5x
E=-582.032394
O -0.000927 0.000184 1.660566
Mg 0.000072 -0.000077 -0.474014
O 1.895213 -1.015017 -0.406223
O -1.894650 1.015410 -0.406938
O -1.072114 -1.867747 -0.104432
H -0.380031 -0.683653 2.216873
H 0.379679 0.682872 2.217252
H -1.989215 -1.831176 -0.398666
H -0.686943 -2.635493 -0.541059
H -2.413294 1.450861 0.278346
H -2.254938 1.259673 -1.267628
H 2.254751 -1.259370 -1.267224
H 2.413876 -1.451246 0.278564
O 1.072311 1.867418 -0.104177
H 0.687269 2.635698 -0.539954
H 1.989317 1.830774 -0.398691

[Mg(H2O)6]+, 3x
E=-658.472760
O 0.988816 1.600981 0.664343
Mg 2.148326 -0.003610 0.000292
O 0.980182 -1.378377 1.054808
O 0.982194 -0.226531 -1.718848
O -1.713746 -0.685231 -1.459062
O -1.714873 -0.917436 1.324666
O -1.706598 1.610468 0.134666
H 1.339929 -2.145355 1.504449
H 0.038717 -1.280092 1.298604
H 1.353605 2.379113 1.090111
H 0.047317 1.763941 0.457913
H 0.040598 -0.486459 -1.755521
H 1.343900 -0.240554 -2.607012
H -2.280301 -1.081550 -2.124964

H -1.883118 -1.142409 -0.619200
H -2.281661 -1.295043 2.001169
H -1.879543 0.039335 1.303544
H -2.270968 2.386895 0.125632
H -1.876185 1.114505 -0.682806

[Mg(H2O)6]+, 4x
E=-658.473418
Mg -0.000432 -0.000413 0.933268
O -1.016378 -1.227582 -0.431209
O 1.654302 -1.382189 0.574965
O -1.654240 1.382308 0.574277
O 1.016806 1.227591 -0.429688
O 3.624815 0.268522 -0.667784
H -0.696420 -2.094065 -0.688359
H -1.963318 -1.154444 -0.630947
H 1.911153 -1.858480 1.370699
H 2.466372 -1.025600 0.177235
H 1.963362 1.153096 -0.630761
H 0.697412 2.094333 -0.686594
H 4.332654 0.632735 -0.129102
H 4.020434 0.037021 -1.512405
H -2.465552 1.025889 0.174773
H -1.912594 1.857947 1.369897
O -3.624695 -0.268019 -0.667326
H -4.332546 -0.630110 -0.127224
H -4.020650 -0.038414 -1.512314

[Mg(H2O)6]+, 5x
E=-658.469659
O 2.122740 -1.392229 0.205332
Mg 0.638247 -0.040327 -0.511061
O -0.979034 -1.610027 -0.814535
O 1.878348 1.592283 0.143371
O -0.132879 -0.047199 1.472157
O -0.829409 1.477506 -0.906646
O -2.741606 0.126537 0.603460
H -1.104251 0.009859 1.507917
H 0.221743 0.573591 2.112757
H 1.595763 2.468295 -0.138359
H 2.827935 1.542437 -0.001733
H 2.259509 -1.813274 1.060507
H 2.645584 -1.857765 -0.456563
H -0.876987 -1.660414 -1.781295
H -0.762655 -2.492356 -0.486075
H -1.697636 1.315736 -0.497847
H -0.971953 1.686006 -1.835495
H -3.632210 0.217846 0.949595
H -2.709093 -0.681011 0.074210

[Mg(H2O)6]+, 6x
E=-658.469612
O 1.245563 0.963203 1.398163
Mg -0.010449 0.001156 0.000520
O -1.106315 1.778369 0.101035
O -1.163480 -0.708699 -1.593551
O -1.240978 -0.934037 1.408933
O 1.307401 0.628956 -1.527960
O 1.167093 -1.736650 0.217911
H 1.855627 -2.072361 -0.360691
H 0.757327 -2.481351 0.669123
H -2.047160 1.457093 0.176306
H -1.113388 2.452199 -0.592939
H 0.958342 0.622194 -2.424786
H 2.016666 1.274270 -1.482903
H 0.897862 1.758307 1.814161
H 1.904354 0.578055 1.980544
H -2.154103 -0.800695 1.032109
H -1.299210 -0.675508 2.339180
H -2.085060 -0.413949 -1.354281
H -1.240154 -1.641273 -1.838303

[Mg(H2O)7]+, 3x
E=-734.909239
O 0.231298 0.003929 -1.531018
Mg 2.201852 0.098635 -0.879782
O 1.734835 -1.488900 0.404661
O 1.610655 1.627685 0.427590
O -1.868888 -1.597013 -0.739895
O -0.656425 -1.260763 1.745084
O -0.964294 1.464919 1.407113
O -2.326448 1.143259 -0.908486
H 2.167813 2.347922 0.728213
H 0.730476 1.701138 0.846761
H -0.354984 0.752290 -1.696135
H -0.361842 -0.761012 -1.419098
H 0.954727 -1.509731 0.994197
H 2.364798 -2.153459 0.689971
H -0.854190 -1.646365 2.601507
H -0.802930 -0.296128 1.816741
H -1.323793 2.071580 2.058530
H -1.555677 1.492933 0.622442
H -2.256051 -2.407358 -1.076688
H -1.615298 -1.745516 0.187145
H -3.056967 1.628277 -1.297134
H -2.544155 0.196879 -0.939461

[Mg(H2O)7]+, 4x
E=-734.906089
O 1.025607 1.000093 -0.040457
Mg 0.140618 -0.562898 0.922534
O -0.759824 -1.557861 -0.695305
O 1.898887 -1.697652 0.384801
O -1.793603 0.665569 0.940546
O 3.701636 0.222233 -0.612367
O -3.487000 -0.953414 -0.691676
H 0.557120 1.843540 -0.230700
H 1.940357 1.032662 -0.347038
H -2.015099 0.643726 1.880518
H -2.527197 0.228481 0.471445
H -1.716222 -1.570594 -0.858532
H -0.355910 -2.328644 -1.098419
H -4.134836 -1.539736 -0.291282
H -3.910272 -0.569085 -1.464267
H 2.666192 -1.203230 0.045709
H 2.216661 -2.308177 1.055860
H 4.410436 0.562170 -0.059842
H 4.059590 0.159871 -1.501672
O -0.690482 2.986738 -0.373116
H -1.364020 2.561410 0.171150
H -0.675972 3.916733 -0.142754

[Mg(H2O)7]+, 5x
E=-734.905436
O 1.466943 1.719698 -0.197744
Mg -0.378911 0.720883 -0.596631
O -2.288146 -0.250041 -0.608922
O -1.355337 2.301986 0.522423

O -0.117859 -0.151075 1.365767
O 0.692494 -1.215057 -1.085926
O -1.204727 -2.539470 0.531400
H -0.507991 -1.039080 1.438700
H -0.461394 0.384153 2.085637
H -2.301346 2.394346 0.371083
H -0.976365 3.185319 0.479275
H 2.196046 1.125657 0.050806
H 1.752328 2.209389 -0.977921
H 0.745978 -1.172705 -2.051035
H 1.605512 -1.165585 -0.745195
H -2.285584 -1.161287 -0.267576
H -2.709151 -0.256397 -1.475079
H -1.440697 -3.425293 0.813970
H -0.513852 -2.609385 -0.142548
H 3.093107 -0.845232 1.194982
O 3.100202 -0.565069 0.275584
H 4.001792 -0.682275 -0.036185

[Mg(H2O)7]+, 6x
E=-734.907133
O 1.850849 1.008906 -1.207236
Mg 0.451692 0.021476 -0.006147
O -0.776189 -1.112344 1.272570
O -0.859425 1.725156 0.123292
O 1.553186 -1.749364 -0.397325
O -0.834024 -0.530789 -1.585835
O -3.018265 -0.083677 0.036637
H -1.773623 -0.477150 -1.333678
H -0.713194 -1.314053 -2.126547
H 1.413144 -2.570355 0.080987
H 2.459657 -1.736402 -0.714899
H 1.708382 1.254924 -2.129588
H 2.053217 1.836484 -0.720490
H -0.365061 2.291229 0.787390
H -0.839561 2.257258 -0.692401
H -1.727042 -0.999064 1.100767
H -0.624930 -0.967420 2.211380
H -3.977555 -0.114469 0.043006
H -2.745367 0.823726 0.238892
O 1.554107 0.461366 1.714789
H 2.489228 0.255087 1.855722
H 1.460495 1.448458 1.818092

[Mg(H2O)8]+, 3x
E=-811.342998
O -1.212463 1.567336 -1.258536
Mg -2.455058 0.668286 0.155562
O -0.872855 0.654599 1.496085
O -2.052932 -1.338777 -0.261816
O 1.522742 1.733450 -1.023356
O 0.966054 -1.323869 1.946183
O 0.443241 -2.426345 -0.507216
H -2.759042 -1.950475 -0.479844
H -1.192549 -1.782237 -0.425413
H -1.545273 2.051642 -2.016591
H -0.238737 1.671453 -1.220660
H -0.401372 -0.152832 1.779165
H -0.276324 1.380614 1.709866
H 1.052377 -1.918256 2.693943
H 0.901665 -1.870284 1.137022
H 0.539515 -3.353875 -0.733548
H 0.968868 -1.908718 -1.168388
H 1.976624 2.508143 -1.362463
H 1.777362 1.640219 -0.079168
O 1.748824 -0.822119 -2.144871
H 2.451896 -0.986721 -2.773143
H 1.801031 0.106632 -1.866441
O 1.943033 1.275597 1.620564
H 2.618094 1.661430 2.181714
H 1.901405 0.324846 1.820895

[Mg(H2O)8]+, 4x
E=-811.338274
O -0.307600 -1.550353 -0.177478
Mg 0.000195 0.000194 1.121905
O 0.307499 1.549946 -0.178703
O -2.218146 0.383207 0.947613
O 2.218218 -0.383184 0.947030
O -3.108137 -1.767503 -0.631020
O 3.108358 1.768560 -0.630661
H 0.429542 -2.106586 -0.512790
H -1.158262 -1.865935 -0.505819
H 2.533425 -0.395720 1.858557
H 2.697730 0.330016 0.483972
H 1.158239 1.866642 -0.505746
H -0.429739 2.106179 -0.513850
H 3.609752 2.510653 -0.282815
H 3.489140 1.562621 -1.488483
H -2.697828 -0.330384 0.485313
H -2.532716 0.396302 1.859355
H -3.609611 -2.510097 -0.284361
H -3.489030 -1.560004 -1.488421
O 2.017944 -2.659140 -0.809849
H 2.485757 -2.155611 -0.132842
H 2.346337 -3.558917 -0.780104
O -2.018405 2.658092 -0.809759
H -2.485173 2.154002 -0.132422
H -2.347746 3.557509 -0.779793

[Mg(H2O)8]+, 5x
E=-811.339818
O -2.438667 -1.001288 0.156873
Mg -0.975368 0.464701 0.753788
O 0.445402 2.044425 0.756314
O -2.213526 1.856373 -0.383084
O -0.166409 0.044032 -1.296684
O 0.471429 -1.184002 1.280713
O 2.264625 1.225198 -1.089510
H 0.727909 0.431815 -1.423457
H -0.749862 0.487937 -1.920203
H -2.060824 2.780216 -0.159319
H -3.164570 1.713178 -0.349338
H -2.073663 -1.827732 -0.204561
H -3.029178 -1.233366 0.881785
H 0.214162 -1.340056 2.201234
H 0.235064 -1.980504 0.765154
H 1.238475 2.002911 0.186133
H 0.689480 2.410555 1.610884
H 2.834748 1.653793 -1.729968
H 2.794365 0.553440 -0.597125
H -0.190810 -2.038939 -1.418087
O -0.484897 -2.721598 -0.799438
H -0.346951 -3.577882 -1.209616
H 2.380211 -0.963301 0.957962
O 3.155352 -0.730460 0.426863
H 3.939393 -0.909910 0.946706

[Mg(H2O)8]+, 6x
E=-811.345959
O 1.945090 -1.478468 0.854897
Mg 0.777018 0.033514 0.008728
O -0.265003 1.685412 -0.686468
O -0.342693 -1.340543 -1.229447
O 1.873708 1.361152 1.232752
O -0.638405 -0.194908 1.535916
O -2.703324 -1.391252 0.243467
H -1.470442 -0.656326 1.308717
H -0.851290 0.504808 2.156395
H 1.833782 2.319466 1.238567
H 2.645659 1.079799 1.729689
H 1.596913 -2.020840 1.573788
H 2.130061 -2.098334 0.101126
H -0.493211 -0.998448 -2.128109
H 0.363697 -2.063135 -1.360326
H -1.244736 1.689967 -0.557066
H -0.072381 2.050283 -1.552547
H -3.309900 -2.107459 0.442513
H -2.056915 -1.713071 -0.408530
O 2.116559 0.169703 -1.601863
H 3.012083 0.524431 -1.549052
H 2.200347 -0.761322 -1.935040
H -3.097563 0.443319 -0.131438
O -2.846198 1.378440 -0.179271
H -3.628190 1.888411 -0.393286

[Mg(H2O)10]+, 3x
E=-964.210655
O -0.941217 0.522077 -1.242204
Mg -0.147814 2.398450 -0.986288
O -0.884254 2.451649 0.994495
O 1.598219 1.547246 -0.306607
O -1.293252 0.008637 2.256051
O -2.124965 -2.040917 0.632702
O 1.483571 0.059956 2.119219
O 0.105851 -2.077968 -0.970411
O 2.424446 -1.964120 0.506311
H -1.335177 3.211659 1.366591
H -1.195206 1.641729 1.441908
H -1.858604 0.260413 -1.470391
H -0.418433 -0.296574 -1.246385
H 1.633047 1.190069 0.594947
H 2.227967 1.019148 -0.845163
H 1.895048 0.400817 2.916123
H 0.522421 0.004594 2.289670
H -1.764193 -0.033260 3.091229
H -1.648634 -0.717688 1.696771
H 0.200795 -2.525596 -1.813915
H 0.964028 -2.147744 -0.505731
H -2.395297 -2.884525 1.000793
H -1.362871 -2.217699 0.044279
H 2.850016 -2.713347 0.927083
H 2.189878 -1.325642 1.208158
O 3.145283 -0.227348 -1.590904
H 3.232183 -0.949365 -0.953107
H 3.998423 -0.093468 -2.005931
O -3.313204 -0.648914 -1.559062
H -3.362669 -1.193443 -0.764207
H -4.202781 -0.453855 -1.853985

[Mg(H2O)10]+, 4x
E=-964.213298
O 0.001680 1.439946 -0.393277
Mg -0.000372 -0.127113 -1.728936
O -0.002036 -1.684926 -0.370831
O 2.255852 -0.168006 -1.519237
O -2.256848 -0.162842 -1.518772
O -2.582014 -1.881086 0.528755
O 2.635729 2.059392 0.133544
O -2.630584 2.062977 0.137373
O 2.576557 -1.883014 0.531791
H 0.802610 1.876459 -0.059448
H -0.798054 1.877582 -0.058018
H 2.602989 -0.347665 -2.400031
H 2.558340 -0.886054 -0.916957
H 0.796664 -1.941003 0.122149
H -0.801825 -1.941330 0.120177
H 3.126493 -2.657929 0.656732
H 2.770320 -1.245677 1.259993
H -2.604788 -0.339535 -2.399845
H -2.561225 -0.881326 -0.917953
H -3.101148 2.878091 -0.047879
H -2.793833 1.455177 -0.602990
H 2.797398 1.450660 -0.606377
H 3.107067 2.873751 -0.053057
H -3.134512 -2.654437 0.652048
H -2.773824 -1.244517 1.258099
O 2.899627 0.152441 2.196809
H 2.912831 0.968497 1.673797
H 3.396108 0.307027 3.000384
O -2.897816 0.152593 2.197142
H -2.909243 0.970001 1.676239
H -3.389076 0.307777 3.003790

[Mg(H2O)10]+, 5x
E=-964.207177
O 0.157852 -2.512928 -0.910489
Mg 0.311754 -0.377737 -1.038192
O -0.199919 -0.391603 1.051369
O 0.479465 1.801471 -0.862874
O 2.409536 -0.608386 -0.556203
O -1.958536 -0.249851 -1.283827
O 2.012365 -1.232033 2.188724
O -0.978950 2.233573 1.362635
O -3.287057 1.779583 0.183748
O -2.169961 -2.294178 0.544136
H -0.460692 0.484455 1.395666
H 0.546639 -0.726117 1.609128
H 2.888088 0.261040 -0.526330
H 2.842651 -1.143388 -1.228925
H -0.634917 -2.833684 -0.445498
H 0.177025 -2.929028 -1.778860
H -1.980534 -0.383523 -2.244566
H -2.334251 -1.046238 -0.859351
H -0.028686 2.186756 -0.113591
H 0.140265 2.172122 -1.686449
H -0.888028 2.857459 2.084510
H -1.918111 2.230205 1.062943
H -1.681114 -1.688126 1.123944
H -2.819548 -2.763448 1.070472
H -3.031022 1.161741 -0.516902
H -4.052211 2.272395 -0.113425
H 2.528427 -1.287612 1.374331
H 2.272152 -1.956388 2.758678
O 3.302771 1.854108 -0.359147
H 4.053880 2.327798 -0.719361

H 2.498405 2.298368 -0.652688

[Mg(H2O)10]+, 6x
E=-964.220470
O -2.634942 -0.497501 -1.387676
Mg -1.222148 0.048953 0.052512
O -2.789348 0.254179 1.435627
O 0.042283 0.622215 1.609554
O -1.015353 -1.998535 0.717951
O -1.310685 2.058127 -0.563777
O 0.350675 -0.188544 -1.274221
O 1.300355 -2.678833 -0.680841
O 2.609961 1.304349 1.054800
H 0.856714 -1.017670 -1.319030
H 0.911271 0.568204 -1.506667
H -0.438901 2.435388 -0.783672
H -1.925989 2.309271 -1.256011
H -2.371539 -0.868272 -2.238666
H -3.190183 -1.189666 -0.934827
H -0.898672 -2.044345 1.681513
H -1.963737 -2.337183 0.558417
H 0.977514 0.905419 1.547810
H -0.347526 0.984914 2.407245
H 1.373263 -3.489782 -1.187954
H 0.483344 -2.735012 -0.150598
H -3.447627 0.957300 1.385807
H -3.283796 -0.604835 1.374618
H 3.001550 0.442892 0.767386
H 3.217534 1.704417 1.679718
O 3.420697 -1.056039 0.150753
H 4.283017 -1.468247 0.207453
H 2.771235 -1.754873 -0.033276
O 1.383105 2.362516 -1.152002
H 1.954916 2.192967 -0.377990
H 1.849415 2.966200 -1.732758

[Mg(H2O)12]+, 3x
E=-1117.083028
O -2.436936 -1.516525 1.413860
Mg -0.750789 -2.261648 0.205081
O 0.967037 -2.504707 -1.066785
O 0.334479 -0.713490 1.057536
O -0.401462 1.374456 -2.165351
O 2.091277 2.116582 1.218769
O -0.635108 1.723326 1.961313
O 2.187275 1.682078 -1.351825
O 3.236384 -0.846093 -1.038673
O 3.009505 -0.535788 1.693609
O -2.131044 2.406774 -0.298586
O -3.844797 0.210693 -0.270692
H -2.855015 -2.226725 1.908878
H -3.135666 -0.977185 1.005272
H -1.056795 -0.349308 -1.706467
H -2.474670 -0.709093 -1.172394
H 1.171310 -3.417119 -1.285959
H 1.792657 -1.986954 -1.090842
H 3.996756 -1.051115 -1.586295
H 2.933585 0.053031 -1.285407
H 1.245853 -0.767238 1.395907
H -0.102333 0.033891 1.501965
H 2.706004 2.375626 -1.765726
H 2.140794 1.908123 -0.376456
H 3.353081 -0.761077 0.810566
H 3.521753 -1.017350 2.345174
H 1.197977 2.143165 1.594782
H 2.533177 1.357097 1.626377
H -1.235145 2.042947 1.262318
H -1.065805 1.877523 2.803903
H -1.522983 2.146766 -1.023367
H -2.361164 3.327215 -0.442031
H -3.463144 1.105607 -0.240180
H -4.763933 0.285029 -0.531976
H 0.544368 1.491914 -1.922448
H -0.471530 1.553870 -3.105296
O -1.579068 -1.059663 -1.300835

[Mg(H2O)12]+, 4x
E=-1117.083214
O 2.870484 1.275804 0.900144
Mg 0.770994 1.034722 0.920213
O 0.716846 0.454394 -1.074350
O 0.733051 3.063426 0.405910
O -1.238735 1.308036 1.195633
O -1.829519 0.755856 -2.142475
O -2.572370 -1.674440 -1.009324
O -0.245411 -2.217633 2.245780
O -2.704493 -0.804638 1.770660
O -1.770292 3.216961 -0.747438
O 1.685998 -2.070905 -1.638336
O 3.924400 -0.746199 -0.479402
H 3.286578 1.231465 1.765677
H 3.317773 0.582209 0.355499
H 1.086148 -0.396264 -1.374733
H -0.123770 0.589862 -1.548796
H 1.519293 3.452805 0.019688
H -0.050402 3.376339 -0.080529
H -1.932550 2.472091 -1.351138
H -2.385064 3.918099 -0.967852
H -1.850262 0.582782 1.483629
H -1.747297 2.047300 0.837683
H -1.995363 0.706108 -3.086023
H -2.206764 -0.057030 -1.748158
H -2.054256 -1.395542 2.178661
H -2.873238 -1.176159 0.894196
H -1.824370 -2.280948 -0.839424
H -3.292770 -2.207458 -1.351921
H 0.102305 -2.636981 3.039587
H 0.275808 -1.370281 2.136430
H 3.280889 -1.361274 -0.862104
H 4.711513 -0.767826 -1.023945
H 1.038143 -2.606446 -1.137442
H 1.769613 -2.467353 -2.507408
O -0.387452 -3.247391 -0.225163
H -0.286610 -2.955319 0.715944
H -0.477326 -4.203010 -0.203203

[Mg(H2O)12]+, 5x
E=-1117.092432
Mg -0.472895 -0.553139 -0.796598
O -2.049312 -1.781207 -0.317802
O -0.289704 0.054146 1.209780
O 0.754266 -2.179686 -0.703762
O 1.068952 0.480691 -1.602723
O -1.800542 0.712774 -1.675483
H -2.746401 0.570218 -1.502652
H -1.623781 1.657385 -1.397534
H -2.915880 -1.354946 -0.171157
H -1.882299 -2.421041 0.387984

H -1.067637 0.612361 1.439037
H 0.488372 0.623832 1.393905
H 1.121211 1.452125 -1.508672
H 1.962091 0.094716 -1.649185
O 0.888201 3.057915 -0.710786
H -0.113073 3.019710 -0.553429
H 1.065913 3.903561 -1.137831
H 1.711662 -2.061381 -0.860701
H 0.616766 -2.749529 0.066967
O 1.816124 1.832188 1.515279
H 1.820376 2.380063 2.303398
H 1.585732 2.423177 0.762044
O 3.209459 -1.160900 -1.228588
H 3.613749 -0.802025 -0.403932
H 3.910871 -1.469271 -1.803914
O 3.857817 -0.034181 1.081688
H 3.255183 0.702954 1.279678
H 4.706703 0.170105 1.474476
O -4.039197 0.035174 -0.120822
H -4.996863 0.065333 -0.138270
H -3.717326 0.706592 0.511963
O -0.443132 -2.802053 1.709827
H -0.312834 -1.894828 2.019362
H -0.395201 -3.384412 2.469665
O -2.440398 1.750336 1.331920
H -2.677775 2.351601 2.047148
H -1.995084 2.319779 0.622615

[Mg(H2O)12]+, 6x
E=-1117.091849
Mg -0.407882 -0.858081 -0.618336
O -0.081751 -2.741527 0.183795
O -0.711659 0.972963 -1.582989
O -1.291501 -1.683070 -2.384880
O 1.384179 -1.019136 -1.666744
O 0.478611 -0.001124 1.150383
O -2.290715 -0.761594 0.231509
O 3.156731 1.094546 -0.590182
O -0.900738 3.360614 -0.311078
O 3.047072 -0.952662 1.193839
O 1.439617 2.627698 0.844535
O -2.990267 1.787563 0.792033
O -1.273975 -1.803344 2.583671
H -0.665881 -2.185913 -2.914832
H -2.066263 -2.235747 -2.252704
H -1.110869 0.906680 -2.452357
H -0.838903 1.882202 -1.248240
H 1.942856 -0.225204 -1.656879
H 1.943039 -1.732861 -1.232059
H -2.648620 0.141393 0.370726
H -2.303484 -1.192819 1.101175
H 3.361132 0.371354 0.043112
H 3.978095 1.338221 -1.022367
H 1.370999 -0.397112 1.292353
H 0.630640 0.959103 1.201411
H -0.966565 4.228665 -0.712818
H -0.030715 3.303790 0.134403
H 2.135462 2.234355 0.270525
H 1.887530 3.088182 1.556837
H 3.605178 -1.238644 1.927921
H 2.825488 -1.792525 0.652089
H -3.853374 2.174528 0.939760
H -2.391613 2.496549 0.515540
H -0.646283 -1.068100 2.576508
H -1.488888 -2.003596 3.495975
H 0.903003 -2.927735 0.193579
H -0.402195 -2.875211 1.089239

[Mg(H2O)16]+, 3x
E=-1422.797967
O 0.150998 -0.764473 -1.155473
Mg -1.177831 -2.283883 -0.596164
O -2.578988 -1.338144 0.469085
O -2.110833 -2.089157 -2.419430
O -1.161727 0.196403 -3.288617
O -1.942890 2.169374 -1.894771
O 2.672814 -1.895393 -1.300460
O 2.080348 -3.214701 1.009400
O 0.273604 3.599141 -1.497883
O -3.519186 1.242725 0.305983
O -2.233448 1.440442 2.687269
O -0.021896 0.767007 1.404846
O 2.075235 -0.563206 2.274962
O -2.707209 -1.255362 3.096534
O 1.637363 2.987938 0.744947
O 3.909334 1.287171 1.273602
O 4.606445 0.177629 -1.082280
H -0.093063 -0.295785 -1.985727
H 1.088739 -1.042327 -1.230070
H -1.905528 -1.270234 -2.934253
H -2.921394 -2.481049 -2.744892
H -0.884536 0.473195 -4.163110
H -1.529002 1.009056 -2.804435
H -2.658516 -1.427038 1.452856
H -2.999199 -0.482053 0.252190
H -4.445819 1.485597 0.260962
H -3.195971 1.485026 1.200061
H -1.187589 2.760152 -1.669521
H -2.473798 2.007992 -1.102798
H -2.626804 -0.303016 3.271432
H -3.344137 -1.620521 3.710687
H 2.736161 -2.501166 -2.042721
H 2.623900 -2.454088 -0.496191
H -2.117028 2.199268 3.261195
H -1.353085 1.235029 2.283860
H 0.698764 0.214499 1.804784
H -0.099899 0.451400 0.495838
H 2.223249 -1.473684 1.993209
H 2.836208 -0.023806 1.995283
H 1.034071 2.332611 1.124522
H 2.513648 2.586354 0.834442
H 0.862695 3.425327 -0.730937
H 0.311475 4.540432 -1.666831
H 1.137152 -3.404520 0.840468
H 2.447382 -4.001095 1.422023
H 4.291632 0.954859 0.434378
H 4.631643 1.630747 1.801360
H 4.039070 -0.591110 -1.242897
H 5.453836 0.007381 -1.492898

[Mg(H2O)16]+, 4x
E=-1422.821397
Mg 0.376267 0.255136 -1.950953
O -3.233625 1.329897 -0.710689
H -2.786531 2.192609 -0.624489
H -4.138130 1.509054 -0.974627
O -0.355808 2.722142 2.166809
H -0.410641 3.252813 2.963736

H 0.587309 2.494664 2.037786
O 3.511728 -1.107510 -0.468084
H 3.407605 -1.111246 0.494272
H 3.785088 -0.204342 -0.688046
O -0.533993 2.134988 -2.374778
H 0.149449 2.596958 -2.878233
H -0.775942 2.709486 -1.621196
O 0.128118 -3.812426 -0.642046
H 0.343358 -4.733176 -0.798513
H -0.839673 -3.707284 -0.698186
O -1.550316 -0.521462 -2.014309
H -2.200921 0.143992 -1.723798
H -1.813235 -1.350050 -1.578701
O -1.552869 3.462231 -0.179638
H -1.749824 4.400516 -0.197760
H -1.109026 3.273388 0.675913
O -2.459984 0.633860 1.950696
H -1.681619 1.196212 2.073308
H -2.807693 0.851160 1.071370
O -2.275880 -1.974341 2.066045
H -2.370911 -0.982572 2.099260
H -2.763867 -2.336813 2.807241
O 3.556460 1.745872 -0.867605
H 2.852544 1.635608 -1.554896
H 4.241193 2.288711 -1.269049
O 1.276004 -1.580853 -1.863808
H 0.899075 -2.441513 -1.619384
H 2.151990 -1.503644 -1.409045
O 2.737334 -0.843159 2.303777
H 1.910973 -1.354802 2.205571
H 3.219534 -1.206037 3.048196
O 2.127681 1.753358 1.504351
H 2.485269 0.964508 1.950470
H 2.740499 1.960909 0.772588
O -2.402483 -2.791953 -0.534248
H -2.496126 -2.539916 0.410774
H -3.236002 -3.175912 -0.812932
O 0.405871 -2.003426 1.475542
H -0.468174 -2.112075 1.893921
H 0.490165 -2.739475 0.844817
O 0.214568 0.414044 0.111295
H 0.216663 -0.456888 0.558223
H 0.909933 0.933441 0.566358

[Mg(H2O)16]+, 5x
E=-1422.824303
O -0.920367 -0.154157 -3.263740
O 0.698809 1.514468 -1.730354
Mg -0.110605 1.896452 0.156628
O 0.035939 -0.136086 0.550700
O -2.150054 1.591576 -0.239373
O 1.776143 2.386641 0.978607
O 2.180985 -1.530520 1.543858
O -1.965777 -1.249891 2.044018
O 3.107472 -0.035147 -1.654278
O 2.299819 -2.538911 -1.045879
O -0.182117 -2.562561 -1.867516
O -2.667739 1.384756 2.500501
O -3.238742 -0.410146 -1.846366
O -2.572004 -2.468860 -0.245803
O 3.897257 0.607826 0.884794
O 0.141222 -2.361862 3.296075
H -1.836680 -0.188844 -2.924866
H -0.974004 -0.085372 -4.218627
H -3.037814 -3.300560 -0.143056
H -2.436237 -2.095446 0.656901
H -2.635542 1.601361 0.604116
H -2.580785 0.941789 -0.822357
H 4.800811 0.857283 1.086076
H 3.839846 0.427748 -0.074472
H 0.118285 1.016024 -2.335537
H 1.541284 1.028469 -1.722370
H -4.150406 -0.360747 -2.138683
H -3.137818 -1.224496 -1.303262
H -0.914762 -2.602537 -1.233579
H -0.364124 -1.795398 -2.430851
H 1.343061 -2.593555 -1.326763
H 2.721526 -3.350040 -1.335059
H -1.794631 1.863186 2.507592
H -3.243261 1.868015 3.101444
H 2.564001 1.813274 1.054809
H 1.486515 2.621741 1.875431
H 0.972566 -2.131038 2.852122
H 0.358416 -2.688117 4.168736
H -1.339731 -1.655011 2.679519
H -2.333390 -0.432765 2.427310
H 2.867132 -0.984790 -1.546820
H 3.632368 0.035799 -2.454017
H 2.330399 -2.071453 0.747673
H 2.921133 -0.906451 1.587827
H 0.833886 -0.559779 0.924552
H -0.715578 -0.514909 1.051087
O -0.343896 3.956502 -0.311062
H 0.476656 4.445142 -0.434698
H -0.761464 4.315030 0.486819

[Mg(H2O)16]+, 6x
E=-1422.826137
O 1.850300 -0.195685 -2.824563
O 3.683627 -0.561704 -0.875200
O 3.273134 1.694017 0.534739
O 1.771748 0.797444 2.517415
O -0.315072 2.357064 3.365830
O -0.115346 0.194477 0.643895
Mg -0.366742 -1.780872 0.214799
O -0.457394 -3.845381 -0.320037
O -0.497499 -1.282542 -1.824021
O 1.692901 -2.147845 0.335333
O -2.460940 -1.739623 0.416779
O -2.004424 2.119159 1.062661
O -3.921837 0.473811 -0.284575
O -2.257136 0.894574 -2.393881
O -1.043847 3.066438 -1.377471
O 1.465115 2.378457 -1.562178
H 2.629374 -0.394840 -2.265094
H 2.130234 -0.267578 -3.738730
H 3.945175 2.326373 0.795737
H 2.791339 1.421355 1.355533
H 1.890299 -2.032281 1.315435
H 2.395433 -1.647274 -0.120079
H -4.875516 0.483127 -0.382676
H -3.525878 0.657291 -1.160171
H 0.344120 -0.984394 -2.220982
H -1.132789 -0.574826 -2.032483
H 4.576194 -0.911913 -0.867851
H 3.680285 0.264609 -0.337909
H 2.038096 2.262414 -0.786581
H 1.564505 1.568597 -2.083884

H -0.069361 2.843711 -1.392670
H -1.128399 3.959929 -1.715290
H -3.062340 -1.007621 0.176195
H -2.609985 -1.932648 1.348157
H -1.041007 2.369869 2.725641
H -0.666471 2.660804 4.202497
H 1.135585 1.308372 3.058265
H 1.792175 -0.140825 2.843986
H -1.817973 1.739927 -2.134455
H -2.500774 0.973199 -3.318256
H -1.745805 2.662891 0.297100
H -2.858310 1.724800 0.834872
H -0.834006 0.833458 0.822366
H 0.597664 0.401027 1.287826
H -0.993804 -4.140029 -1.059201
H 0.430514 -4.196577 -0.439028
O -0.485874 -2.395883 2.243741
H -0.420421 -3.338226 2.446737
H 0.323098 -1.976467 2.699663

[Mg(H2O)16]+, 6x, inner
E=-1422.818520
O 1.385599 0.476807 0.473893
Mg -0.441284 0.337279 -0.402453
O -1.334019 1.591205 1.097271
O -0.872515 -1.293447 0.915050
O 0.027674 -0.847711 -1.997086
O -2.255297 0.741883 -1.399703
O 0.237960 2.043266 -1.698431
O 1.741624 -2.754451 -1.380293
O -3.917946 -1.317290 -1.116083
O 0.234171 1.653973 3.196036
O -3.407461 -0.373219 1.408217
O -0.767211 -3.640709 -0.397047
O 2.896259 2.414571 -0.815542
O 3.226377 -1.464891 0.651584
O 4.713620 0.215811 -0.812144
O 0.934949 -1.006112 3.102876
O -1.882729 3.482074 -0.831255
H 5.216458 0.003434 -1.598889
H 4.194854 1.010777 -1.003001
H 3.207585 3.287922 -0.571823
H 2.094143 2.524849 -1.349225
H 0.116502 1.632736 -2.562465
H -0.435609 2.748225 -1.614913
H -2.207574 4.382314 -0.788142
H -1.775018 3.145704 0.074488
H -2.628826 1.630892 -1.406073
H -2.974974 0.077838 -1.477992
H -3.170733 -1.975429 -1.347993
H -4.697739 -1.609413 -1.603315
H -0.790975 1.672736 1.928356
H -2.166950 1.147448 1.334993
H 0.077243 1.992996 4.077345
H 0.603370 0.752768 3.285374
H -3.781907 -0.725972 0.570755
H -4.042821 -0.558196 2.102136
H -1.785156 -1.184017 1.248022
H -0.837850 -2.193561 0.499885
H 0.325884 -1.240057 2.382106
H 0.802475 -1.652066 3.797933
H -1.127824 -4.479301 -0.087246
H -1.435846 -3.286759 -1.068224
H 1.066207 -3.357848 -1.035187
H 2.319314 -2.525420 -0.636838
H 3.591600 -1.718027 1.500545
H 3.911903 -0.961880 0.157770
H 0.754503 -1.515396 -1.856586
H -0.762030 -1.381287 -2.240836
H 1.971903 -0.285804 0.677691
H 1.974868 1.218356 0.262040

[Mg(H2O)17]+, 6x
E=-1499.251254
Mg 0.530094 -1.764451 0.263199
O -1.392612 -1.782533 1.032241
O 0.143240 0.226460 -0.370533
O -0.141379 -2.533352 -1.558103
O 2.455135 -1.808370 -0.580135
O 1.350569 -0.868765 2.046326
O 0.957619 -3.605703 1.133378
O 2.193158 1.923323 1.665232
O -1.072466 -0.459854 3.392883
O -1.320300 1.560827 1.580428
O -3.726459 -1.089919 -0.369130
O -3.510602 1.607691 -0.195440
O -2.347641 -1.388227 -2.750950
O 0.457446 3.498952 -1.550957
O 2.288930 1.620947 -1.096761
O 4.379497 -0.025624 -1.902019
H 4.732781 -0.368720 -2.725937
H 5.133007 0.101666 -1.319466
H 3.013862 -1.135112 -1.001969
H 2.999378 -2.189456 0.176284
H 2.516564 1.831981 0.755283
H 1.969410 1.021627 1.941822
H 0.880170 -4.457955 0.689743
H 2.155828 -1.445781 2.207499
H 0.767011 -0.923235 2.824424
H 1.895999 -3.530887 1.479155
H 1.811284 2.392892 -1.471534
H 3.005797 1.323394 -1.670505
H -2.176890 1.670931 1.134892
H -0.900327 2.446054 1.616742
H -2.916450 1.669144 -0.982472
H -4.180614 2.289042 -0.272939
H -3.848235 -0.124966 -0.242941
H -4.544375 -1.523361 -0.117806
H -2.972174 -1.364111 -2.000664
H -2.788708 -1.847332 -3.468368
H -1.421686 -0.421110 4.283915
H -1.214355 0.406422 2.963186
H -1.476119 -1.501449 1.961072
H -2.228498 -1.588064 0.578328
O 0.271297 3.712872 1.320823
H 0.363719 4.530000 1.812934
H 1.047693 3.137961 1.543873
O -1.659057 1.380176 -2.103934
H -0.991035 0.888614 -1.584759
H -1.933637 0.753190 -2.783894
H -0.346756 3.124603 -1.926294
H 0.261710 3.745960 -0.633528
H 0.977160 0.695248 -0.625116
H -0.251689 0.712437 0.386507
H -0.890729 -2.169806 -2.070546
H 0.578788 -2.727629 -2.162109

[Mg(H2O)17]+, 6x, inner

E=-1499.263324

Mg	0.598451	0.429019	-0.326611
O	-1.293181	0.814652	0.299452
O	0.211934	-1.714723	-0.280068
O	1.305485	0.352831	1.654912
O	0.742734	2.558272	-0.459128
O	-3.354065	2.035728	-1.098167
O	2.235233	2.914350	1.846833
O	3.604581	2.805145	-0.559690
O	2.537698	0.335456	-1.063106
O	3.819217	-1.920824	-0.260263
O	0.016311	0.347442	-2.339593
O	1.913806	-2.753888	-2.040914
O	-2.690249	-0.198238	-2.632162
O	-2.449758	-2.167769	-0.755136
O	0.877761	-2.874381	2.103863
O	-0.539364	-0.713109	3.225830
O	-2.957552	-0.751886	1.685450
O	-4.970956	0.890901	0.944092
H	-5.664025	1.382680	1.384151
H	-4.593610	1.459922	0.257078
H	2.269515	3.386276	2.679825
H	1.999717	1.984032	2.037108
H	-3.259898	1.350912	-1.784926
H	-3.384737	2.886220	-1.538931
H	1.005386	2.942441	0.395266
H	1.397550	2.897111	-1.079387
H	3.413633	2.967364	0.380200
H	4.455827	3.195290	-0.760500
H	3.062594	1.152269	-0.952133
H	3.119401	-0.415371	-0.820794
H	3.398511	-1.904394	0.664676
H	4.762140	-2.070179	-0.131102
H	-0.898642	0.143105	-2.606578
H	0.626208	-0.087401	-2.939568
H	-3.144229	-0.469194	-3.432474
H	-2.749092	-0.947027	-1.998912
H	2.730988	-2.634310	-1.516524
H	1.934854	-3.643722	-2.395390
H	0.768882	-2.172920	-0.959114
H	0.427599	-2.166904	0.571444
H	-1.483267	-2.087900	-0.594692
H	-2.634155	-3.093896	-0.922389
H	1.004905	-3.813984	2.274016
H	1.802852	-2.472162	2.055947
H	-0.206943	-1.620129	3.145935
H	-1.459781	-0.737947	2.925746
H	-3.050078	-1.448878	1.021523
H	-3.774498	-0.215846	1.624679
H	0.591993	0.062694	2.292010
H	2.014526	-0.355502	1.724854
H	-1.870769	0.247300	0.859842
H	-1.871857	1.472149	-0.119188

[Mg(H2O)18]+, 6x

E=-1575.687121

Mg	1.759750	-1.007418	0.179236
O	0.436710	-2.202773	1.262119
O	0.090770	0.161471	-0.356186
O	1.530426	-2.268523	-1.472196
O	3.067391	0.121566	-1.012497
O	2.085520	0.348889	1.820708
O	3.398711	-2.058324	0.917838
O	0.976075	3.006799	1.452133
O	0.153580	-0.756418	3.550030
O	-1.598483	0.461787	1.827525
O	-2.132484	-2.996103	0.394026
O	-3.782738	-0.809738	0.589635
O	-0.918539	-3.378509	-2.048955
O	-2.057657	3.050422	-1.001205
O	0.538827	2.606795	-1.263599
O	3.006721	2.604060	-2.563827
H	3.350744	2.501036	-3.454052
H	3.569828	3.252861	-2.133384
H	2.953609	0.960355	-1.490083
H	3.843245	0.253763	-0.388973
H	1.017564	3.048758	0.483253
H	1.405959	2.167574	1.680298
H	3.817253	-2.782026	0.437564
H	3.088322	0.423756	1.820673
H	1.796312	0.010190	2.686436
H	4.104769	-1.367077	1.091617
H	-0.390429	2.903005	-1.401121
H	1.106552	2.811075	-2.015375
H	-2.444016	0.067548	1.555199
H	-1.745448	1.427842	1.931518
H	-3.847696	-0.274739	-0.238587
H	-4.666060	-0.866802	0.958491
H	-2.788564	-2.267378	0.430074
H	-2.490444	-3.719611	0.912572
H	-1.428848	-3.400772	-1.216896
H	-1.059333	-4.211283	-2.502183
H	-0.028596	-0.846720	4.485863
H	-0.575011	-0.252140	3.137078
H	0.370142	-1.952289	2.201376
H	-0.453322	-2.465081	0.976094
O	-1.624095	3.148040	1.825613
H	-1.925657	3.798380	2.460997
H	-0.631747	3.196816	1.786108
O	-3.603882	0.659234	-1.641657
H	-2.882543	0.161281	-2.107473
H	-4.294940	0.835324	-2.282606
H	-2.656868	2.316819	-1.198648
H	-2.105248	3.205529	-0.045434
H	0.316523	1.060484	-0.706634
H	-0.504549	0.302737	0.412109
H	0.724605	-2.750310	-1.739231
H	2.011452	-2.004647	-2.258969
O	-1.489915	-0.629896	-2.524321
H	-0.859022	-0.419168	-1.813727
H	-1.431025	-1.586313	-2.646250

[Mg(H2O)18]+, 6x, inner

E=-1575.692791

Mg	0.362753	-0.032918	0.290847
O	-1.681862	-0.094276	0.299659
O	0.171448	2.077571	0.515808
O	0.558571	0.119102	-1.810990
O	0.305926	-2.203784	0.126869
O	-2.388048	-2.716468	0.730149
O	1.454313	-2.357149	-2.389302
O	3.608916	-2.371879	-0.702316
O	2.449611	-0.083365	0.433930
O	2.210514	-3.546177	1.404274
O	3.568835	2.187410	-0.588281
O	0.375346	-0.317618	2.347486
O	2.344996	3.055898	1.702886
O	-2.006993	0.110132	3.392957

O	-2.316623	2.374545	1.895766
O	0.068480	3.330733	-1.934706
O	-1.503040	1.229095	-2.982235
O	-3.706465	0.722402	-1.312967
O	-4.304745	-1.894163	-1.175354
H	-4.662007	-2.524641	-1.800032
H	-3.708634	-2.371951	-0.579066
H	1.266604	-2.770506	-3.233304
H	1.163227	-1.419440	-2.435710
H	-2.564507	-3.110891	1.585673
H	-1.436465	-2.812956	0.550277
H	0.547359	-2.499584	-0.769578
H	0.936139	-2.667081	0.731785
H	2.995909	-2.470104	-1.457222
H	4.501828	-2.410233	-1.048326
H	2.524548	-3.481004	2.306417
H	2.951627	-3.327652	0.818294
H	2.927003	-0.865976	0.105704
H	2.948503	0.692196	0.097871
H	2.893635	2.239119	-1.347428
H	4.437974	2.299803	-0.988350
H	-0.455632	-0.163768	2.862130
H	1.118393	0.045677	2.831507
H	-2.283289	0.189894	4.306151
H	-2.272109	0.933769	2.933522
H	2.960244	2.938181	0.953701
H	2.486897	3.935372	2.054609
H	0.907724	2.508104	1.015493
H	0.097671	2.582980	-0.325617
H	-1.474052	2.331426	1.411171
H	-2.404541	3.272387	2.217519
H	0.148227	4.278181	-2.087350
H	0.965598	2.934639	-2.165444
H	-1.205656	2.138536	-2.824567
H	-2.368570	1.136976	-2.553474
H	-4.427195	1.290540	-1.038109
H	-4.055281	-0.193713	-1.371335
H	-0.265461	0.441944	-2.284023
H	1.249221	0.827700	-2.010548
H	-2.360650	0.395221	-0.204362
H	-2.110760	-0.904208	0.622803

[Mg(H2O)19]+, 6x
E=-1652.125536

Mg	1.741245	-0.034389	1.034760
O	0.614859	-1.407683	2.148992
O	-0.020999	0.205817	-0.173021
O	2.392181	-1.586767	-0.217777
O	2.815593	1.290353	-0.134420
O	1.041540	1.617496	2.211252
O	3.350878	-0.163778	2.341375
O	-0.567641	3.500711	0.730085
O	-0.851894	0.206848	3.783152
O	-2.279358	0.318942	1.431081
O	-1.302885	-3.273136	1.188685
O	-3.465172	-1.923396	0.164820
O	0.630691	-3.756946	-0.679022
O	-2.613872	1.825630	-2.173972
O	-0.022661	2.321356	-1.739289
O	2.437294	1.870964	-2.877985
H	2.503377	0.926553	-3.108110
H	3.048411	2.356124	-3.434260
H	2.728894	1.696888	-1.013111
H	3.218901	1.938633	0.499927
H	-0.203822	3.381619	-0.160288
H	0.000856	2.963501	1.305877
H	4.158503	-0.609390	2.058472
H	1.917264	2.093250	2.416680
H	0.612576	1.390572	3.055415
H	3.597433	0.795669	2.514112
H	-0.907422	2.229288	-2.154175
H	0.683930	2.341092	-2.401395
H	-2.849675	-0.398169	1.112116
H	-2.710489	1.167381	1.181093
H	-3.371315	-1.677002	-0.783214
H	-4.368864	-2.219065	0.290363
H	-2.120895	-2.874357	0.824232
H	-1.568012	-3.882727	1.880353
H	-0.059982	-3.785262	0.011164
H	0.890991	-4.659538	-0.869767
H	-1.263268	0.270741	4.645586
H	-1.540567	0.307894	3.098429
H	0.215219	-1.006050	2.942129
H	-0.048130	-2.023129	1.799706
O	-3.052897	2.711559	0.504307
H	-3.703352	3.344434	0.811396
H	-2.150582	3.116243	0.635877
O	-2.919491	-1.097440	-2.359272
H	-1.975133	-1.335928	-2.486308
H	-3.388824	-1.341461	-3.159303
H	-2.843422	0.888827	-2.218812
H	-2.966032	2.163753	-1.335458
H	0.008650	1.034553	-0.726376
H	-0.815455	0.279274	0.400731
H	1.921298	-2.432835	-0.285066
H	2.636605	-1.331628	-1.121296
O	-0.252246	-1.593642	-2.206533
H	-0.162706	-0.983446	-1.438930
H	-0.030686	-2.471513	-1.849501
O	2.307950	-0.900608	-3.023881
H	2.712668	-1.424097	-3.718118
H	1.363148	-1.148269	-2.985899

[Mg(H2O)19]+, 6x, inner
E=-1652.128093

O	-0.801939	-2.175670	0.089723
Mg	-0.012216	-0.211719	-0.263143
O	0.309198	-0.087434	1.784211
O	-1.996582	0.437192	-0.150030
O	0.733597	1.765879	-0.609966
O	-0.443767	-0.281275	-2.360740
O	1.854233	-0.975510	-0.637086
O	2.260327	1.432965	2.583314
O	-2.232015	-2.565274	-2.068918
O	1.791677	-0.409088	-3.740476
O	0.654350	3.461402	1.501348
O	-1.156184	2.566079	-2.401077
O	-2.638553	3.115415	-0.149834
O	3.880211	-0.238280	1.066093
O	2.459738	-2.107813	2.378361
O	1.556976	-3.612477	0.123977
O	3.161049	1.315979	-2.080654
O	-2.000045	0.842270	2.995445
O	-2.778720	-1.655512	2.050652
O	-4.130702	-1.321939	-0.322716
H	-1.363027	-2.447683	-0.689147
H	-1.397093	-2.132952	0.861166
H	3.044197	1.174168	2.075614

H	1.995659	2.328444	2.328756
H	-2.612573	-0.750393	2.390705
H	-2.955212	-2.209235	2.813756
H	-5.068992	-1.129296	-0.354513
H	-3.915880	-1.590408	0.589273
H	-2.785999	-0.103853	-0.328434
H	-2.300217	1.361081	-0.061418
H	-2.175589	3.204447	0.745843
H	-3.522622	3.478756	-0.025963
H	0.745507	2.408467	0.138750
H	0.187053	2.183930	-1.307273
H	-0.467300	0.195528	2.305891
H	1.060041	0.495495	2.100189
H	-1.953795	1.711529	2.494393
H	-2.005266	1.088444	3.926416
H	0.701174	4.421013	1.436699
H	-0.287991	3.259465	1.787095
H	-1.114801	3.216533	-3.103841
H	-1.785063	2.900194	-1.729275
H	1.950443	-1.940334	-0.563408
H	2.661614	-0.592207	-0.240990
H	-0.879488	0.550589	-2.600184
H	0.351362	-0.367404	-2.950431
H	1.972552	-0.314188	-4.675188
H	2.426454	0.149141	-3.252976
H	-1.817881	-1.832192	-2.544576
H	-3.101236	-2.251497	-1.784685
H	0.607371	-3.404359	0.148005
H	1.662490	-4.486803	-0.253202
H	4.825458	-0.341749	0.950396
H	3.560026	-0.972351	1.641072
H	1.629233	-1.620727	2.450303
H	2.265704	-2.833329	1.767905
H	2.437686	1.595728	-1.498940
H	3.709791	2.083376	-2.243089

[Mg(H2O)20]+, 3x
E=-1728.546637

O	-1.414764	0.333156	2.457452
O	-0.230979	-0.200119	0.038751
O	-0.093057	-2.780615	-0.824313
O	2.423945	0.888842	-0.034364
O	-1.507635	1.388050	-1.736360
O	-2.390037	2.756827	1.563850
O	-3.823718	1.771127	-0.423483
O	0.656573	2.731336	-1.905910
O	0.213937	3.941170	0.676018
O	3.497326	-1.524870	0.642634
O	2.571865	-2.600916	-1.736139
Mg	2.457347	1.553551	-2.038801
O	1.629267	-0.174003	-2.661468
O	1.120084	0.320973	3.503706
O	1.980549	-2.027300	2.760469
O	-2.303487	-2.450205	-2.354044
O	-3.278608	-1.037205	-0.335179
O	-0.533733	-3.248759	1.897008
O	-2.856089	-2.003982	2.271842
O	-0.765931	-0.482778	-3.657351
O	2.189311	2.599693	2.032537
H	-2.047814	-0.375964	2.666860
H	-1.051003	0.076097	1.586429
H	-0.377793	-3.288077	0.939986
H	0.275196	-2.870257	2.269475
H	-3.263485	-1.386069	0.572385

H	-3.633705	-0.136303	-0.306921
H	0.639117	3.382580	-2.615245
H	-0.249423	2.271338	-1.890748
H	-1.206771	0.729841	-1.069835
H	-2.399599	1.670770	-1.445683
H	-3.482679	-2.497990	2.801609
H	-2.030402	-2.544580	2.203013
H	1.499680	1.143525	3.169236
H	0.162256	0.364437	3.314033
H	0.743161	-2.952771	-1.280890
H	-0.830450	-2.877928	-1.458794
H	-1.275635	-1.269892	-3.401559
H	-1.245937	0.246678	-3.236517
H	0.146704	4.898649	0.660810
H	0.381737	3.655509	-0.238442
H	-4.670073	2.188668	-0.586159
H	-3.402800	2.217231	0.355280
H	-1.578521	3.210883	1.307274
H	-2.104038	1.959573	2.048342
H	2.880379	3.151557	2.404101
H	1.490375	3.195459	1.694661
H	2.909821	0.072102	0.220485
H	2.477753	1.516562	0.722307
H	0.682815	0.121365	0.023082
H	-0.201566	-1.134866	-0.263918
H	3.007459	-2.377619	-0.891483
H	3.132930	-3.224358	-2.200676
H	-2.774720	-1.932387	-1.655183
H	-2.932295	-3.067959	-2.728986
H	2.985739	-1.795551	1.450989
H	4.427987	-1.577448	0.871173
H	0.732982	-0.326639	-3.080813
H	2.019075	-1.047861	-2.478247
H	2.308103	-2.506055	3.524695
H	1.688717	-1.128233	3.084248

[Mg(H2O)20]+, 4x
E=-1728.561170

O	0.543304	-2.130506	-2.018279
H	-0.173114	-2.686660	-1.655695
H	1.377600	-2.449983	-1.629856
O	-1.556496	-3.537115	-0.827551
H	-2.385535	-3.036553	-0.972019
H	-1.720855	-4.438952	-1.110282
O	1.858527	2.977813	-2.439456
H	2.139119	3.672532	-3.042806
H	1.001255	2.617883	-2.787115
O	2.818925	-2.828654	-0.540214
H	3.510434	-2.131774	-0.563242
H	3.258144	-3.680095	-0.568631
O	1.217347	-1.681704	1.404026
H	1.669223	-1.259346	2.161852
H	1.862881	-2.192993	0.886921
O	3.494408	1.083570	1.301379
H	3.135775	0.742434	2.137930
H	2.834687	1.717816	0.975460
O	-0.107213	0.096543	-0.125221
H	0.370481	-0.539434	0.462167
H	-1.047170	0.115792	0.162334
O	-2.771169	0.151634	0.573900
H	-3.005772	1.097539	0.499169
H	-2.835328	-0.105910	1.517535
O	2.079437	-0.070240	3.408278
H	2.380120	-0.238543	4.301979

H 1.200310 0.380321 3.465748
O -0.743694 3.419084 1.801961
H 0.055324 3.276906 1.243178
H -0.607498 4.232930 2.290539
O -1.758474 -0.378145 -2.708360
H -2.187789 0.512868 -2.723008
H -2.417155 -0.997424 -2.354592
O 2.244044 0.254646 -2.182778
H 3.011689 -0.088439 -1.682041
H 2.421287 1.189792 -2.387642
O -2.470002 -0.837982 3.056975
H -3.074718 -1.061246 3.765537
H -2.060240 -1.680422 2.726469
O 4.265866 -0.575564 -0.539682
H 4.030210 0.038851 0.216435
H 5.190366 -0.435976 -0.750687
O 1.273839 2.539083 0.265209
H 1.504245 2.915601 -0.604011
H 0.792773 1.715103 0.062127
O -1.205409 -2.878938 1.977887
H -1.438561 -3.241449 1.112084
H -0.285895 -2.576965 1.876693
O -2.930338 2.859271 0.239246
H -3.667892 3.460776 0.354546
H -2.192930 3.189694 0.793621
O -0.335047 0.991143 3.312777
H -1.115491 0.414441 3.320696
H -0.573654 1.827878 2.889071
O -2.491065 2.181811 -2.552538
H -2.587771 2.501952 -1.641865
H -1.590547 2.432897 -2.819596
O -3.581097 -1.760707 -1.126552
H -3.454795 -1.047464 -0.459972
H -4.519805 -1.860310 -1.289964
Mg 0.202587 -0.108702 -2.202261

[Mg(H2O)20]+, 5x
E=-1728.557818
O -0.752658 -2.141161 -1.582236
Mg -1.099856 -1.737372 0.411007
O -2.832543 -0.654288 -0.006881
O 0.083143 0.007557 0.246686
O 0.558528 -2.778260 1.153244
O 0.162078 1.067967 -2.195364
O 0.999220 3.218087 -0.743563
O 2.765342 0.084306 0.850172
O 3.521984 1.874946 -1.054603
O 2.688210 0.262848 -2.969019
O 3.130255 -2.456671 0.109046
O -3.569883 1.189080 -1.959257
O -2.135642 3.128049 -0.884761
O -1.898498 -0.320869 -3.420227
O 1.920928 -2.458912 -2.325129
O -3.315055 0.210598 2.564890
O 0.623197 -1.420941 3.446381
O 2.118281 1.099499 3.289621
O 0.777070 3.243980 2.195756
O -1.447628 1.962723 1.532855
H 0.162442 -2.278533 -1.894241
H -1.215322 -1.626806 -2.267271
H 2.454633 -2.573026 -1.512588
H 2.131070 -3.192806 -2.905921
H -4.015554 0.149019 3.222140
H -2.654046 -0.512177 2.771625

H -2.672515 0.169900 -3.078368
H -2.057987 -0.510092 -4.346147
H 0.178032 1.986284 -1.869146
H -0.602543 0.892519 -2.764281
H -1.408349 3.686514 -1.168283
H -1.916839 2.818169 0.014660
H 0.091009 0.358778 -0.677880
H 1.024231 -0.006235 0.526392
H 2.756377 0.427693 1.764602
H 3.145552 0.778277 0.270125
H -0.141365 2.895335 2.140873
H 0.731435 4.119766 2.583796
H 0.713050 -2.388820 2.050982
H 1.437514 -2.851836 0.745976
H -3.128628 -0.064395 -0.724206
H -3.270610 -0.353956 0.810988
H 4.377061 2.285278 -1.191747
H 3.356271 1.268548 -1.822334
H -3.071861 1.991461 -1.663532
H -4.487894 1.442124 -2.068857
H -2.170657 1.566104 2.053857
H -0.954971 1.199997 1.170140
H 2.637966 -0.697385 -2.870407
H 1.763756 0.555828 -2.902414
H 2.628432 1.277466 4.081354
H 1.678979 1.934261 3.027447
H 1.921802 2.973617 -0.922065
H 0.945978 3.280650 0.221616
H -0.342302 -1.267262 3.385696
H 1.031836 -0.541665 3.457447
H 3.166940 -1.526439 0.430905
H 3.913031 -2.912002 0.422238
O -2.229991 -3.440761 0.951820
H -2.609217 -3.142261 1.796933
H -1.659187 -4.185637 1.172359

[Mg(H2O)20]+, 6x
E=-1728.558482
O 2.182441 -2.050186 0.710047
Mg 0.793570 -1.698325 -0.781587
O 1.477503 -3.405556 -1.757109
O 2.115593 -0.365398 -1.679525
O -0.109631 -0.050975 0.262303
O -0.651173 -1.373591 -2.262331
O 0.655572 0.190174 2.773394
O -1.355348 1.970832 2.923330
O -2.880853 -0.277329 0.128014
O -2.845591 -2.649533 -1.243953
O -3.150943 -0.243773 2.800450
O -1.161479 -1.941364 2.916455
O 4.113280 0.769822 -0.040169
O 3.163985 3.234875 0.175612
O 0.573280 2.511733 -0.465662
O -1.721340 3.553192 0.454234
O -3.278218 2.159333 -1.251140
O 3.374260 -0.176524 2.451439
O -1.695901 1.075755 -3.231484
O 0.944415 1.805433 -3.111732
O -0.528858 -2.999478 0.311247
H 2.137087 -3.024562 0.906118
H 2.533079 -1.574040 1.480607
H 1.351482 2.264457 -3.848192
H 0.019811 1.608226 -3.354415
H 3.794874 0.175951 1.643495

H	4.069430	-0.382438	3.078020
H	0.139805	0.988406	3.006365
H	1.607421	0.316373	2.899697
H	3.320371	3.868799	0.875522
H	2.201871	3.103641	0.095540
H	0.174723	-0.021313	1.214678
H	-1.086705	-0.146156	0.257885
H	-3.198102	0.532745	-0.302520
H	-3.165109	-0.245288	1.068323
H	-0.829522	3.332003	0.100008
H	-1.747925	4.498812	0.610805
H	-1.001592	-0.522131	-2.566577
H	-1.420122	-1.964220	-2.171277
H	2.852844	-0.020790	-1.141477
H	1.826265	0.337561	-2.281045
H	-3.881879	-0.443723	3.386581
H	-2.459144	-0.950736	2.927291
H	3.892446	1.729709	0.030168
H	5.005060	0.703735	-0.386998
H	0.732564	2.465009	-1.425897
H	0.384350	1.584185	-0.201612
H	-0.958281	-2.409809	2.089706
H	-0.384460	-1.390817	3.094587
H	-4.136714	2.548522	-1.427639
H	-2.800473	2.771350	-0.645422
H	-2.086583	1.337535	3.000542
H	-1.569683	2.545051	2.177425
H	-2.203472	0.941451	-4.034282
H	-2.284544	1.525622	-2.590084
H	-3.641369	-3.138403	-1.455129
H	-3.088874	-1.859454	-0.722768
H	1.526695	-4.102419	-1.032269
H	2.340294	-3.396425	-2.188302
H	-1.324638	-3.298010	-0.164446
H	0.067905	-3.808064	0.468670

[Mg(H2O)20]+, 6x, inner
E=-1728.563973

O	0.231000	-2.325461	-0.268120
Mg	-0.256632	-0.294746	0.247266
O	1.731440	-0.030576	0.801336
O	-0.825745	1.668827	0.883712
O	-0.580933	-0.883936	2.285077
O	-2.244148	-0.632306	-0.160055
O	0.155899	0.235613	-1.716238
O	0.902769	-3.339827	2.054270
O	-3.168744	-0.940967	2.791702
O	-3.319556	0.868419	-2.197916
O	-1.890765	-1.091324	-3.353741
O	-2.193736	-3.120614	-1.337899
O	-0.993494	2.386734	-2.586531
O	0.022842	3.862530	-0.444254
O	2.515495	2.323052	2.022816
O	0.313355	1.575372	3.381083
O	2.806582	0.140062	-2.507146
O	3.828035	2.104015	-1.076349
O	2.709637	-2.375697	-1.548053
O	3.376236	-2.235743	1.114703
H	0.468136	-2.822827	0.564319
H	1.015315	-2.392746	-0.845815
H	-1.938748	2.199689	-2.482687
H	-0.755718	3.116372	-1.992473
H	2.855162	-1.487778	-1.948430
H	2.960833	-3.029302	-2.203151

H	4.292175	-2.231467	1.396792
H	3.363648	-2.434942	0.159822
H	2.362420	-0.743488	1.006972
H	2.114707	0.804515	1.133493
H	2.309410	2.916459	1.227793
H	3.438481	2.495843	2.239752
H	-0.550818	2.482736	0.396397
H	-0.527507	1.799704	1.806147
H	1.094452	0.246161	-1.988324
H	-0.247962	1.086140	-2.063960
H	3.248912	0.870509	-1.993935
H	2.916947	0.349069	-3.436859
H	4.462503	2.739137	-1.420790
H	3.084907	2.649727	-0.696527
H	-0.110458	4.769104	-0.146480
H	1.014071	3.686299	-0.337669
H	0.090007	2.016839	4.201719
H	1.190931	1.907906	3.092265
H	-2.495781	-1.524115	-0.453972
H	-2.756789	-0.010087	-0.712884
H	-0.199364	-0.206392	2.867670
H	-1.537697	-0.964684	2.538690
H	-3.638550	-1.050030	3.618249
H	-3.518819	-0.142247	2.353012
H	0.466129	-2.621787	2.533548
H	1.848718	-3.138942	2.066572
H	-1.283905	-3.146920	-0.994763
H	-2.586840	-3.984762	-1.209876
H	-4.245479	0.995817	-2.408215
H	-2.963912	0.169585	-2.795593
H	-1.058189	-0.796206	-2.958180
H	-2.088208	-1.929558	-2.913060
H	-2.748702	1.636853	1.187255
O	-3.653250	1.436386	1.472532
H	-4.048172	2.260301	1.758569

D1 minima, ADC(2)/aug-cc-pVDZ

[Mg(H2O)3]+, 3x
E=-428.2178791192

O	1.4473174	1.4277961	-0.3253374
Mg	0.0031651	-0.0033894	-0.3223923
O	0.5130988	-1.9713841	-0.3195454
O	-1.9560393	0.5401091	-0.3123409
H	-2.5045004	0.6871553	0.4699257
H	-2.5122644	0.7021132	-1.0862046
H	1.8554468	1.8324675	0.4519049
H	1.8456238	1.8389615	-1.1042241
H	0.6561296	-2.5305093	-1.0949455
H	0.6534186	-2.5239959	0.4609818

[Mg(H2O)4]+, 3x
E=-504.5048006894

O	-0.4648674	1.5644809	-0.3711535
Mg	0.7259214	0.0076572	0.1629221
O	-0.4536350	-1.5584326	-0.3677767
O	-2.6161892	-0.0045939	0.4478087
H	-1.4055795	1.3715395	-0.1841484
H	-0.4183046	2.1222927	-1.1586022
H	-1.3959971	-1.3724265	-0.1822291
H	-0.4016029	-2.1212951	-1.1512575
H	-3.5638726	-0.0084700	0.2371458
H	-2.5787110	-0.0037381	1.4204615
O	2.4593035	0.0020868	1.2086288

H 3.3585011 0.0004894 0.8541803
H 2.5261973 -0.0010125 2.1729531

[Mg(H2O)4]+, 4x
E=-504.5182080785
O -0.0015974 1.5334500 0.9094636
Mg 0.0002885 -0.1103683 -0.2557201
O -1.9485025 -0.0367125 -0.8682196
O 0.0017940 -1.5578840 1.1694771
H 0.0022505 1.3810383 1.8814666
H 0.0006058 2.4997585 0.7835298
H -0.7671684 -1.6124545 1.7702252
H 0.7661975 -1.6094151 1.7764543
H -2.6995386 0.3707654 -0.4125671
H -2.2802559 -0.3860842 -1.7082106
O 1.9475685 -0.0361595 -0.8698075
H 2.2806780 -0.3859335 -1.7090271
H 2.6977100 0.3698874 -0.4115704

[Mg(H2O)5]+, 3x
E=-580.7930318790
O 1.0818692 0.3808559 0.2600333
Mg -0.5447734 -0.7392041 0.0995769
O -1.7206925 0.4790552 -0.9878923
O -1.2095267 -2.2490927 1.2771258
O -0.8867853 2.4807334 0.5631030
H 0.9183477 1.3164490 0.4594007
H 2.0191232 0.2628127 -0.0417278
H -1.7058709 1.3858650 -0.6055899
H -2.4888218 0.3818635 -1.5633911
H -0.9408015 3.4501019 0.5314416
H -1.2420620 2.2337721 1.4397779
H -1.1699404 -3.2077434 1.1584089
H -1.6012063 -2.0716417 2.1429422
O 3.5485695 -0.0855107 -0.5247188
H 3.8925389 0.0284080 -1.4206168
H 4.3214505 -0.0621862 0.0549955

[Mg(H2O)5]+, 4x
E=-580.8139653569
O -1.1747116 1.4521111 1.4294021
Mg -0.7405107 0.3142724 -0.1953709
O 0.9321290 0.8605441 -1.1663842
O -2.2642771 -0.8594139 -0.8484245
O 0.1836530 -1.2311721 0.7350159
H -1.7634749 2.1992676 1.6095906
H -0.6543390 1.2963803 2.2379987
H 0.0947035 -1.2926889 1.7136037
H 1.1564249 -1.2820602 0.5931808
H -2.3675490 -1.8077268 -0.6690248
H -2.9241215 -0.6197341 -1.5164910
H 1.8017901 0.5157056 -0.8548949
H 1.0722811 1.4488468 -1.9184094
O 2.8533458 -0.4864695 0.1119186
H 3.1702930 -0.0999128 0.9480078
H 3.6089702 -0.9869095 -0.2364162

[Mg(H2O)5]+, 5x
E=-580.8260919155
O 0.0797562 0.0209223 2.0828533
Mg 0.0009577 0.0006513 0.0481866
O 1.7480698 -1.0821699 -0.0215507
O -1.7519807 1.0757745 0.1109964
O -0.9692386 -1.5129688 -0.9352469

H 0.7132825 -0.4701803 2.6339862
H -0.4183670 0.6023038 2.6762129
H -1.8065318 -1.4429211 -1.4143618
H -0.6757161 -2.4319424 -1.0052149
H -2.6515518 0.7935019 0.3700901
H -1.8751446 1.9540806 -0.3144578
H 2.1820942 -1.5291371 -0.7751351
H 2.2321878 -1.3985178 0.7742943
O 0.8954839 1.5002549 -1.0008783
H 0.4497856 2.2817679 -1.3705675
H 1.8472889 1.6376911 -1.1166120

[Mg(H2O)6]+, 3x
E=-657.0872766274
O 1.0160872 2.2296967 0.3411790
Mg 0.8717990 0.2951476 -0.0335912
O 0.7288141 -0.4694108 -1.8657766
O 0.0509057 -1.0202076 1.2340862
O -1.4051889 -1.8900693 -2.2312841
O -2.4512706 -1.3368129 0.4048770
O -1.0914433 3.7240888 0.3672496
H 0.4572446 -1.6303215 1.8615572
H -0.8966170 -1.2754291 1.1027219
H 1.7900475 2.7053376 0.6650304
H 0.2373539 2.8648108 0.3309374
H -0.0935057 -0.9941006 -2.1107950
H 1.4580481 -0.7990333 -2.4050252
H -1.9932730 -1.8361631 -2.9969497
H -1.9795254 -1.8154853 -1.4467081
H -3.2054341 -1.7263913 0.8792087
H -2.6643892 -0.3822532 0.3230760
H -1.2508668 4.4567114 -0.2454221
H -1.8717890 3.1508812 0.2883307

[Mg(H2O)6]+, 4x
E=-657.1070903969
Mg -0.0000747 0.0002245 -0.1461909
O -1.2557985 -1.1533896 -1.1739911
O 1.4543687 -0.8913066 0.8797464
O -1.4544357 0.8922937 0.8787344
O 1.2558930 1.1527479 -1.1745984
O 3.5902302 0.3298467 -0.1658417
H -1.1662077 -1.8397153 -1.8520990
H -2.2231415 -1.0417467 -0.9826138
H 1.4988238 -1.5731261 1.5669509
H 2.3834503 -0.6093850 0.6741797
H 2.2231397 1.0410791 -0.9830873
H 1.1668635 1.8402361 -1.8515749
H 4.1434311 0.9270427 0.3793211
H 4.2320801 -0.1617163 -0.7192890
H -2.3832446 0.6091701 0.6736753
H -1.4995640 1.5732747 1.5667291
O -3.5901017 -0.3298882 -0.1665085
H -4.1435053 -0.9283016 0.3771430
H -4.2317218 0.1627859 -0.7192843

[Mg(H2O)6]+, 5x
E=-657.1205247051
O 2.1196857 -1.2571436 0.5924119
Mg 0.5437260 -0.1632240 -0.1595487
O 0.0627000 -1.6360592 -1.4853252
O 1.8406061 1.3327074 -0.6203295
O -0.4787377 -0.2276779 1.5876681
O -0.9689153 1.0430726 -0.8992582

O -2.9560254 0.6939293 1.0000045
H -1.4202690 0.0418860 1.6540804
H -0.1597581 -0.4652974 2.4669026
H 1.5923028 2.2368308 -0.8692013
H 2.7960863 1.3475403 -0.4264726
H 2.1651586 -2.1703550 0.9293399
H 2.9990358 -0.8660418 0.7857952
H -0.8283521 -1.6933506 -1.8791928
H 0.5952827 -2.3286221 -1.9045449
H -1.8022593 1.0836912 -0.3780748
H -1.2574155 1.0118523 -1.8292899
H -3.4763059 1.3895971 1.4351695
H -3.6143896 0.0421585 0.6995628

[Mg(H2O)6]+, 6x
E=-657.1314839576
O 1.1140499 0.7740217 1.5300710
Mg -0.0686619 0.0025721 -0.0019041
O -1.2029013 1.7511473 -0.0076216
O -1.2650030 -0.7837309 -1.5169552
O -1.3363426 -0.8203872 1.4331536
O 1.1872289 0.8472304 -1.4328388
O 1.0530903 -1.7523355 -0.0177135
H 2.0042956 -1.7066991 -0.2321532
H 0.9721515 -2.4474117 0.6532325
H -2.1429084 1.7467417 0.2478780
H -1.1101408 2.4596989 -0.6635729
H 1.1637466 0.6152925 -2.3738891
H 2.1312745 0.9745846 -1.2195354
H 1.1062970 1.7063999 1.7956602
H 2.0461489 0.4919832 1.5983168
H -2.2725399 -1.0046128 1.2367845
H -1.3006882 -0.6082553 2.3790427
H -2.1842624 -0.4866208 -1.6420711
H -1.2448969 -1.7103401 -1.8033133

[Mg(H2O)7]+, 3x
E=-733.3977422878
O -0.3416381 0.1306102 -1.3150717
Mg 1.0072413 0.6710878 0.0574821
O 1.0332104 -0.8615542 1.3791013
O 2.2637383 2.2415821 -0.2772801
O -0.4595193 -2.4972133 -1.2079159
O -1.3302409 -2.0825355 1.4652174
O -0.2952924 1.8766284 1.0625081
O -2.5653167 1.4032726 -0.2549054
H 3.1403637 2.1708547 -0.6883088
H 2.0808514 3.1868924 -0.1469387
H -1.2412807 0.4988555 -1.1999286
H -0.4544806 -0.8537232 -1.4235841
H 0.1709956 -1.3475258 1.4941979
H 1.7358268 -1.5251918 1.4033652
H -1.6039357 -2.6563707 2.2019008
H -2.0585388 -1.4190908 1.4023916
H -0.3693707 1.9758495 2.0222027
H -1.2264577 1.8270532 0.7211283
H -0.7419535 -3.1245872 -1.8870139
H -0.9767239 -2.7089642 -0.4098978
H -3.2282834 2.0250107 -0.6042933
H -3.0846829 0.7322606 0.2479009

[Mg(H2O)7]+, 4x
E=-733.3988490098
O 0.9776059 0.9448369 -0.0520124
Mg 0.1687040 -0.7491443 0.6540783
O -0.9123235 -1.8301196 -0.6554921
O 1.9694671 -1.6974736 0.4175123
O -1.4754704 0.2220579 1.3515088
O 3.6814753 0.2899073 -0.5030437
O -3.2593670 -0.6265392 -0.5983302
H 0.4164523 1.7427297 -0.1916841
H 1.8876979 1.0960658 -0.3536306
H -1.4655533 1.1791020 1.1200545
H -2.3158517 -0.0865496 0.9498998
H -1.8422426 -1.5382312 -0.8382183
H -0.6683398 -2.5082342 -1.2956932
H -4.1700995 -0.9649343 -0.6540561
H -3.2585494 0.1817205 -1.1655145
H 2.7478571 -1.1628738 0.1434699
H 2.2911081 -2.4163922 0.9830395
H 4.3765977 0.6664811 0.0578305
H 4.0948326 0.2181801 -1.3768330
O -1.1107819 2.5937648 0.0058685
H -1.7086659 2.4455763 -0.7666167
H -1.2378858 3.5320047 0.2329987

[Mg(H2O)7]+, 5x
E=-733.4135929063
O 1.4827143 1.2370386 0.8639306
Mg -0.1703828 0.3290885 0.0242969
O -1.8440589 -0.5805723 -0.7774191
O -0.9494603 2.1336046 -0.4777560
O -0.6069971 -0.6076044 1.7641707
O 1.0789938 -0.6813363 -1.2181117
O -2.3726767 -2.5295670 1.0632159
H -1.2155739 -1.3780850 1.7982501
H -0.1199559 -0.5616730 2.5965460
H -1.8765748 2.3567610 -0.6576874
H -0.4909282 2.9810233 -0.3047298
H 2.3586008 1.1359750 0.4375024
H 1.4495544 2.1582286 1.1893711
H 0.8918073 -1.5639913 -1.5734682
H 2.0446906 -0.5258327 -1.2579000
H -2.2064640 -1.3530615 -0.2837149
H -1.9972934 -0.7742633 -1.7161887
H -3.2441024 -2.7352688 1.4456859
H -2.0010775 -3.3959695 0.8076930
H 4.3725801 -0.0853738 -0.4952655
O 3.5893274 0.3807789 -0.8240765
H 3.9403193 1.0235806 -1.4594800

[Mg(H2O)7]+, 6x
E=-733.4232557355
O 1.7693145 1.0879324 -1.3160036
Mg 0.4743680 0.1397335 0.0238297
O -0.8377021 -0.8236457 1.3058439
O -0.4211639 1.9745487 0.4273068
O 1.4877958 -1.6501568 -0.2956118
O -0.8334803 -0.2303468 -1.5544579
O -3.1573380 -0.3840450 -0.0786307
H -1.7788179 -0.2246304 -1.2851719
H -0.7228649 -1.0154929 -2.1086773
H 1.3521758 -2.4681039 0.2077046
H 2.4141797 -1.6752517 -0.6020443
H 1.6652292 1.0627606 -2.2794910
H 2.7308306 1.0488018 -1.1594566
H -1.3223242 2.0170615 0.8107413
H -0.3819289 2.7230499 -0.1904775

H	-1.7832444	-0.7893835	1.0333962
H	-0.8280786	-0.7262768	2.2686152
H	-3.9316923	-0.9613315	-0.2077893
H	-3.5373544	0.4820211	0.1824440
O	1.8038972	0.5583908	1.5822030
H	2.6830161	0.1471310	1.6253242
H	1.8949041	1.4362169	1.9797810

[Mg(H2O)8]+, 3x
E=-809.6520874040

O	-1.1002490	1.9534353	-1.1761345
Mg	-1.5617656	0.3887235	-0.0423030
O	-1.0126503	0.5819378	1.8975174
O	-1.9320569	-1.5058503	-0.5035299
O	1.5159997	1.9163851	-1.2598665
O	0.8428081	-1.3564209	2.0530933
O	0.4088531	-2.6758974	-0.4827276
H	-2.7421244	-2.0124514	-0.6322076
H	-1.1393999	-2.1086968	-0.5665732
H	-1.6060460	2.6973714	-1.5231422
H	-0.1273771	2.0954634	-1.3398913
H	-0.4509072	-0.2076734	2.1109191
H	-0.3695936	1.3080987	2.0077691
H	1.0602580	-1.9684569	2.7730518
H	0.8069542	-1.9025959	1.2390400
H	0.5968852	-3.6055037	-0.6860837
H	0.9380803	-2.1431619	-1.1374670
H	2.1234151	2.5853126	-1.6142773
H	1.7295771	1.8346620	-0.3021689
O	1.6294753	-1.0412463	-2.2360555
H	2.4705903	-1.1575856	-2.7023377
H	1.6415526	-0.1254115	-1.9010626
O	1.6983910	1.4808733	1.5259748
H	2.3821317	1.8647916	2.0982170
H	1.8033244	0.5136361	1.6197909

[Mg(H2O)8]+, 4x
E=-809.6832841031

O	-0.2380144	-1.7106733	-0.3786988
Mg	-0.0009686	-0.0051758	0.5799588
O	0.2385345	1.7149860	-0.3516048
O	-1.6624729	-0.2261191	1.6633079
O	1.6558174	0.2001847	1.6735040
O	-2.6824604	-2.4495886	0.6075390
O	2.6768897	2.4420286	0.6581645
H	0.2843738	-2.1745004	-1.0812519
H	-1.0681040	-2.2100641	-0.2183480
H	2.1410333	-0.3568818	2.2993475
H	2.2289205	0.9837608	1.4628229
H	1.0669893	2.2131377	-0.1793764
H	-0.2798850	2.1873508	-1.0513941
H	2.8321822	3.2319141	1.2135846
H	3.4810013	2.3747361	0.1035771
H	-2.2357796	-1.0053884	1.4377572
H	-2.1483390	0.3201883	2.2980560
H	-2.8417627	-3.2479295	1.1496086
H	-3.4837756	-2.3721430	0.0502530
O	1.2603166	-2.8405313	-2.2549425
H	1.8030002	-3.6213303	-2.0786791
H	0.9390522	-2.9589979	-3.1593721
O	-1.2474137	2.8660191	-2.2252119
H	-1.8075120	3.6322234	-2.0395621
H	-0.9118800	3.0132829	-3.1202103

[Mg(H2O)8]+, 5x
E=-809.6987930617

O	-2.0034530	-1.0812958	-1.0238508
Mg	-0.7562282	0.2209210	-0.0209637
O	0.5083907	1.5225424	0.9728849
O	-2.3325097	1.3802052	0.5356399
O	0.2452468	0.6584305	-1.7167028
O	-0.1970688	-1.3296995	1.1470790
O	2.3656486	2.0462528	-0.9335326
H	1.0744986	1.1911115	-1.7196581
H	0.0348287	0.3824130	-2.6166920
H	-2.2934043	2.2811765	0.8951719
H	-3.2358121	1.2576400	0.1856242
H	-2.1832451	-1.9794981	-0.6746688
H	-2.8186254	-0.7912485	-1.4766060
H	0.6258968	-1.3780663	1.6681828
H	-0.6050010	-2.2173870	1.1202903
H	1.2525003	1.8762901	0.4306740
H	0.9082073	1.2810973	1.8275906
H	2.7492204	2.8893536	-1.2100824
H	3.1252877	1.4385661	-0.7326177
H	-1.5519113	-4.2898328	0.1105800
O	-1.8479113	-3.4554626	0.5045216
H	-2.5869917	-3.7088400	1.0782122
H	3.9034730	-0.2156287	0.6306519
O	4.0785399	0.1301236	-0.2611751
H	5.0363131	0.0503717	-0.3765342

[Mg(H2O)8]+, 6x
E=-809.7113086725

O	2.0249654	-1.6142520	0.7495738
Mg	0.8474607	-0.1281000	-0.1537597
O	-0.3293612	1.3398769	-0.9641089
O	0.1846801	-1.5417749	-1.5247982
O	1.5824083	1.3024060	1.1705517
O	-0.6047960	-0.6125676	1.2410568
O	-3.0793081	-1.1546050	0.2373886
H	-1.4986025	-0.9060741	0.9412199
H	-0.7639412	0.0589780	1.9195889
H	1.4637436	2.2566812	1.0255027
H	2.3978423	1.2137589	1.6995904
H	1.7481405	-1.9337472	1.6235028
H	2.9911576	-1.5150479	0.8023356
H	-0.6983973	-1.4435148	-1.9449297
H	0.2735595	-2.4978968	-1.3831209
H	-1.2413471	1.5101130	-0.6058954
H	-0.2895012	1.7021423	-1.8568171
H	-3.7341168	-1.7352379	0.6600372
H	-3.0174669	-1.4915602	-0.6839079
O	2.4061372	0.2864196	-1.4855758
H	3.1960758	0.7911818	-1.2332340
H	2.6469266	-0.2337789	-2.2657588
H	-3.1268460	0.7565367	0.2318762
O	-2.6268340	1.5928170	0.3000228
H	-3.2779133	2.3018448	0.2137708

[Mg(H2O)10]+, 3x
E=-962.2601827156

O	-0.8233398	0.3081121	-1.3426647
Mg	-0.4040143	1.7077676	0.0406816
O	-2.2632133	2.5898167	-0.1326297
O	1.4902455	1.2704028	0.5134223
O	-1.1548958	0.7104259	1.6500953
O	-1.9145775	-1.7244963	0.8904299

O	1.9267654	-0.8015165	2.2857461
O	0.1496240	-2.1783289	-0.9967460
O	2.7211833	-2.1294375	-0.0236242
H	-2.5086737	3.3484209	-0.6796317
H	-2.9743819	1.9204233	-0.2111983
H	-1.7780923	0.1296886	-1.4488061
H	-0.3752659	-0.5708484	-1.3411458
H	1.6208038	0.6001148	1.2204811
H	2.1876150	1.0687991	-0.1652723
H	2.4827579	-0.6951467	3.0796209
H	1.1320510	-1.2790259	2.6203254
H	-1.0126625	0.7925556	2.6049152
H	-1.3736085	-0.2470682	1.4936007
H	0.0587501	-2.8628417	-1.6759083
H	1.0791152	-2.2615656	-0.6719446
H	-2.0259062	-2.3465546	1.6311890
H	-1.1882530	-2.1134455	0.3529346
H	3.2248703	-2.9527290	0.0583670
H	2.5793891	-1.8232157	0.9007540
O	3.1568784	0.3499135	-1.3095186
H	3.3088001	-0.5712396	-1.0268022
H	4.0066657	0.6809783	-1.6275009
O	-3.4862838	0.0806203	-0.6486192
H	-3.2735864	-0.6526203	-0.0375706
H	-4.3461548	-0.1429879	-1.0309408

[Mg(H2O)10]+, 4x

E=-962.2914236820

O	-0.0861373	1.9395207	-0.5735177
Mg	0.0039103	0.0162099	0.1803817
O	0.0913964	-1.9107353	0.9160741
O	1.7299537	-0.2425563	-1.0243400
O	-1.2512068	-0.5905828	-1.3639501
O	-2.5923661	-2.3579607	0.2863286
O	2.5833737	2.4339399	0.0725532
O	-2.7378948	2.4699450	0.0918396
O	2.7284798	-2.4259490	0.2573043
H	0.6953668	2.5089420	-0.4612850
H	-0.8951100	2.4667077	-0.4250539
H	1.5673141	-0.4071222	-1.9644361
H	2.2529870	-1.0180213	-0.7037409
H	0.9010604	-2.4381364	0.7722046
H	-0.6908207	-2.4781095	0.8057776
H	3.2133834	-3.1780280	-0.1269336
H	3.2983904	-2.1357605	1.0044923
H	-1.7728260	0.0666805	-1.8573031
H	-1.9011479	-1.2588083	-1.0566147
H	-3.1835832	3.2267076	0.5169863
H	-3.3440712	2.2165072	-0.6366195
H	2.9998111	1.7771373	-0.5082803
H	3.2550352	3.1166132	0.2110535
H	-3.2926798	-3.0175763	0.1773526
H	-2.9618742	-1.6835709	0.8809290
O	1.2624557	0.6287458	1.7127810
H	1.8952544	1.3124134	1.4097048
H	1.8004970	-0.0113852	2.2126598
O	-1.7275345	0.2743354	1.3770482
H	-2.2564432	1.0434994	1.0508542
H	-1.5819068	0.4423085	2.3209633

[Mg(H2O)10]+, 5x

E=-962.2836926216

O	1.0057503	-2.2983443	-0.0753867
Mg	0.7627428	-0.2646898	-0.3598157
O	-1.1731019	-0.5481292	-0.9199667
O	0.3777878	-0.0509495	1.6472081
O	0.5991636	1.7780531	-0.5768897
O	2.2197503	-0.2205733	-1.7632152
O	-0.4883330	-2.6245616	2.1982920
O	-1.0356497	2.3238708	1.5596080
O	-2.5928867	-2.6568316	0.3487141
O	-3.0115594	1.4742934	-0.2305080
O	2.8217110	2.5159419	-2.0378214
H	-0.1766302	0.7204530	1.8792140
H	0.0014722	-0.8387102	2.0862481
H	2.6389040	0.6268778	-2.0341060
H	2.8906999	-0.9184519	-1.9114297
H	0.7355725	-2.6798525	0.7854228
H	1.8109487	-2.7631498	-0.3627176
H	-1.8140028	0.1835797	-0.8150258
H	-1.6436909	-1.3472655	-0.6109333
H	0.2137338	2.3076487	0.1458971
H	1.3158567	2.2873683	-1.0053851
H	-1.2067899	3.0012926	2.2274808
H	-1.8785238	2.1927586	1.0785404
H	-3.4539004	-2.2851327	0.6140078
H	-2.8243522	-3.4546705	-0.1535806
H	-3.3623736	2.0626604	-0.9196152
H	-3.8046389	1.0369430	0.1330646
H	-1.3375562	-2.8011469	1.7451547
H	-0.4917425	-3.1555683	3.0059369
H	3.6677315	2.7649547	-1.6293315
H	2.8258862	2.9588057	-2.9019554

[Mg(H2O)10]+, 6x

E=-962.2874183226

O	-2.805702	-0.4909363	-1.3529692
Mg	-1.3938439	0.0032470	0.1387721
O	-2.9420403	0.5345023	1.4376579
O	-0.0251923	0.2434412	1.6761640
O	-1.6271454	-1.9537577	0.8325869
O	-1.2413132	1.9974442	-0.4244913
O	0.0961049	-0.4903244	-1.1790404
O	1.7734841	-2.5553235	-0.5389728
O	2.4185204	1.2267932	0.9691377
H	0.6939755	-1.2515446	-0.9911981
H	0.6546123	0.2468941	-1.4966638
H	-0.4121688	2.2783348	-0.8703280
H	-1.9627919	2.5377243	-0.7874198
H	-2.4692114	-0.4841959	-2.2638040
H	-3.6311769	0.0255376	-1.3850649
H	-0.8895775	-2.3481062	1.3395138
H	-1.9477715	-2.6762925	0.2713817
H	0.8849333	0.5850778	1.5061830
H	-0.3116471	0.6291845	2.5139261
H	1.8880619	-3.2557052	-1.2060713
H	1.4386500	-3.0427526	0.2484688
H	-3.4343711	1.3675113	1.3277240
H	-3.5878845	-0.1131132	1.7617285
H	3.0496046	0.4908068	0.7330524
H	2.8937298	1.7871582	1.5979253
O	3.8600933	-0.7793276	0.0503372
H	4.7005688	-1.1313135	0.3708545
H	3.2868790	-1.5558122	-0.1086200
O	1.3056216	1.9760451	-1.4168647
H	1.8813230	1.9130160	-0.6225389
H	1.8020766	2.4737609	-2.0801685

[Mg(H2O)12]+, 3x
E=-1114.8029012661

O	-3.2625378	0.5038436	2.3278739
Mg	0.3555186	-1.9203364	0.4030815
O	2.1096410	-2.5812340	-0.1052168
O	0.5200370	-0.3993640	1.5912589
O	-0.4822429	-0.2484152	-2.7132895
O	1.3559648	2.7578253	0.4874125
O	-1.1098643	1.5322002	1.0632288
O	1.8235147	1.0634514	-1.8681970
O	3.5460033	-0.5760731	-0.4310846
O	2.8251299	0.9457776	1.8850560
O	-2.3719334	1.3858051	-1.4290353
O	-3.5632936	-0.7670748	-0.2800618
H	-3.9765532	0.8595130	2.8727551
H	-3.6951472	0.0333084	1.5947517
H	-0.9652425	-1.3928495	-1.6356727
H	-2.1039464	-1.5988550	-0.5626809
H	2.4819291	-3.4112468	-0.4251839
H	2.7916580	-1.8044668	-0.2678286
H	3.6059587	-0.0472168	0.3873293
H	3.1001740	0.0253963	-1.0701240
H	1.3790588	0.0191720	1.8502685
H	-0.1176734	0.3646812	1.4038016
H	2.0998230	1.5806619	-2.6534966
H	1.5585605	1.7573125	-1.2197089
H	2.4710909	1.7808140	1.4985260
H	3.3021794	1.2020235	2.6867087
H	0.4226910	2.6822667	0.7747755
H	1.4787539	3.6946957	0.2642406
H	-1.4814692	1.5693612	0.1522592
H	-1.9012097	1.3850060	1.6341347
H	-1.7300834	1.0147371	-2.0774227
H	-2.6461818	2.2340278	-1.8173734
H	-4.3636420	-1.1499862	-0.6681651
H	-3.3890079	0.0582506	-0.7926394
H	0.3566883	0.2387096	-2.5028860
H	-0.4789445	-0.3483432	-3.6833844
O	-1.1840966	-1.8958648	-0.7986710

[Mg(H2O)12]+, 4x
E=-1114.8368948129

O	2.0226860	1.9581165	1.0059806
Mg	0.2359403	2.1705714	0.1068982
O	0.4543906	0.8983687	-1.4004721
O	-0.9412862	3.5259667	-0.8265377
O	-1.2208447	1.4019053	1.2039923
O	-2.0328987	0.0021461	-2.0701806
O	-1.9376819	-2.2833268	-0.6252722
O	1.3683637	-2.3546872	2.0087215
O	-1.1633408	-1.0837642	1.8412705
O	-3.1922080	2.0867087	-0.6968009
O	2.2592112	-1.0875640	-1.5135033
O	3.2342617	-0.4034761	0.9099965
H	2.4407462	2.5938933	1.6022493
H	2.5551277	1.1154440	1.0240601
H	1.1315727	0.1887264	-1.5318389
H	-0.3940338	0.5362711	-1.7521591
H	-0.8688666	4.4812987	-0.9529851
H	-1.8907865	3.2628673	-0.8922557
H	-3.0148990	1.3145853	-1.2795927
H	-4.1391549	2.2687719	-0.7638297
H	-1.1948068	0.4335557	1.4978368
H	-2.1253674	1.5782860	0.8898321
H	-2.1771255	-0.1893489	-3.0098835
H	-2.0747117	-0.8843107	-1.6229648
H	-0.3133283	-1.5191872	2.0472349
H	-1.5388358	-1.6096649	1.1084864
H	-1.0582172	-2.7167621	-0.7518492
H	-2.5860017	-2.9969653	-0.7361103
H	1.6518910	-2.9417214	2.7260244
H	2.1269025	-1.7628145	1.8378727
H	3.0768114	-0.7195411	-0.0165200
H	4.1838307	-0.5236747	1.0629297
H	1.7305853	-1.9117615	-1.3353111
H	2.7533152	-1.2850005	-2.3295887
O	0.6912322	-3.1418498	-0.6353918
H	0.9672177	-3.1733583	0.3098442
H	0.9124783	-4.0207160	-1.0008809

[Mg(H2O)12]+, 5x
E=-1114.8574166056

Mg	-0.3721223	-0.3691226	-0.6932933
O	-2.0306457	-1.4696697	-0.2849979
O	-0.2644251	0.7603937	0.9789471
O	0.6858444	-2.0596767	-0.2620748
O	1.2500594	0.2605370	-1.7118594
O	-1.6450079	0.8327709	-1.7802594
H	-2.4788379	1.0535907	-1.3187491
H	-1.3696188	1.6283664	-2.2706816
H	-2.9008632	-1.0414153	-0.1930299
H	-1.9836117	-2.2548143	0.2979340
H	-0.9742776	0.8162051	1.6483257
H	0.3521286	1.5170319	1.0812318
H	1.4363609	1.1997651	-1.9320304
H	2.1054752	-0.1820802	-1.5196638
O	1.5878886	2.9912419	-1.8709928
H	0.7538273	3.3929505	-2.2090728
H	2.2864316	3.4121853	-2.4094623
H	1.6639056	-2.0400091	-0.2352332
H	0.3459602	-2.7381258	0.3491015
O	1.6809769	2.6780129	0.8759155
H	1.7214001	3.5106788	1.3683447
H	1.6904314	2.9472377	-0.0730874
O	3.2374687	-1.2141978	-0.5849691
H	3.5019645	-0.6159132	0.1577007
H	4.0481999	-1.6116671	-0.9275609
O	3.4990369	0.5483575	1.4145347
H	2.9947573	1.3710936	1.2633208
H	4.2952656	0.8137335	1.8919500
O	-3.8043028	0.6155654	-0.0058693
H	-4.7519134	0.7732124	-0.1105901
H	-3.6055812	0.7620072	0.9405965
O	-1.1193296	-3.5243627	1.2704225
H	-1.2341419	-3.3198732	2.2179388
H	-1.3639635	-4.4600257	1.1925781
O	-2.5620137	0.5973248	2.5344617
H	-2.5560066	-0.3346463	2.8410148
H	-2.7681810	1.0978823	3.3401364

[Mg(H2O)12]+, 6x
E=-1114.8636259537

Mg	-0.3290021	-0.9078692	-0.7175497
O	0.3223495	-2.6164162	0.3270640
O	-0.9479253	0.8344472	-1.6719262
O	-1.1342127	-2.0897236	-2.2378825
O	1.4934836	-0.8536069	-1.7526476
O	0.4810488	0.0284162	0.9449264

O	-2.1709892	-1.0154667	0.2308021
O	3.1552067	1.0698951	-0.6229780
O	-1.1414986	3.2345276	-0.4290578
O	3.0861671	-0.5590365	1.5923521
O	1.2498487	2.6807256	0.7734928
O	-2.9555551	1.4762269	0.9120156
O	-0.9417831	-1.8922275	2.6328210
H	-0.9215084	-1.9909754	-3.1861237
H	-2.1017561	-2.1687461	-2.2004820
H	-0.9935671	0.8455357	-2.6407864
H	-1.0409917	1.7629391	-1.3628709
H	2.1254111	-0.1485675	-1.4885235
H	1.4918156	-0.8643192	-2.7228802
H	-2.5597839	-0.1230829	0.3973920
H	-2.0803313	-1.3997498	1.1216428
H	3.4088772	0.5288327	0.1600914
H	3.9929087	1.3698789	-1.0059999
H	1.3616721	-0.3179528	1.2260643
H	0.5934250	1.0019239	1.0180913
H	-1.3190791	4.1101466	-0.7977650
H	-0.2680307	3.2956274	0.0149474
H	2.0285146	2.3827780	0.2563176
H	1.6158852	3.0435279	1.5973402
H	3.2649272	-0.1531000	2.4671686
H	3.4487146	-1.4622931	1.6689907
H	-3.8509696	1.7765691	1.1119100
H	-2.4953115	2.2439935	0.5284786
H	-0.4701028	-1.0504249	2.7613473
H	-1.1245825	-2.2242456	3.5232242
H	0.3480580	-3.5055029	-0.0478955
H	-0.0404777	-2.6879546	1.2338736

(H2O)6-
E=-457.6288051520

O	1.4893016	-0.3905881	1.3811923
H	0.5376145	-0.6751356	1.5130520
H	1.9962597	-0.8170355	2.0827603
O	0.8957558	2.0657535	-0.0838423
H	1.1718035	1.4498373	0.6166600
H	1.1602539	1.5899415	-0.8861268
O	1.7231071	-0.5594559	-1.3626750
H	2.4312879	-1.1166504	-1.7086202
H	1.7873664	-0.6439703	-0.3853235
O	-1.9000199	1.1942529	-0.0303014
H	-1.1112197	1.7726535	-0.0660800
H	-2.6611003	1.8052306	-0.0732222
O	-1.1275295	-1.2594456	-1.3689579
H	-0.1988791	-1.0297136	-1.5523960
H	-1.5528567	-0.3900964	-1.2286686
O	-1.0687826	-1.0665917	1.4409603
H	-1.1225564	-1.4192959	0.5258665
H	-1.5347002	-0.2148204	1.3091154
